# bSRWPSO-FKNN: A boosted PSO with fuzzy K-nearest neighbor classifier for predicting atopic dermatitis disease

**DOI:** 10.3389/fninf.2022.1063048

**Published:** 2023-01-16

**Authors:** Yupeng Li, Dong Zhao, Zhangze Xu, Ali Asghar Heidari, Huiling Chen, Xinyu Jiang, Zhifang Liu, Mengmeng Wang, Qiongyan Zhou, Suling Xu

**Affiliations:** ^1^College of Computer Science and Technology, Changchun Normal University, Changchun, Jilin, China; ^2^College of Computer Science and Artificial Intelligence, Wenzhou University, Wenzhou, China; ^3^School of Surveying and Geospatial Engineering, College of Engineering, University of Tehran, Tehran, Iran; ^4^Department of Dermatology, The Affiliated Hospital of Medical School, Ningbo University, Ningbo, China; ^5^School of Medicine, Ningbo University, Ningbo, Zhejiang, China

**Keywords:** swarm intelligence optimization, FKNN, feature selection, machine learning, atopic dermatitis

## Abstract

**Introduction:**

Atopic dermatitis (AD) is an allergic disease with extreme itching that bothers patients. However, diagnosing AD depends on clinicians’ subjective judgment, which may be missed or misdiagnosed sometimes.

**Methods:**

This paper establishes a medical prediction model for the first time on the basis of the enhanced particle swarm optimization (SRWPSO) algorithm and the fuzzy K-nearest neighbor (FKNN), called bSRWPSO-FKNN, which is practiced on a dataset related to patients with AD. In SRWPSO, the Sobol sequence is introduced into particle swarm optimization (PSO) to make the particle distribution of the initial population more uniform, thus improving the population’s diversity and traversal. At the same time, this study also adds a random replacement strategy and adaptive weight strategy to the population updating process of PSO to overcome the shortcomings of poor convergence accuracy and easily fall into the local optimum of PSO. In bSRWPSO-FKNN, the core of which is to optimize the classification performance of FKNN through binary SRWPSO.

**Results:**

To prove that the study has scientific significance, this paper first successfully demonstrates the core advantages of SRWPSO in well-known algorithms through benchmark function validation experiments. Secondly, this article demonstrates that the bSRWPSO-FKNN has practical medical significance and effectiveness through nine public and medical datasets.

**Discussion:**

The 10 times 10-fold cross-validation experiments demonstrate that bSRWPSO-FKNN can pick up the key features of AD, including the content of lymphocytes (LY), Cat dander, Milk, Dermatophagoides Pteronyssinus/Farinae, Ragweed, Cod, and Total IgE. Therefore, the established bSRWPSO-FKNN method practically aids in the diagnosis of AD.

## 1. Introduction

Atopic dermatitis (AD) is a chronic inflammatory skin disease accompanied by allergic reactions characterized by itchy eczematous skin-lesions and dry skin, which is common in childhood and influences at least 20% of children around the world (Rehbinder et al., 2020; Asano et al., 2022; Johansson et al., 2022). Considering the starting point of the atopic march with the development of food allergy, asthma, and allergic rhinitis, it is important to distinguish AD and intervene (Spergel, 2021). There are many diagnostic criteria for AD in the world, such as Hanifin & Rajka diagnostic criteria, Williams diagnostic criteria, International Study of Asthma and Allergy in Children (ISAAC) questionnaire, which depends on the subjective judgment of dermatologists (Williams et al., 1994; Williams, 1996). William’s criteria are the primary basis for diagnosing AD, which includes an itchy skin condition for the last 12 months and three minor criteria (Williams, 1996; Williams et al., 1996). Depending on the clinicians’ extensive experiences, some patients are missed or misdiagnosed. The sensitivity of diagnosis is enhanced when combined with serology results (Poto et al., 2022). Thus, a comprehensive evaluation of combination clinic symptoms with serology gradually attracts attention.

However, exploring such questions relies on vast amounts of relevant data. A large subject of research is medical information systems, which aid clinicians in providing quicker and more accurate medical diagnoses (Li C. et al., 2022; Liu S. et al., 2022; Zhuang et al., 2022). In this regard, manual analysis is impractical because it is time-consuming, inefficient, and error-prone for vast amounts of data. Consequently, it is essential to establish a machine learning model for studying AD, which will help AD staff better and more efficiently explore the factors and pathogenic features affecting AD. Moreover, some machine learning studies focus on AD. Gustafson et al. (2017) described a machine learning-based phenotyping algorithm that obtained higher positive predictive values (PPV) than previous low-sensitivity algorithms and demonstrated the utility of natural language processing (NLP) and machine learning in EHR-based phenotyping. Suhendra et al. (2019) proposed a machine learning algorithm that successfully combined a multi-class SVM classifier to classify and predict AD severity based on skin color, texture, and redness with an overall accuracy of about 0.86. Maintz et al. (2021) analyzed the association of 130 factors with AD severity based on a machine learning gradient boosting approach, cross-validated tuning, and multinomial logistic regression. It was demonstrated that the associations among AD patients identified in this study contribute to a deeper understanding, prevention, and treatment of AD disorders.

Guimarães et al. (2020) established a fully automated method based on a convolutional neural network (CNN) combined with multiphoton tomography (MPT) imaging to achieve AD morbidity prediction successfully. Li X. et al. (2021) used three machine learning models to analyze AI-assisted AD diagnosis and subclassify AD severity by 3D Raster Scanning Photoacoustic Mesoscopy (RSOM) images to extract features from volumetric vascular structures and clinical information. Jiang et al. (2022) developed a precise and automatic machine learning classifier on the basis of transcriptomic and microbiota data to predict the risk of AD. This method can accurately distinguish 161 subjects with AD from healthy individuals. Holm et al. (2021) developed two machine learning models to predict AD and explore the relationship between various immune markers in the serum of AD patients and AD disease severity based on clinically obtained biomarkers. Clayton et al. (2021) conducted a dermatological biopsy transcriptome profiling for AD. They performed cross-validation at different skin inflammation conditions and disease stages by using co-expression clustering and machine learning tools, ultimately revealing the impact of keratin-forming cell programming on skin inflammation and suggesting that perturbation of uniaxial immune signaling alone may not be sufficient to resolve keratin-forming cell immunophenotype abnormalities. Berna et al. (2021) constructed a machine learning framework for exploring the association between AD pathogenesis and low-frequency, rare alleles. However, because of the variety of factors that influence the physiological status of AD. Although the above scholars have conducted a series of explorations and studies for the prevention, diagnosis and treatment of AD, these extant studies are still inadequate for AD.

Therefore, to further explore the key factors affecting the physiological condition of AD, we propose a novel and effective feature selection method, bSRWPSO-FKNN, by combining the swarm intelligence optimization algorithm and machine learning techniques in this paper. While proposing the method to make the feature selection performance of the combination of particle swarm optimization (PSO) and the FKNN more outstanding, we first enhance the PSO. Thus, an improved variant of PSO combined with Sobol sequence population initialization (SOB), random replacement strategy (RRS), and adaptive weight strategy (AWS), named SRWPSO, is proposed for the first time. In SRWPSO, this study exploits the advantage of uniform distribution of low discrepancy sequences by the SOB, which enhances the diversity of the initial population and the traversal of the population space. It makes it easier for PSO to find the optimal particle position at the beginning. RRS and AWS are also introduced into PSO, which cooperate to make the PSO overcome the shortcomings of poor convergence ability and having fallen into local optimum. Moreover, the comprehensive performance of SRWPSO is demonstrated on 30 benchmark functions of CEC 2014, mainly including mechanism combination verification experiments, quality analysis experiments, comparison experiments with traditional algorithms, comparison experiments with famous variants, and comparison experiments with new peer variants. The benchmark function validation experiments show that SRWPSO, under the action of the three enhancement strategies, has the relatively best all-around performance among all the well-known algorithms involved in the comparison. Then, to apply SRWPSO to feature prediction, a binary version of SRWPSO is proposed in this paper, named bSRWPSO. Next, this paper combines bSRWPSO with FKNN to propose the bSRWPSO-FKNN model.

What’s more, to prove the feature selection performance of the model, the article firstly uses nine public datasets of the UCI to compare bSRWPSO with 10 other binary versions of the algorithm based on FKNN through the 10-fold cross-validation experiment. Then, this article also sets up a series of comparison experiments for the model on a medical dataset by the 10-fold cross-validation, including the comparison experiments of bSRWPSO combined with five classifiers, the comparison experiments of bSRWPSO-FKNN with other well-known classification models and the comparison experiments of 11 FKNN models combined with swarm intelligence algorithms. In this paper, we analyze the results of the experiments and demonstrate that bSRWPSO-FKNN has a significant core advantage over all the methods involved in the comparison experiments by combining the following evaluation indicators: Accuracy, Sensitivity, Matthews correlation coefficient (MCC), and F-measure. Finally, based on the bSRWPSO-FKNN and the medical dataset (AD), the key features affecting AD are extracted by 10 times 10-fold cross-validation experiments, mainly including the content of lymphocytes (LY), Cat dander, Milk, Dermatophagoides Pteronyssinus/Farinae, Ragweed, Cod, and Total IgE. The correctness and validity of the experimental results are also verified in the context of clinical medical practice. The main contributions of this study are summarized below.

1. An improved variant is proposed based on the PSO, named SRWPSO, which has stronger convergence in global optimization tasks.
2. A binary algorithm is proposed for solving discrete problems, named bSRWPSO.
3. A novel and efficient medical prediction method is proposed by combining bSRWPSO and FKNN, named bSRWPSO-FKNN.
4. The bSRWPSO-FKNN is successfully applied to AD prediction and provides a scientific approach to diagnosing AD and other disorders.

The rest of the paper is structured as follows. Section 2 describes the main work related to this article. Section 3 introduces the principle of operation of the original PSO. In section 4, the improvement process of the SRWPSO is presented. In section 5, the proposed bSRWPSO-FKNN is described. Section 6 sets up a series of benchmark function experiments to verify the advantages of SRWPSO. Section 7 sets up a series of feature selection experiments for bSRWPSO-FKNN and validates the potential of the method by the 10 times 10-fold cross-validation experiments. At last, section 8 reviews all the contents and guides the future work.

## 2. Related works

In recent years, feature selection technology based on swarm intelligence algorithms and machine learning techniques has gained wide attention in the field of medical diagnosis. Furthermore, many excellent machine learning methods have also been developed and applied to link diseases with various factors (El-Kenawy et al., 2020; Liu et al., 2020; Houssein et al., 2021; Hu et al., 2022a; Liu S. et al., 2021; Li Y. et al., 2022). For example, Hu et al. (2022b) presented a predictive framework based on an improved binary Harris hawk optimization (HHO) algorithm combined with a kernel extreme learning machine (KELM), which provides adequate technical support for early and accurate assessment of COVID-19 and differentiation of disease severity. Hu et al. (2022c) proposed a diagnostic model based on an improved binary mutation quantum grey wolf optimizer (MQGWO) and the FKNN techniques. They validated the model for hypoalbuminemia by predicting trends in serum albumin levels.

Liu et al. (2020) used the suggested COSCA method to optimize the two critical parameters of the SVM. As a result, they proposed a medical model that can self-directed the prediction of cervical hyperextension injury, named COSCA-SVM. Wu S. et al. (2021) combined an improved variant of the sine cosine algorithm (LSCA) and the FKNN techniques to propose a medical predictive model, named LSCA-FKNN, and successfully Its effectiveness has been validated on the disease in 3 medical datasets and lupus nephritis. Based on the proposed dispersed foraging sine cosine algorithm (DFSCA) and the KELM. Xia et al. (2022) established a new machine learning model called DFSCA-KELM. The medical diagnostic significance of the model was successfully confirmed by six public datasets and two real medical cases in the UCI library. Yang X. et al. (2022) proposed a feature selection framework called BSWEGWO-KELM and successfully verified the framework’s effectiveness by analyzing 1,940 records from 178 HD patients. Ye H. et al. (2021) proposed a predictive model that utilizes the HHO to optimize the FKNN, called HHO-FKNN. They successfully used this model to distinguish the severity of COVID-19, which one of the most hard cases in medicine (Li H. et al., 2021). Zuo et al. (2013) proposed an effective and efficient diagnostic system for Parkinson’s disease (PD) diagnosis based on particle swarm optimization (PSO) enhanced FKNN, which provides strong technical support for the diagnosis of PD.

Optimization methods are the oldest methods that can quickly bring feasible solutions using deterministic and gradient info (Cao et al., 2020b, 2021a,b) or without them (metaheuristic class). Also, as an emerging evolutionary computing technique, swarm intelligence algorithms have become the focus of more and more researchers. With the escalation of the solution problem, many different swarm intelligence optimization algorithms have gradually emerged to suit different problems. For example, there is ant colony optimization based on continuous optimization (ACOR) (Dorigo, 1992; Dorigo and Caro, 1999; Socha and Dorigo, 2008), particle swarm optimizer (PSO) (Cao et al., 2020a), different evolution (DE) (Storn and Price, 1997), sine cosine algorithm (SCA) (Mirjalili, 2016), HHO (Heidari et al., 2019b), grey wolf optimization (GWO) (Mirjalili et al., 2014), hunger games search (HGS) (Yang et al., 2021), Harris hawks optimization (HHO) (Heidari et al., 2019b), slime mould algorithm (SMA) (Li S. et al., 2020), Runge Kutta optimizer (RUN) (Ahmadianfar et al., 2021), weighted mean of vectors (INFO) (Ahmadianfar et al., 2022), colony predation algorithm (CPA) (Tu et al., 2021b), whale optimization algorithm (WOA) (Mirjalili and Lewis, 2016), bat-inspired algorithm (BA) (Yang, 2010), moth-flame optimization (MFO) (Mirjalili, 2015), wind-driven optimization (WDO)(Bayraktar et al., 2010), and so on. As time progresses, the drawbacks of the traditional swarm intelligence algorithm have also gradually emerged with the change of the problem, mainly including the slow convergence speed and low convergence accuracy of the algorithm when solving the problem. Therefore, many scholars have proposed a series of optimization variants based on the traditional algorithm. For example, there are hybridizing SCA with DE (SCADE) (Nenavath and Jatoth, 2018), chaotic BA (CBA) (Adarsh et al., 2016), modified SCA (m_SCA) (Qu et al., 2018), chaotic random spare ACO (RCACO) (Dorigo, 1992; Dorigo and Caro, 1999; Zhao et al., 2021), ACO with Cauchy and greedy levy mutations (CLACO) (Dorigo, 1992; Dorigo and Caro, 1999; Liu L. et al., 2021), hybridizing SCA with PSO (SCA_PSO) (Nenavath et al., 2018), double adaptive random spare reinforced WOA (RDWOA) (Chen et al., 2019), boosted GWO (OBLGWO) (Heidari et al., 2019a), fuzzy self-tuning PSO (FSTPSO) (Nobile et al., 2018) and so on. Furthermore, they have been well applied in many fields, such as resource allocation (Deng et al., 2022a), feature selection (Hu et al., 2022a; Liu Y. et al., 2022), complex optimization problem (Deng et al., 2022b), robust optimization (He et al., 2019, 2020), fault diagnosis (Yu et al., 2021), scheduling problems (Gao et al., 2020; Han et al., 2021; Wang et al., 2022), medical diagnosis (Chen et al., 2016; Wang et al., 2017), multi-objective problem (Hua et al., 2021; Deng et al., 2022d), solar cell parameter Identification (Ye X. et al., 2021), expensive optimization problems (Li J.-Y. et al., 2020; Wu S.-H. et al., 2021), gate resource allocation (Deng et al., 2020a, 2022b), and airport taxiway planning (Deng et al., 2022c).

Inspired by the foraging behavior of bird flocks, Kennedy and Eberhart (1995) proposed PSO, which is a stochastic search algorithm based on group collaboration developed by simulating the foraging behavior of bird flocks in 1995. Then, many famous scholars have researched and developed various variants of PSO based on different problems. Zhou Q. et al. (2021) propose a human-knowledge-integrated particle swarm optimization (Hi-PSO) scheme to globally optimize the design of the hydraulic-electromagnetic energy-harvesting shock absorber (HESA) for road vehicles. Nagra et al. (2019) put forward a mixed population algorithm (GSADMSPSO) that combines dynamic multi-swarm PSO (DMSPSO) and a gravitational search algorithm. Wang et al. (2021) proposed a dynamic modified chaotic PSO algorithm (DMO). Tu et al. (2020) proposed a novel quantum-inspired PSO (MQPSO) algorithm for electromagnetic applications. Zhen et al. (2020) proposed a hybrid optimization method (WPA-PSO) based on the wolf pack algorithm (WPA) and PSO. They proved that it has obvious advantages over a single algorithm in estimating and predicting the parameters of the software reliability model. The above improved PSO algorithms can have stronger capability to solve problems in one or several specific fields. However, there is no free lunch (Wolpert and Macready, 1997). In other words, the above methods gain enhancements in some problems while exposing drawbacks in other problems. Based on the above studies, we can conclude that PSO is an excellent swarm intelligence optimization algorithm, but there are many areas for improvement. Therefore, in this paper, an improved version (SRWPSO) is proposed for PSO and succeeds in making the classifier obtain better experimental results in feature selection experiments.

## 3. An overview of PSO

During the food search, PSO evaluates the fitness value of each individual at a location by a special evaluation function and uses this value to characterize the likelihood that the searching individual will find food there. Theoretically, the lower the evaluation value, the better the location. In addition, PSO introduces a memory mechanism for each searching individual to record that individual’s current optimal position. Then, the best position of all the independent individuals in the whole group of birds is used to determine the best foraging point for the whole group of birds, which is the global optimal position for the whole solution process. The PSO model is described in the section below.

Before updating the particle population, the PSO initializes a random population space *X*, as shown in Eq. 1.


(1) $$X_{m}^{n}=\left\{ \begin{array}{c} \left(X_{1,1},X_{1,2,},X_{1,3}\cdots X_{1,n}\right)\\ \left(X_{2,1},X_{2,2,},X_{2,3}\cdots X_{2,n}\right)\\ \vdots \\ \left(X_{m,1},X_{m,2},X_{m,3}\cdots X_{m,n}\right) \end{array}\right.$$

where $X_{m}^{n}$ represents an initial population space, *m* represents the number of individuals in the population, and *n* represents the number of dimensions of each individual.

For each particle, the corresponding position is a potential solution to the optimization problem, and each position’s fitness value is obtained by a special evaluation function. Then, it is made to compare with the recorded fitness value of the current individual, and if it is smaller than the previous fitness value, it is replaced. The optimal position of that individual is updated once. In each search process, the optimal position of each particle is recorded by *pB*, as shown in Eq. 2.


(2) $$pB_{i}=\left(pB_{i,1},pB_{i,2},pB_{i,3}\cdots pB_{i,dim}\right)$$

where *pB*_*i*_ records the best foraging position found by the *i*th particle in the current population, and *dim* indicates that each individual has *dim* dimensions.

The updating method is shown in Eq. 3. In the equation, *X*_*i*_(*t* + 1) represents the position of the individual after the current update process, *pB*_*i*_(*t* + 1) represents the best position obtained by the current individual after the *t* + 1th update, and *f*() represents the evaluation method for calculating the fitness value of each individual.


(3) $$pB_{i}\left(t+1\right)=\left\{ \begin{array}{c} X_{i}\left(t+1\right),iff\left(X_{i}\left(t+1\right)\right)<f\left(pB_{i}\left(t\right)\right)\\ pB_{i}\left(t\right),otherwise \end{array}\right.$$

For the whole particle population, the current search position of all particles becomes one of the candidates for the global optimal solution. The PSO will use the whole update process of the population to find the only global optimal target position and record it with Eq. 4, which is updated in the way shown in Eq. 5.


(4) $$gBest=\left(gBest_{1},gBest_{2},gBest_{3}\cdots gBest_{dim}\right)$$

(5) $$gBest=\left\{ \begin{array}{c} X_{i}\left(t+1\right),iff\left(X_{i}\left(t+1\right)\right)<f\left(gBest\right)\\ gBest,otherwise \end{array}\right.$$

where *gBest* indicates the global optimal position.

Of course, the key role of the PSO in updating the population of individuals is the movement vector of each particle, as shown in Eq. 6. Based on this vector, PSO can control the update direction and movement step of each particle, as represented by Eq. 7.


(6) $$V_{i}=\left(V_{i,1},V_{i,2},V_{i,3}\cdots V_{i,dim}\right)$$

(7) $$V_{i,j}\left(t+1\right)=V_{i,j}\left(t\right)+c_{1}\cdot rand\cdot \left(pB_{i,j}\left(t\right)-X_{i,j}\left(t\right)\right)$$

$$\;+c_{2}\cdot rand\cdot \left(gBest_{j}-X_{i,j}\left(t\right)\right)$$

where both *c*_1_ and *c_2_* are learning factors representing the movement of the particles toward *pB* and *gBest*, respectively. To make the particles move under certain limits for better merit search, the PSO constrains the displacement vector *Vε*[−*V*_*max*_, *V*_*max*_]. To deal with the displacement vector crossing problem, the researchers made the following settings, as shown in Eq. 8.


(8) $$V_{i,j}=\left\{ \begin{array}{c} V_{max},V_{i,j}>V_{max}\\ -V_{max},V_{i,j}<-V_{max} \end{array}\right.$$

Finally, the update formula for individuals is shown in Eq. 9.


(9) $$X_{i,j}\left(t+1\right)=X_{i,j}\left(t\right)+V_{i,j}\left(t+1\right)$$

**FIGURE 1:**
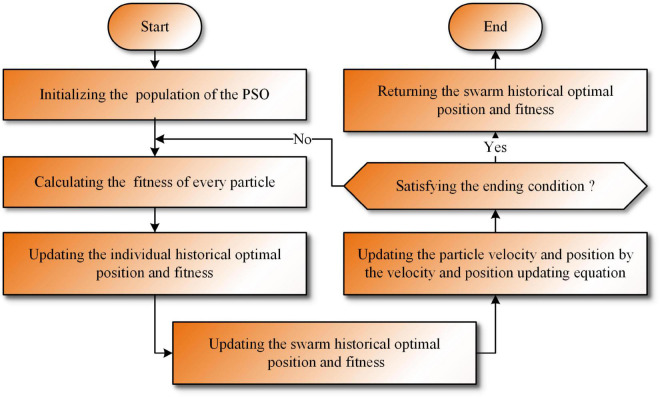
The workflow diagram of the PSO.

In summary, the workflow of the traditional PSO is shown in Algorithm 1 and Figure 1.

**Algorithm 1:** Pseudocode for the PSO

**Input:** The fitness function F (*x*), maximum evaluation number (*MaxFEs*), population size (*N*), dimension (*dim*) **Output:** the best location (*gBest*) Initialize a random population *X* Initialize the parameters: *FEs*, *t*, *V*_*max*_, *c*_1_, *c*_2_ Initializes the velocity vector: *V* = *zeros*(*N*, *dim*) Initializes the optimal position and grade of the current individual: *pB* = *zeros*(*N*, *dim*), *pB*_*score* Initialize position vector and score for the best location: *gBest*, *gBest*_*score* **While** (*FEs* < *MaxFEs*) **For i =** 1: size(*X*, 1) Keep each particle in the search space Calculate the fitness value for every search particle *FEs* = *FEs* + 1 Update the locations and scores of *gBest and pB*_*i*_ **End for** **For** *i* = 1: size(*X*, 1) **For** j = 1: size (*X*, 2) Updates the velocity vector *V*_***i**,**j***_ by Eq. 7 and Eq. 8 Update the location of particles by Eq. 9 **End for** **End for** *t* = *t* + 1 **End while** **Return** *gBest*

In summary, the time complexity of traditional PSO can be easily found and is mainly affected by initialization, population updating, and fitness value calculation. The population initialization is the most important component of the initialization phase and can be analyzed as *O*(*Initializing*) = *O*(*N* × *dim*). The population updating phase was analyzed as *O*(*Updating*) = *O*(*N* × *dim*), and the fitness value calculation phase was analyzed as *O*(*Calculating*) = *O*(*N* × *dim*). Thus, *O*(PSO) = *O*(*N* × *dim*) + *O*(*T* = (*N* × *dim*)) = *O*((*N* × *dim*) = (*T* + 1)). Here, *N* denotes the population size, *dim* denotes that each particle has *dim* dimensions, and *T* = *MaxFEs*/*M* denotes the number of iterations, which is determined by having the total number of evaluations (*MaxFEs*) and the number of evaluations(*M*) during each iteration.

## 4. The proposed SRWPSO

### 4.1. Sobol sequence

Based on studies related to metaheuristic algorithms, it can be found that the distribution of initial population individuals affects the convergence performance of metaheuristic algorithms to some extent (Rahnamayan et al., 2007; Kazimipour et al., 2014; Dokeroglu et al., 2019). Therefore, in this study, a more uniformly distributed low-difference random sequence (Sobol sequence) is adopted instead of the traditional pseudo-random method in an attempt to improve the diversity of the population and the algorithm’s traversal of the population space through low-difference sample points, thus enhancing the efficiency of the algorithm in finding the global optimal solution.

In addition, many scholars have also conducted related research on population initialization. For example, Yang X. et al. (2022) used a sinusoidal initialization strategy (SS) to initialize the population of the GWO algorithm and successfully enhanced the search capability of traditional GWO. Qi et al. (2022a) combined the Levy fight strategy and the traditional initialization method and proposed a Levy fight initialization method with better effect and successfully used to improve WOA. Arora and Anand (2019) used the Circle chaos method to initialize the population and improve the Grasshopper optimization algorithm(GOA). The initialization steps in this study are as follows.

Step 1: The initialized population space takes a range of values *Xε*[*lb*, *ub*]. *lb* denotes the lower bound of the population’s space, and *ub* denotes the upper bound of the population space.

Step 2: Sobol sequence generates random sample points with low variance properties *Sε*[0, 1].

Step 3: The initialization method is defined as Eq. 10.


(10) $$X_{i}=lb+S_{i}\cdot \left(ub-lb\right)$$

where *X*_*i*_ denotes the *i*-th particle in the population and *iε*[1, *N*].

Step 4: Repeating Step 3 *N* times based on population size *N*.

### 4.2. Random replacement strategy

To develop the population in a better direction, many scholars have tried to enhance the ability of traditional swarm intelligence algorithms for population updating by various methods. For example, the random replacement strategy has been effectively used in the literature (Gupta and Deep, 2018; Chen et al., 2019; Zhao et al., 2021). This strategy enriches the diversity of the population of individuals by replacing the position vector in the *j*-th dimension of the current individual with the position vector in the same dimension of the current swarm optimal individual. Thus, it improves the chance of exploiting the optimal individual.

Inspired by the method, this paper introduces the random replacement strategy into PSO, with the difference that this study transforms the object being replaced. In this improvement process, we combine the characteristic of PSO to record the current best position of each particle and achieve the improvement of the traditional PSO by replacing the position vector on the *j*-th dimension of the current best position of each particle with the position vector on the *j*-th dimension of the best individual of the population, as shown in Eq. 11.


(11) $$pB_{i,j}=gBest_{j}$$

During the search process, when the current optimal position of the population obtained by the algorithm approaches the global best position, we cannot exclude the possibility that it has excellent position vectors in some individual dimensions. Therefore, a probability parameter is introduced in the replacement strategy, as shown in Eq. 12.


(12) $$pB_{i,j}\left(t+1\right)=\left\{ \begin{array}{c} gBest_{j},a<C\\ pB_{i,j},\text{otherwise} \end{array}\right.$$

(13) C=tan(π⋅(rand-0.5))

(14) a=1-FEs/MaxFEs

where *C* denotes a Cauchy random number, and *a* is a decay factor that decays linearly from 1 to 0 as the number of evaluations increases.

### 4.3. Adaptive weight strategy

From the perspective of convergence speed and accuracy, the traditional PSO is easily trapped in the local optimum and lacks the ability to jump out of the local optimum in the middle and early stages of the updating process. In this paper, to remedy this deficiency, the adaptive weight ω is introduced into the velocity vector of the traditional PSO. The purpose is to improve the diversity of individuals in the population by increasing the perturbation capacity of the velocity vector, which facilitates the particles to explore and exploit the global optimum better, as shown in Eq. 15.


(15) $$\omega ={\left(1-\frac{FEs}{MaxFEs}\right)}^{\beta }$$

(16) $$\beta =1-C_{1}\cdot S/MaxFEs$$

where β stands for a perturbation parameter under the control of *C*_1_ and *S*, giving the possibility of jumping out of the linearly decreasing trajectory when ω decreases linearly from 1 to 0. *C*_1_, like *C*, denotes a Cauchy random number. *S* denotes an adaptive parameter with an initial value of 0.01, which is updated, as shown in Eq. 17.


(17) $$S=\left\{ \begin{array}{c} S/2,\text{if}gBest\text{updated}\\ S+1,\text{otherwise} \end{array}\right.$$

Therefore, the update of the velocity vector after the introduction of the adaptive weight ω can be expressed as Eq. 18.


(18) $$V_{i,j}\left(t+1\right)=\omega \cdot V_{i,j}\left(t\right)+c_{1}\cdot rand\cdot \left(pB_{i,j}\left(t\right)-X_{i,j}\left(t\right)\right)$$

$$\;+c_{2}\cdot rand\cdot \left(gBest_{i,j}-X_{i,j}\left(t\right)\right)$$

### 4.4. Implementation of SRWPSO

**FIGURE 2:**
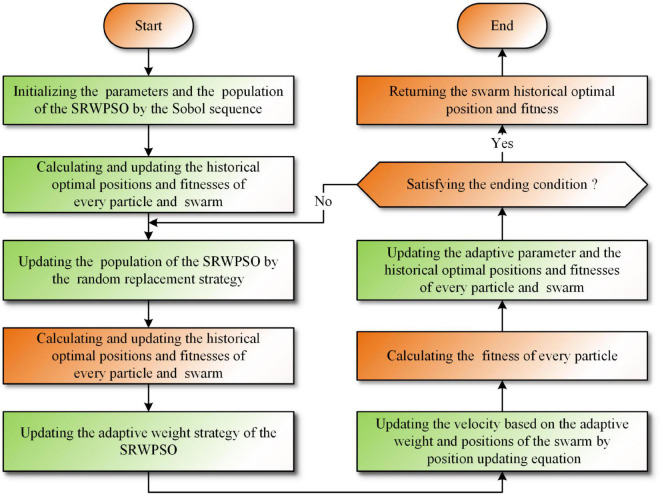
The workflow diagram of the SRWPSO.

In order to improve the overall performance of the PSO, this paper makes PSO combined with the three optimization strategies introduced above for the first time and proposes an enhanced PSO named SRWPSO. First, this study introduces the Sobol sequence in PSO for initializing the particle population to enhance the algorithm for population space traversal by improving the overall quality of the initial population. Next, in order to improve the possibility of moving to the global optimal position, this study introduces a random substitution strategy based on the optimal position of the current particles. Finally, an adaptive weight strategy is introduced to improve the algorithm’s ability to jump out of the local trap during the optimization search to increase the particle population’s perturbation ability by enhancing the displacement vector’s scalability. The specific framework of the enhanced SRWPSO is shown in Algorithm 2 and Figure 2.

**Algorithm 2:** Pseudocode for the SRWPSO

**Input:** The fitness function F (*x*), maximum evaluation number (*MaxFEs*), population size (*N*), dimension (*dim*) **Output:** the best location (*gBest*) Initialize a random population *X* by Eq. 10 Initialize the parameters: *FEs*, *t*, *V*_*max*_, *c*_1_, *c*_2_, *S* Initialize the velocity vector: *V* = *zeros*(*N*, *dim*) Initialize the optimal position and grade of the current individual: *pB* = *zeros*(*N*, *dim*), *pB*_*score* Initialize position vector and score for the best location: *gBest*, *gBest*_*score* **For i =** 1: size(*X*, 1) Keep each particle in the search space Calculate the fitness value for every search particle *FEs* = *FEs* + 1 Update the locations and scores of *gBest and pB*_*i*_ **End for** **While** (*FEs* = *MaxFEs*) **For i =** 1: size(*X*, 1) Updates the position of particles by Eq. 12 Keep each particle in the search space Calculate the fitness value for every search particle *FEs* = *FEs* + 1 Update the locations and scores of *gBest and pB*_*i*_ **End for** Update the adaptive weight ω by Eq. 15 **For** *i* = 1: size(*X*, 1) **For** j = 1: size(*X*, 2) Update the velocity vector *V*_*i, j*_ by Eq. 18 Update the location of particles by Eq. 9 **End for** **End for** **For i =** 1: size(*X*, 1) Keep each particle in the search space Calculate the fitness value for every search particle *FEs* = *FEs* + 1 Update the locations and scores of *gBest and pB*_*i*_ Update the adaptive factor *S* by Eq. 17 **End for** *t* = *t* + 1 **End while** **Return** *gBest*

Analyzing the above workflow, we can find that the complexity of SRWPSO is mainly determined by the population size (*N*), dimension size (*dim*), and the maximum number of evaluations (*MaxFEs*). If the number of times the fitness value is calculated in one iteration is *M*, the number of iterations (*T*) can be calculated as *T* = *MaxFEs*/*M*, which is determined by *MaxFEs* and the application time of the evaluation function. Therefore, the overall time complexity is *O*(SRWPSO) = *O*(*Sobol initialization*) + *O*(*Assessment and selection of initialization*) + *O*(*Random replacement strategy*) + *O*(*Adaptive weight strategy*). The complexity under the Sobol initialization is *O*(*N* × *dim*). The complexity under assessment and selection of initialization is *O*(*N* × *dim*). The complexity under the random replacement strategy is *O*(*N* × *dim* + 2 × *N* × *dim*). The complexity under the adaptive weight strategy is *O*(2 × 2 × *N* × *dim*). In conclusion, *O*(SRWPSO) = *O*(2 × *N* × *dim*) + *T* = (*O*(4 × *N* = *dim*) + *O*(3 × *N* × *dim*)) = *O*(*N* × *dim* + *T* × (7*N* × *dim*)).

## 5. The proposed bSRWPSO-FKNN

### 5.1. Binary conversion method

It is well known that feature selection is a binary-based discretization problem. However, the SRWPSO in this paper is proposed based on a continuous problem. Therefore, in order to make the SRWPSO applicable to the feature selection experiments, this subsection provides a binary conversion method suitable for the SRWPSO for converting from the continuous problem to the feature selection problem and finally proposes a novel discrete binary version of the SRWPSO, named bSRWPSO. The following is a partial description of the binary conversion process of the SRWPSO.

(1) Initialize the problem domain as [0,1]. In the problem, each dimension of each individual represents an attribute of the problem, and each feature has a data marker between 0 and 1.

(2) Discrete the continuous problem. As shown in Eq. 19, the obtained feature values are transformed into 0 or 1 by the V-transformation equation, indicating whether the feature is selected. Where 1 indicates that it is selected and 0 represents the opposite meaning.


(19) $$X_{d}\left(t+1\right)=\left\{ \begin{array}{c} X_{d},V\left(X_{d}\left(t\right)\right)\leq r\\ ∼X_{d},\mathrm{otherwise} \end{array}\right.$$

where *r* is a random number from 0 to 1, *X_d_* denotes the binary transformed position of the search agent, and *V*(⋅) is a V-shaped discretization equation, as shown in Eq. 20.


(20) V(x)=|tanh(x)|

### 5.2. Fuzzy K-nearest neighbor

K-nearest neighbors (KNN) (Cover and Hart, 1967; Jadhav et al., 2018; Tang et al., 2020) is a simple, efficient, nonparametric classification method proposed by Cover and Hart (1967) and one of the world-famous machine learning algorithms since the 20th century. In KNN, one of its classes is assigned according to the most common class in its K-nearest neighbors. Keller et al. (1985) combined fuzzy set theory with the KNN and proposed a fuzzy version of the KNN, named the FKNN (Keller et al., 1985; Chen et al., 2011, 2013; Mailagaha Kumbure et al., 2020). Unlike the individual classes of the KNN, the fuzzy affiliations of the samples of the FKNN are assigned to different classes according to Eq. 21.


(21) $$\mu_{i}\left(x\right)=\frac{\sum_{j=1}^{k}\mu_{i,j}\left(1/{∥{\text{x-x}}_{j}∥}^{2/\left(m-1\right)}\right)}{\sum_{j=1}^{k}\left(1/{∥{\text{x-x}}_{j}∥}^{2/\left(m-1\right)}\right)}$$

In the above equation, *i* = 1, 2, 3, …, *C* and *j* = 1, 2, 3, …, *k*. *C* denotes the number of classes and *k* represents the number of the nearest neighbors. In calculating the contribution of each neighbor to the affiliation value, the FKNN method determines the weight of the distance in the calculation process by using the fuzzy strength parameter *m*, which is usually taken as *m* ∈ (1, ∞). ∥ x-x_*j*_ ∥ is calculated using the Euclidean distance, which denotes the distance between x and its *j*-th nearest neighbor *x_j_*. μ_*i*,*j*_ is the membership degree of the pattern *x_j_* from the training set to the class *i*, among the *k* nearest neighbors of *x*.

### 5.3. Implementation of bSRWPSO-FKNN

This section proposes a novel feature prediction model, named bSRWPSO-FKNN, based on the binary SRWPSO and the FKNN, which provides technical support for conducting feature selection experiments. The principle is to optimize the subset of data produced during the experiment by using the ability of the bSRWPSO to find the global optimum in order to obtain a better and more suitable optimization set for feature selection experiments and then use the FKNN to perform feature prediction on the obtained optimization set. By the above method, we not only exploit the potential of the FKNN but also improve the efficiency and accuracy of the classification experiments.

In addition, to better achieve the classification performance of the bSRWPSO-FKNN, this paper provides an evaluation method based on error rate and feature subset for aiding feature prediction, as shown in Eq. 22.


(22) $$Fitness=\alpha \cdot Error+\beta \cdot \frac{R}{D}$$

where *Error* denotes the error rate of classification results, and the sum of classification accuracy is 1; *D* denotes the number of features in the dataset involved in feature selection; *R* denotes the number of features in the subset of data obtained by the feature selection experiment; α and β are two important weight parameters, and α + β = 1, and α = 0.99 reflects the importance of error rate.

**FIGURE 3:**
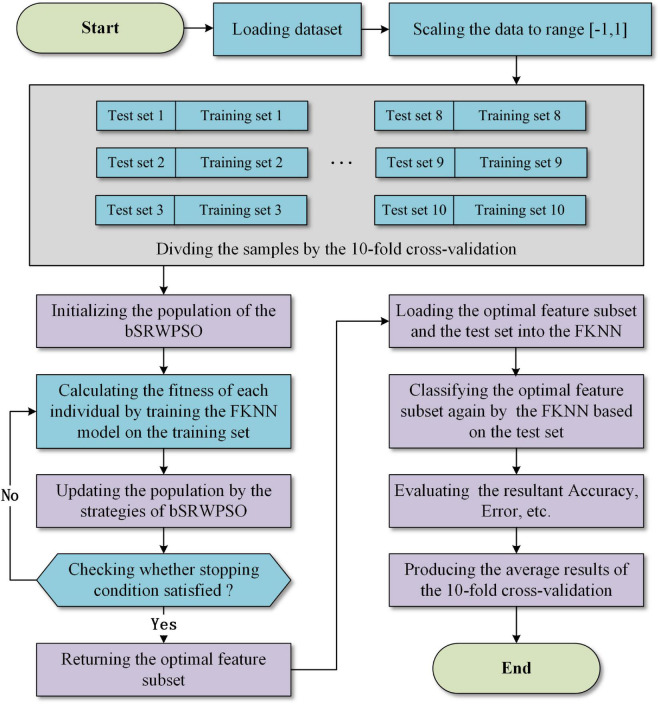
The basic workflow of the bSRWPSO-FKNNN.

In summary, the workflow of the bSRWPSO-FKNN proposed in this paper is shown in Figure 3.

## 6. Benchmark function validation

In this section, this paper experiments to test the performance of the SRWPSO based on 30 benchmark functions from the CEC 2014. The convergence process of the SRWPSO is analyzed from several aspects, and its ability to escape the local optimum and search for the global optimum is fully demonstrated.

### 6.1. Experimental setup

In order to verify the comprehensive ability of SRWPSO, this section sets up performance verification experiments for SRWPSO from four aspects, including mechanism combination verification experiments, quality analysis experiments, comparison experiments with traditional algorithms, comparison experiments with famous variants, and comparison experiments with new peer variants. At the same time, combined with the experimental results, this section analyzes the convergence process of SRWPSO and proves its excellent performance. As shown in Table 1, this subsection gives the specific details of the CEC 2014 benchmark function set. The parameters of the algorithms involved in this paper are shown in Table 2.

**TABLE 1:** Description of the 30 benchmark functions.

Class	No.	Functions	${\text{F}}_{i}^{*}={\text{F}}_{i}\left({\text{x}}^{*}\right)$
Unimodal functions	1	Rotated high conditioned elliptic function	100
	2	Rotated Bent Cigar function	200
	3	Rotated discus function	300
Simple multimodal functions	4	Shifted and rotated Rosenbrock’s function	400
	5	Shifted and rotated Ackley’s function	500
	6	Shifted and rotated Weierstrass function	600
	7	Shifted And rotated Griewank’s function	700
	8	Shifted Rastrigin’s function	800
	9	Shifted and rotated Rastrigin’s function	900
	10	Shifted Schwefel’s function	1,000
	11	Shifted and rotated Schwefel’s function	1,100
	12	Shifted and rotated Katsuura function	1,200
	13	Shifted and rotated HappyCat function	1,300
	14	Shifted and rotated HGBat function	1,400
	15	Shifted and rotated expanded Griewank’s plus Rosenbrock’s function	1,500
	16	Shifted and rotated expanded Scaffer’s F6 function	1,600
Hybrid functions	17	Hybrid function 1 (*N* = 3)	1,700
	18	Hybrid function 2 (*N* = 3)	1,800
	19	Hybrid function 3 (*N* = 4)	1,900
	20	Hybrid function 4 (*N* = 4)	2,000
	21	Hybrid function 5 (*N* = 5)	2,100
	22	Hybrid function 6 (*N* = 5)	2,200
Composition functions	23	Composition function 1 (*N* = 5)	2,300
	24	Composition function 2 (*N* = 3)	2,400
	25	Composition function 3 (*N* = 3)	2,500
	26	Composition function 4 (*N* = 5)	2,600
	27	Composition function 5 (*N* = 5)	2,700
	28	Composition function 6 (*N* = 5)	2,800
	29	Composition function 7 (*N* = 3)	2,900
	30	Composition function 8 (*N* = 3)	3,000

**TABLE 2:** The parameters of the algorithms involved in this article.

Algorithms	Parameters
SRWPSO	*V*_*max*_ = 6;*c*_1_ = 2; *c*_2_ = 2; *s* = 0.01;*w* = [1, 0]
PSO	*V*_*max*_ = 6; *c*_1_ = 2; *c*_2_ = 2;
ACOR	*k* = 10; *q* = 0.5; *ξ* = 1
DE	*Scaling factor* = 0.5; *crossover probability* = 0.5
SCA	*r*1 = [2, 0]
HHO	*Rabbit Energy* = [2, 0]
GWO	*a* = [2, 0]
WOA	*a*_1_ = [2, 0]; *a*_2_ = [−2, −1]; *b* = 1
BA	*Qmin* = 0; *Qmax* = 2
MFO	*a* = 2; *b* = 1
WDO	–
SCADE	β_*min*_ = 0.2; β_*max*_ = 0.8; *P*_*cr*_ = 0.8 *or P*_*cr*_ = 0.1
CBA	*Qmin* = 0; *Qmax* = 2
RCACO	*ξ* = 0.9; *k* = 10; *q* = 0.8; *P*_1_ ∈ 0, 1 *and P*_1_0.25, 0.5, 0.75, 1; μ = 4
m_SCA	*JR* = 0.1; *a* = 2; *r*_1_ = [2, 0];
CLACO	*ξ* = 1; *k* = 10; *q* = 0.5; β = 1.5
SCA_PSO	*M* = 4; *N* = 9; *c*_1_ = 2; *c*_2_ = 2; *a* = 2
RDWOA	*a*_1_ = [2, 0]; *a*_2_ = [−2, −1]; *b* = 1; *s* = 0
OBLGWO	*a*_1_ = [2, 0]; *a*_2_ = [−2, −1]; *b* = 1; *beta* = [2, 0]
FSTPSO	*c*_1_ = 2; *c*_2_ = 2; *w* = 0.9
EWOA	*wMax* = 0.7; *wMin* = 0.2; *a* = [2, 0];
EESHHO	*Rabbit Energy* = [2, 0]
XMACOR	ξ = 1; *k* = 10; *q* = 0.5; β = 1.5
CAGWO	*type* = 2; *a* = [2, 0]
SGLSCA	*a* = 2; *k* = 1
IGWO	*r*_1_ = *r*_2_ = *rand*(0, 1); β = ω = 10; *C* = 2 = *r*_2_
GCHHO	*Rabbit Energy* = [2, 0]

For the purpose of increasing the persuasion of test outcomes, we utilized two representative statistical standards in the analysis, namely average value (AVG) and variance (STD). In the analysis part, the AVG is employed to represent the comprehensive capability of the algorithm, and the smaller its value is, the better the comprehensive performance of the algorithm is; the STD reflects the performance state of the algorithm, and the smaller its value is, the more stable its comprehensive performance is. Then, to further discuss the comparative experimental results, this section provides two popular statistical methods for the experimental analysis process: the Wilcoxon signed-rank test (García et al., 2010) and the Friedman test (García et al., 2010). The “+,” “=,” and “–” appearing in the Wilcoxon signed-rank test, respectively mean that the performance of SRWPSO is superior to, equal to and inferior to competitors. In the table, the optimal data of the experimental results are highlighted in black. Eventually, some of the convergence curves are drawn to visualize the convergence effect of the algorithms.

In addition, in order to balance the influence of the experimental process on the experimental outcomes, the experimental environment was unified from the internal and external aspects of the experiment. As displayed in Table 3, the study sets the population size, test times, target dimension, and other aspects of each algorithm during the experiment to eliminate the influence of internal experimental parameters on the performance of each algorithm. The difference is that the maximum number of the evaluation is used in this experiment instead of the number of iterations, which can be calculated by using iteration times. As shown in Table 4, the study uses the same experimental equipment to avoid the interference of the external experimental environment, thus further increasing the fairness and scientific nature.

**TABLE 3:** The parameter setting of the experiment.

Parameters	Parameter meaning	Value
*N*	The population size	30
*Fold*	Random tests number	30
*Dim*	Objective function dimensions	30
*Ub*	The upper boundary of the search space	100
*Lb*	The lower boundary of the search space	−100
*MaxFEs*	Maximum evaluations number	300,000

**TABLE 4:** Description of the experimental environment.

Equipment	Parameters
System version	Windows 11 Professional Edition
System type	64-bit operating systems, x64-based processors
Processor	11th Gen Intel(R) Core (TM) i7-11700 @ 2.50GHz 2.50 GHz
RAM	32.0 GB
Matlab version	Matlab2021b

### 6.2. Impacts of components

In this part, the experimental process of SRWPSO is presented. In this process, this paper explores the impact of three improved strategies on the performance of PSO based on the CEC 2014 benchmark function set. Table 5 shows the different combinations in the improvement process. In the table, the SOB represents the Sobol initialization strategy, the RRS represents the random replacement strategy, and the AWS represents the adaptive weight strategy.

**TABLE 5:** The results of strategy combinations.

	SOB	RRS	AWS
SRWPSO	1	1	1
SRPSO	1	1	0
SWPSO	1	0	1
RWPSO	0	1	1
SOBPSO	1	0	0
RRPSO	0	1	0
AWPSO	0	0	1
PSO	–	–	–

Supplementary Appendix Table 1 reflects the effects of the different combinations of strategies on the comprehensive performance of PSO through AVG and STD. By analyzing the data in the table, it can be seen that the SRWPSO occupies the largest share of the number of excellent performances among the 30 test functions, especially in the unimodal functions and composition functions. For AVG, the smaller value obtained by the SRWPSO indicates that it performs better on the problem. Of course, the more frequency of occurrences of the minimum state of the AVG in the 30 test functions means that the SRWPSO is more adaptable to different problems. For STD, a smaller value obtained by the SRWPSO indicates a more stable performance on the corresponding problem. Similarly, the number of minimum states of the STD also reflects the adaptability of the corresponding algorithm to different problems in a certain extent. In addition, the performance of the PSO under single-strategy action is not outstanding enough compared to the traditional PSO, and even slightly worse than the traditional PSO in some problems. In the dual-strategy role, the PSO performs relatively well in terms of overall capability, especially the SWPSO and the RWPSO. Of course, by observing the table, it is easy to find that the SRWPSO shows the best-combined ability in this comparison test under the role of three strategies.

Supplementary Appendix Table 2 presents the *p*-values acquired based on the Wilcoxon signed-rank test. In analyzing the experiments, the article has bolded the experimental results less than 0.05 in the table, indicating that the excellent ability of the SRWPSO has statistical significance and higher confidence relative to the algorithms participating in the comparison. The table shows that the *p*-values less than 0.05 occupy a significant proportion compared to those greater than 0.05, especially relative to the performance of the traditional PSO on the 30 benchmark functions. It indicates that the SRWPSO proposed in this paper outperforms the single-strategy improvement variant, the two-strategy improvement variant, and the original PSO in the comparison experiments.

To enhance the persuasiveness of the experimental results, the experimental results based on the Wilcoxon signed-rank test are given in Table 6. By analyzing the table, it is easy to find that the SRWPSO shows the best comprehensive performance in this experiment, and the average value of the Wilcoxon signed-rank test obtained by it is much smaller than that of the second-ranked SWPSO. In addition, the SRWPSO performs better than the SWPSO on 15 of the 30 benchmark problems, and 14 have similar optimization capabilities. Of course, compared to the traditional PSO, the SRWPSO is more outstanding, with 26 benchmark functions performing well, and only one benchmark function performing less well than the traditional PSO, except for three with equal performance.

**TABLE 6:** The results of Wilcoxon signed-rank test of different versions.

Algorithms	+/−/=	Mean	Rank
**SRWPSO**	**–**	**1.90**	**1**
SRPSO	17/1/12	4.07	4
SWPSO	15/1/14	3.33	2
RWPSO	11/1/18	3.47	3
SOBPSO	21/0/9	5.37	6
RRPSO	25/1/4	5.37	6
AWPSO	23/2/5	4.70	5
PSO	26/1/3	6.40	8

Bold values represent the optimal data.

**FIGURE 4:**
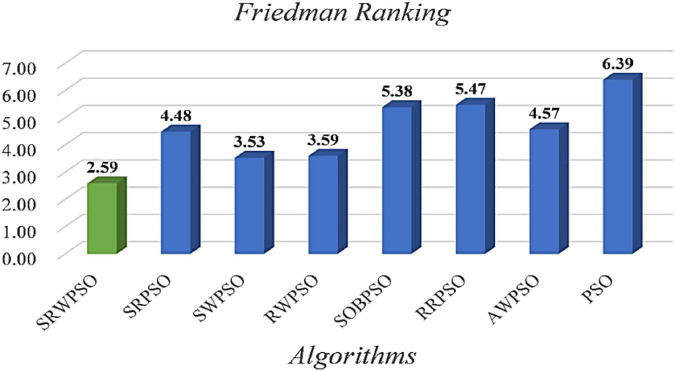
The results of the Friedman test of different versions.

In order to advance to increase the authority of the experimental results, the statistical results based on the Friedman test are given in Figure 4. As seen from the figure, the Friedman statistic value obtained by the SRWPSO is 2.59, which is the smallest among the comparison results. In addition, it is not only much smaller than the traditional PSO, which ranks at the bottom, but also smaller than the SWPSO, which ranks second. This again indicates that the comprehensive performance of the SRWPSO performs relatively best in this experiment and also provides the basis for the SRWPSO proposed in this paper.

### 6.3. The qualitative analysis of SRWPSO

**FIGURE 5:**
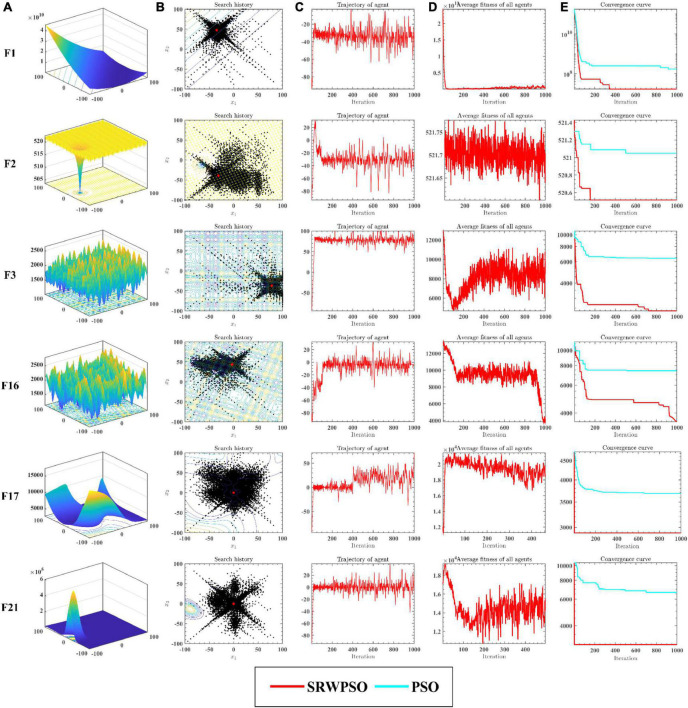
The analysis results of SRWPSO and PSO from multiple perspectives. See section 6.3 for details.

Figure 5 analyzes the performance of the SRWPSO from several perspectives. Figure 5A provides a three-dimensional view of the benchmark function. Figure 5B marks the two-dimensional distribution of the historical search positions of the SRWPSO during the search for superiority, where the red markers indicate the best positions throughout the search process and the black dots indicate the historical search positions. Figure 5C shows the change of the first dimension of each position during the iteration. Figure 5D gives the variation of the average fitness of all individuals in the population during the iteration. Figure 5E then provides the convergence curves of the SRWPSO and the PSO. The three-dimensional and two-dimensional distributions show that the SRWPSO is able to obtain better global optimal solutions on benchmark functions of different complexity. The variation of the first dimension at each position shows that the amplitude of oscillation at the beginning of the iteration is small. As the number of iterations keeps increasing, the amplitude of oscillations increases and stabilizes to a certain extent, indicating that individuals can better traverse the search space and increase the diversity of the population, thus enhancing the ability to escape the local optimal position. Similarly, it can be seen from Figure 5D that the average fitness values of the SRWPSO have a large oscillation amplitude on F12, F16, F17, and F21, again indicating the existence of population diversity during the search process. the convergence curves of the SRWPSO and the PSO show that the final convergence accuracy of the SRWPSO is better than that of the PSO. The convergence curves in Figure 5E also shows that the convergence ability of the SRWPSO is much larger than that of the PSO; the convergence curves on F1, F2, and F16 show that the SRWPSO has a solid ability to escape from the local optimum. Each inflection point on the convergence curve represents a successful escape from the local optimum position.

**FIGURE 6:**
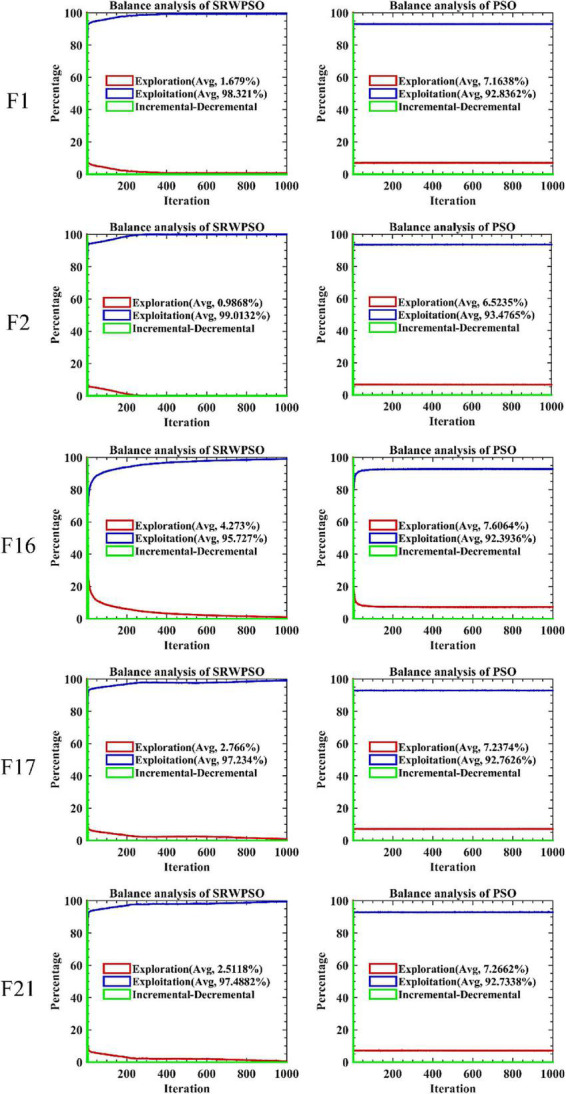
The balance analysis results of SRWPSO and PSO.

The results of the equilibrium analysis of the corresponding benchmark functions in Figure 5 are given in Figure 6. By comparing the equilibrium images of the SRWPSO and the PSO, it is easy to observe that there is a significant improvement in the development capability of the SRWPSO relative to the PSO, which makes the SRWPSO based on the three optimization strategies reach a better balance point in both exploration and development stages, thus making the convergence speed and final convergence accuracy of the SRWPSO better than the PSO.

### 6.4. Comparison with traditional algorithms

This subsection discusses the experimental results of comparing the SRWPSO with nine well-known traditional algorithms to demonstrate the core advantages of the SRWPSO further. In this comparison, the traditional algorithms involved include ant colony optimization based on continuous optimization (ACOR) (Dorigo, 1992; Dorigo and Caro, 1999; Socha and Dorigo, 2008), different evolution (DE) (Storn and Price, 1997), sine cosine algorithm (SCA) (Mirjalili, 2016), HHO (Heidari et al., 2019b), grey wolf optimization (GWO) (Mirjalili et al., 2014), whale optimization algorithm (WOA) (Mirjalili and Lewis, 2016), bat-inspired algorithm (BA) (Yang, 2010), moth-flame optimization (MFO) (Mirjalili, 2015), and wind-driven optimization (WDO)(Bayraktar et al., 2010).

Supplementary Appendix Table 3 gives the results of the SRWPSO compared with nine traditional algorithms based on AVG and STD. In terms of the number of the best solutions obtained on the 30 benchmark functions, the SRWPSO ranks first in this experiment. This indicates that the SRWPSO not only has the best comprehensive performance relatively but also its adaptability to different problems. In the same way, it’s evident that the SRWPSO still has a tremendous advantage over the other nine algorithms in finding the global optimum.

The analysis of the Wilcoxon signed-rank test in Table 7 shows that the SRWPSO ranks first in this comparison experiment with an overall mean of 1.53. It is 1.67, smaller than the average score of DE, which is ranked second overall and outperforms DE on 20 functions. The results of the *p*-values obtained during the Wilcoxon signed-rank test are presented in Supplementary Appendix Table 4. The data bolded in black in the table indicate less than 0.05, indicating that the experimental results are credible. The table shows that the *p*-values are essentially less than 0.05 in all the comparison results, indicating that the optimal solutions obtained by SRWPSO are credible when compared with the other nine conventional algorithms.

**TABLE 7:** The results of the Wilcoxon signed-rank test of SRWPSO with traditional algorithms.

Algorithms	+/−/=	Mean	Rank
**SRWPSO**	**–**	**1.53**	**1**
ACOR	22/2/6	4.97	4
DE	20/6/4	3.20	2
SCA	30/0/0	8.70	10
HHO	22/0/8	4.47	3
GWO	26/3/1	5.73	5
WOA	28/0/2	6.83	8
BA	29/1/0	5.97	7
MFO	30/0/0	7.50	9
WDO	29/0/1	5.90	6

Bold values represent the optimal data.

**FIGURE 7:**
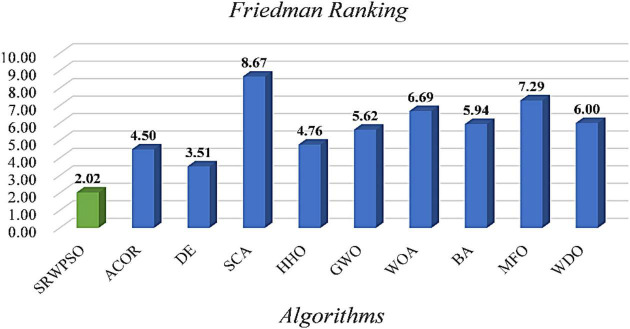
The results of Friedman test of SRWPSO with traditional algorithms.

**FIGURE 8:**
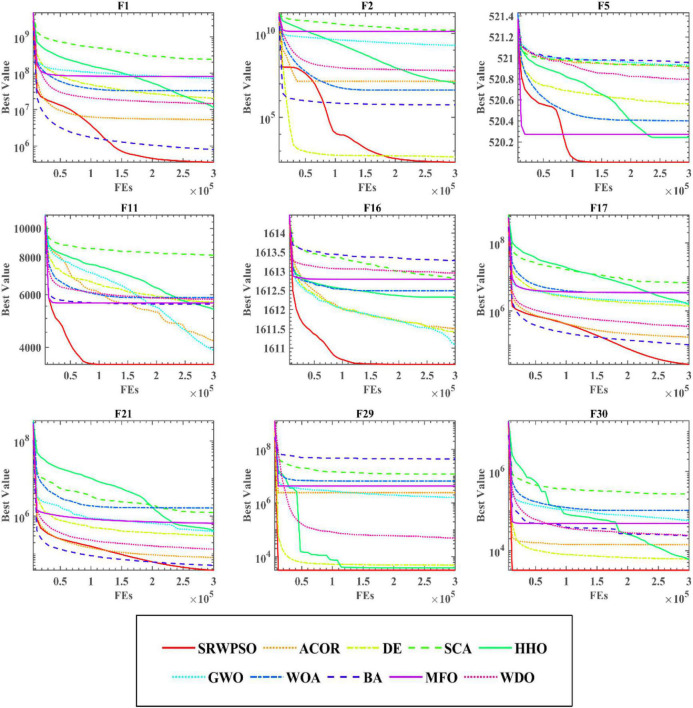
The convergence curves of SRWPSO with traditional algorithms.

To further demonstrate the performance of SRWPSO, Figure 7 analyzes the experimental results based on the Friedman test. It is not obvious from the figure that the SRWPSO ranks first, obtaining the Friedman test result of 2.02, and DE ranks second with a score of 3.48 in the experiment. Thus, this is another evidence that SRWPSO still has a clear advantage compared to the basic algorithm. Figure 8 shows the representative partial convergence curves of SRWPSO compared with the other nine traditional algorithms. Among them, SRWPSO has significantly better convergence accuracy than the other algorithms. In addition, SRWPSO performs significantly better in terms of convergence speed on F11, F16, F29, and F30. On F1, F2, and F3, the convergence curves of SRWPSO have obvious inflection points compared with other algorithms, which indicates that SRWPSO has a more vital ability to escape from the local optimum position on this type of problem. Overall, SRWPSO is more competitive than the other nine traditional algorithms in searching for the global optimum. Therefore, when SRWPSO is compared with other basic algorithms, its core advantages are also well demonstrated.

**FIGURE 9:**
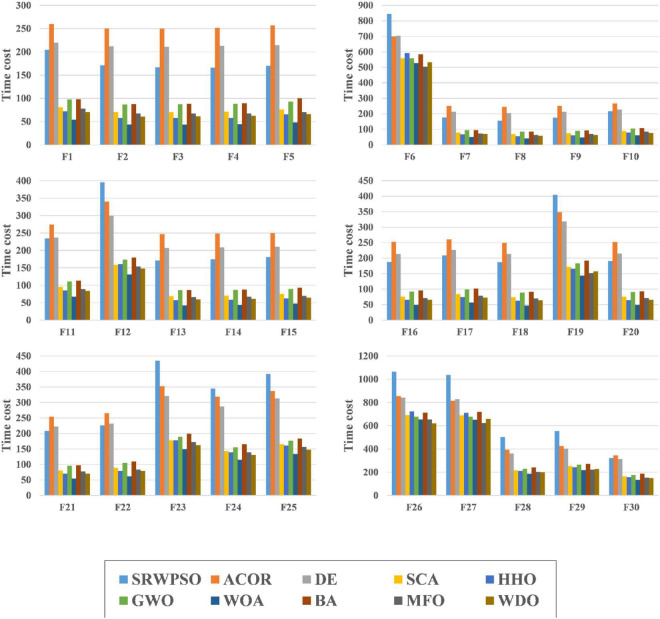
The time complexity evaluation results of SRWPSO with traditional algorithms.

Figure 9 shows the time cost consumed by all algorithms in this experiment when run on the 30 benchmark functions. Each color in the figure represents an algorithm, and the experimental results are calculated in seconds. It is easy to see that SRWPSO has a higher consumption in the optimization task relative to these original classical algorithms. It is also easy to understand that this situation occurs due to the introduction of several improvement strategies in SRWPSO. However, the difference compared to ACOR and DE is not very large and even less consuming than them for most functions. This indicates that the computational cost of SRWPSO has an advantage over some well-known original algorithms.

### 6.5. Comparison with famous variants

To further verify that the comprehensive performance of SRWPSO has core advantages, this subsection compares SRWPSO with nine well-known variants of algorithms proposed in recent years, mainly hybridizing SCA with DE (SCADE) (Nenavath and Jatoth, 2018), chaotic BA (CBA) (Adarsh et al., 2016), chaotic random spare ACO (RCACO) (Dorigo, 1992; Dorigo and Caro, 1999; Zhao et al., 2021), modified SCA (m_SCA) (Qu et al., 2018), ACO with Cauchy and greedy levy mutations (CLACO) (Dorigo, 1992; Dorigo and Caro, 1999; Liu L. et al., 2021), hybridizing SCA with PSO (SCA_PSO) (Nenavath et al., 2018), double adaptive random spare reinforced WOA (RDWOA) (Chen et al., 2019), boosted GWO (OBLGWO) (Heidari et al., 2019a), and fuzzy self-tuning PSO (FSTPSO) (Nobile et al., 2018).

Supplementary Appendix Table 5 analyzes the AVG and STD obtained in the experiment after 30 independent runs. It can be seen that SRWPSO obtains the largest number of minimum AVG, which indicates that its convergence performance and adaptability to the problem are more advantageous than the other nine well-known variants in this comparison experiment. Also, SRWPSO obtains the largest number of minimum STD, which indicates that it exhibits performance with more stability.

The analytical results of the Wilcoxon signed-rank test are given in Table 8. As seen from the table, SRWPSO achieves relatively better global optimal solutions for most of the functions and ranks first in this experiment with an overall mean of 1.87. In addition, it is not difficult to observe the second column of the table to find that SRWPSO outperforms the second-ranked CLRCO by 17 out of 30 benchmark functions, 19 outperforms the third-ranked RCACO, and even 30 outperforms the bottom-ranked FSTPSO. This indicates that the comprehensive performance of SRWPSO has a powerful core advantage among all the algorithms participating in this experiment. To further demonstrate the core advantage of SRWPSO, Supplementary Appendix Table 6 analyzes the *p*-values obtained in the Wilcoxon signed-rank test. The bolded and blackened data in the table indicate that the *p*-values obtained are less than 0.05, again indicating that it is plausible that SRWPSO excels over the other nine well-known variants for the corresponding problem. Thus, it is credible that we can easily see that SRWPSO has superior performance in most comparisons through the table.

**TABLE 8:** The results of the Wilcoxon signed-rank test of SRWPSO with famous variants.

Algorithms	+/−/=	Mean	Rank
**SRWPSO**	**–**	**1.87**	**1**
SCADE	28/0/2	8.40	9
CBA	28/0/2	6.60	7
RCACO	19/8/3	3.50	3
m_SCA	28/1/1	6.70	8
CLACO	17/9/4	3.23	2
SCA_PSO	23/2/5	4.67	5
RDWOA	20/2/8	4.43	4
OBLGWO	25/0/5	5.63	6
FSTPSO	30/0/0	9.20	10

Bold values represent the optimal data.

**FIGURE 10:**
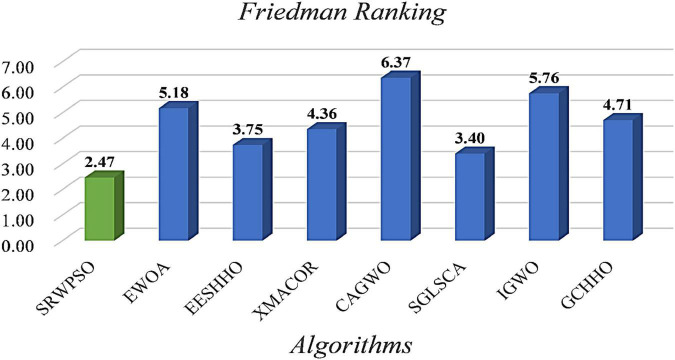
The results of Friedman test of SRWPSO with famous variants.

**FIGURE 11:**
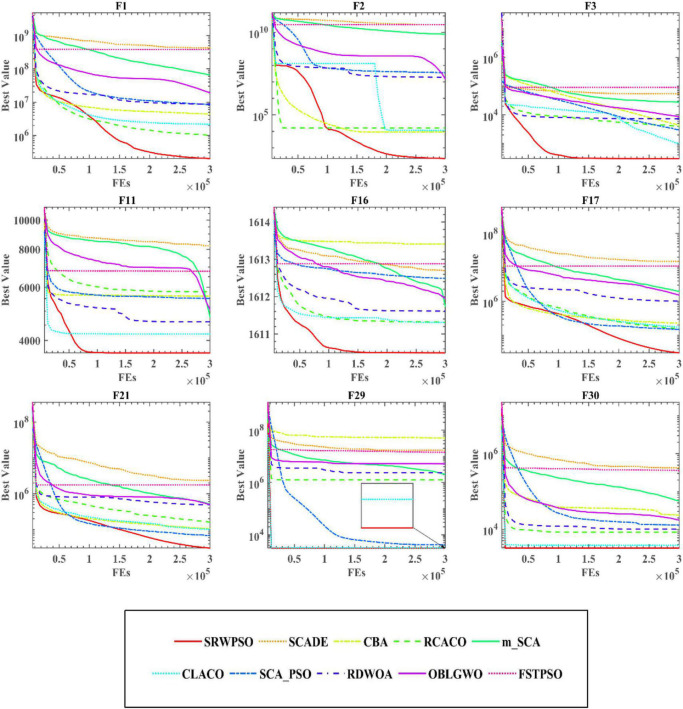
The convergence curves of SRWPSO with famous variants.

The results of the Friedman test given in Figure 10 show that SRWPSO ranks first with 2.37 and CLACO ranks second with 3.34, which proves that SRWPSO outperforms the other nine methods. To further analyze the convergence capability of SRWPSO, we give some of the convergence curves in this comparison experiment in Figure 11. From the figure, it can be observed that SRWPSO has the best convergence accuracy on the listed benchmark functions. In terms of convergence speed, SRWPSO is relatively more excellent on the F2, F3, F11, F16, and F30, while it is well demonstrated to have the ability to continuously find the global optimum on F1, F2, F17, and F21. Thus, the above analysis strongly demonstrates that the comprehensive performance of SRWPSO still has significant advantages compared with other advanced variants.

**FIGURE 12:**
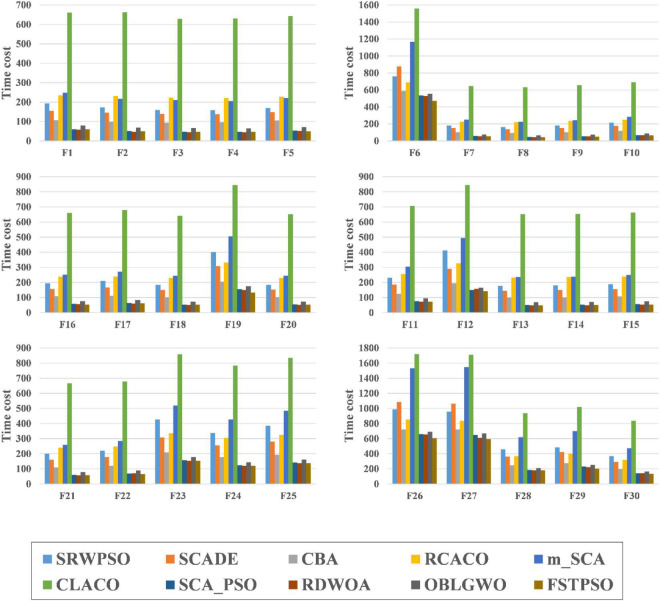
The time complexity evaluation results of SRWPSO with famous variants.

Figure 12 shows the time cost consumed by SRWPSO with 9 other famous variants for 30 optimization tasks. Each color in the figure represents an algorithm, and the experimental results are in seconds. SRWPSO consumes less than m_SCA and CLACO in all 30 optimization tasks, with the most prominent advantage over CLACO in particular. In addition, it is not difficult to find that SRWPSO consumes less than RCACO in most optimization tasks upon closer inspection. The difference compared to SCADE and CBA is also not very large. Of course, SRWPSO also has some weaknesses against several other variants, which are caused by introducing optimization strategies with different complexity to algorithms with different complexity. In conclusion, SRWPSO has good computational efficiency in comparison with famous variants.

### 6.6. Comparison with new peer variants

To highlight the core strengths of SRWPSO, we make a comparison of SRWPSO with seven new peer variants in this section. These variants are mainly enhanced WOA (EWOA) (Tu et al., 2021a), elite evolutionary strategy-based HHO (EESHHO) (Li C. et al., 2021), ACOR based on the directional crossover (DX) and directional variant (DM) (XMACOR) (Qi et al., 2022b), cellular grey wolf optimizer with a topological structure (CAGWO) (Lu et al., 2018), multi-core SCA (SGLSCA) (Zhou W. et al., 2021), improved GWO (IGWO) (Cai et al., 2019), and HHO based on Gaussian mutation and cuckoo search (GCHHO) (Song et al., 2021).

Supplementary Appendix Table 7 compares SRWPSO with new peer variants. It is obvious that SRWPSO has the best performance on unimodal functions (F1, F2, and F3) and composition functions (F23–F30). In addition, the AVG and STD of SRWPSO obtain the highest number of optimal in this experiment, which indicates that the method is relatively the most adaptable to different problems. In another way, it means that SRWPSO has not only better optimization ability but also better stability. Supplementary Appendix Table 8 shows the *p*-value of the comparison result of SRWPSO with new peer variants. The data marked in black in the table indicate that the *p* value is greater than 0.05, which indicates that these data lack statistical significance. On the contrary, other data have statistical significance and can be powerful evidence to verify SRWPSO. It is clear that most of the data is less than 0.05, which indicates that SRWPSO is better than the other algorithms in terms of the corresponding functions.

Table 9 shows the results of the Wilcoxon signed-rank test of SRWPSO with new peer variants. It is clear that SRWPSO ranks first in this test with a mean score of 1.90, which is 0.9, smaller than the second-ranked SGLSCA. Specifically, SRWPSO is stronger than SGLSCA on 13 functions, equal on 14 functions, and worse on only 3 functions. In addition, SRWPSO is superior to EWOA, XMACOR, CAGWO, IGWO, and GCHHO on 22 or more functions. In conclusion, this test shows that SRWPSO still has a significant advantage in comparison with the new peer variants.

**TABLE 9:** The results of the Wilcoxon signed-rank test of SRWPSO with new peer variants.

Algorithms	+/−/=	Mean	Rank
**SRWPSO**	**–**	**1.90**	**1**
EWOA	26/3/1	5.50	6
EESHHO	18/3/9	3.50	3
XMACOR	23/6/1	4.33	4
CAGWO	27/1/2	6.33	8
SGLSCA	13/3/14	2.80	2
IGWO	26/1/3	5.87	7
GCHHO	22/0/8	4.73	5

Bold values represent the optimal data.

**FIGURE 13:**
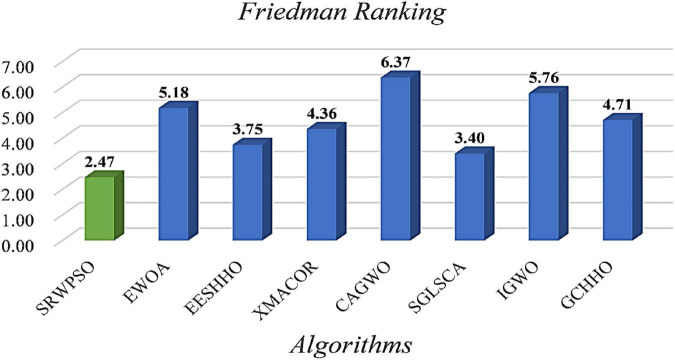
The results of Friedman test of SRWPSO with new peer variants.

**FIGURE 14:**
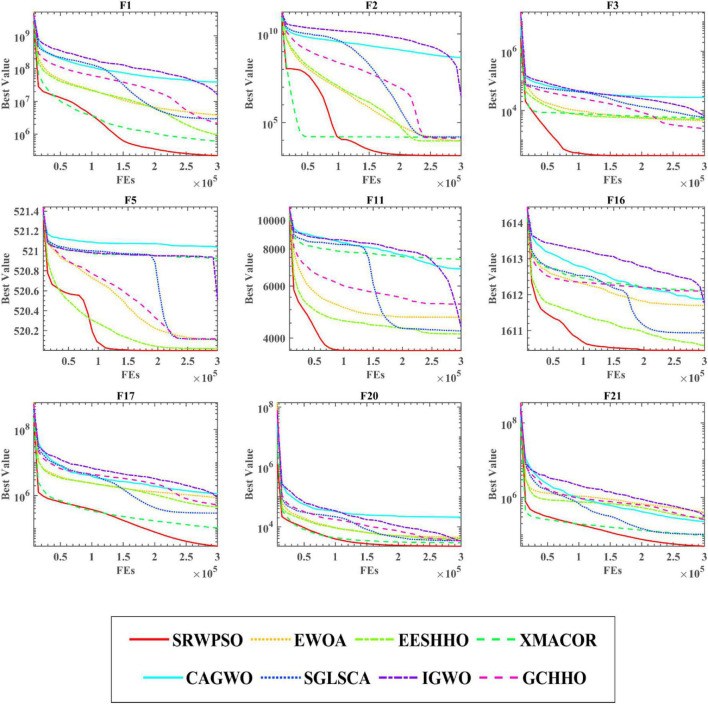
The convergence curves of SRWPSO and new peer variants.

Figure 13 is the results of the Friedman test of SRWPSO with new peer variants. It is not difficult to see from the figure that SRWPSO obtains the best scores in the Friedman test, which is 0.93 smaller than the second-ranked SGLSCA and 3.9 smaller than the last ranked CAGWO. Figure 14 shows 9 convergence images of SRWPSO and new peer variants. In this figure, SRWPSO obtains the best convergence accuracy in all 9 convergence images. Also, the convergence curves of SRWPSO on F1, F2, F3, F5, and F16 have obvious inflection points compared to the other variants, which indicates that the algorithm has a stronger ability to escape from local optimal on the corresponding functions. Moreover, it is obvious that SRWPSO also has better convergence speed. In conclusion, SRWPSO has a very significant core advantage in comparison with the new peer variant.

**FIGURE 15:**
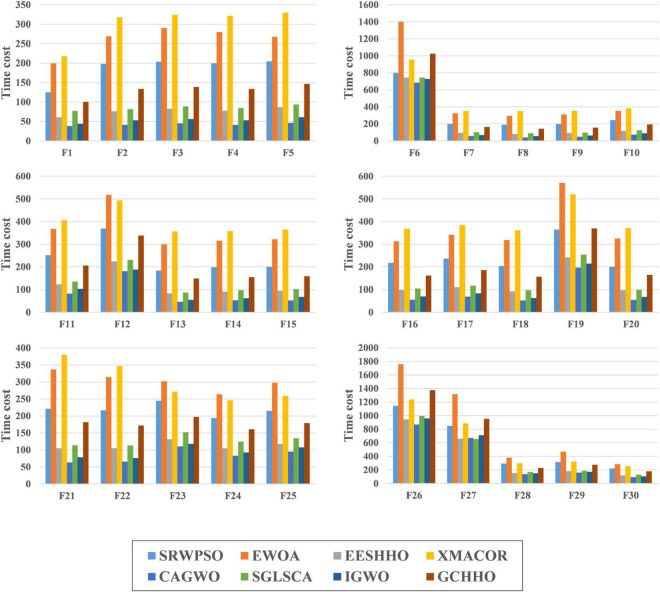
The time complexity evaluation results of SRWPSO with new peer variants.

Figure 15 is the time complexity evaluation results of SRWPSO with new peer variants in this experiment. In this figure, each color represents an algorithm, and the experimental results are in seconds. SRWPSO consumes significantly less than EWOA and XMACOR on all 30 functions. Except for F1, F2, and F3, there is not much difference between them, although SRWPSO is more time-consuming than GCHHO. It is not promising that SRWPSO is more time-consuming relative to EESHHO, SGLSCA, and IGWO. It is not difficult to understand this situation, mainly caused by the introduction of optimization strategies with different degrees of complexity in algorithms with different degrees of complexity. In conclusion, SRWPSO has better computational efficiency than the new peer variants. The SRWPSO and the future improved PSO can be applied in different fields, such as human activity recognition (Qiu et al., 2022), dynamic module detection (Ma et al., 2020; Li D. et al., 2021), recommender system (Li et al., 2014, 2017), smart contract vulnerability detection (Zhang L. et al., 2022), privacy protection of electronic medical records (Wu et al., 2022), named entity recognition (Yang Z. et al., 2022), structured sparsity optimization (Zhang X. et al., 2022), microgrids planning (Cao et al., 2021c), location-based services (Wu et al., 2020; Wu Z. et al., 2021), disease prediction (Su et al., 2019; Li L. et al., 2021), medical data processing (Guo et al., 2022), drug discovery (Zhu et al., 2018; Li Y. et al., 2020), and object tracking (Zhang et al., 2015).

## 7. Feature selection experiments and analysis

In this section, the performance of the bSRWPSO-FKNN is first tested and validated on the basis of nine public datasets in the UCI. Then, this section performs a secondary performance validation of the suggested model based on a medical dataset and successfully extracts the key features affecting the incidence of AD through 10 times 10-fold cross-validation experiments combined with clinical medical practice.

### 7.1. Experimental setup

For the purpose of verifying that the proposed bSRWPSO-FKNN has better performance in feature selection, a series of comparative tests are conducted in this paper between bSRWPSO and some well-known algorithms in this field based on nine public datasets and one medical dataset. The details of the public datasets are described in Table 10. The main binary swarm intelligence algorithms involved in the tests are bSCGWO, bGWO, bGSA, bPSO, bALO, bBA, bSSA, bQGWO, bHHO, and bSMA. In addition to converting the swarm intelligence methods involved in the comparison to a binary discrete version suitable for feature selection, the parameters unique to the algorithms themselves remain unchanged, as shown in Table 11.

**TABLE 10:** A detailed description of the public dataset.

Datasets	Samples	Features	Classes
Australian	690	15	2
Breast	569	31	2
Clean	476	167	2
Heart	270	14	2
JPNdata	152	11	2
SpectEW	267	23	2
Vote	101	17	2
wdbc	569	31	2
Wielaw	240	31	2

**TABLE 11:** Key parameters of the experiment.

Public parameters	Methods	Other parameters
Iteration = 50 *N* = 20 Fold = 10	bSRWPSO	w = [1 0]
	bSCGWO	*q* = 2; *walk* = 1
	bGWO	*a* = [2 0]
	bGSA	*wMax* = 20; *wMin* = 1*e* − 10
	bPSO	*Max = 0.9, Min = 0,4*
	bALO	–
	bBA	*a = 0.5; r = 0.5*
	bSSA	*c*_1_ = random in [0, 1]; *c*_2_ = random in [0, 1]; β = 1.5
	bQGWO	a = [4 0]
	bHHO	ω_*a*_ = 0.7; ω_*b*_ = 0.2; | *E* | = [2 0]; β = 1.5
	bSMA	*a* = [0,5]; *b* = [0,1]

To facilitate the verification of the core advantages of the bSRWPSO-FKNN, four evaluation criteria, including Accuracy, Sensitivity, MCC, and F-measure, are applied in this paper to evaluate the methods involved in the comparison experiments, and the average value (AVG) and variance (STD) obtained during the experiments were analyzed and compared. Here, unlike the benchmark function validation part, a larger AVG indicates a more robust average performance of the method. In addition, the optimal data of the experimental results are highlighted in black. The details of the evaluation criteria are described in Table 12.

**TABLE 12:** Detailed description of the evaluation criteria.

Name	Formula	Remark
Accuracy	$Accuracy=\frac{TP+TN}{TP+FP+FN+TN}$	A higher accuracy rate indicates a larger proportion of correctly predicted samples
Sensitivity	$\text{Sensitivity}=\frac{TP}{TP+FN}$	The closer the sensitivity to 1, the better the performance of the binary classification method to correctly identify instances
MCC	$MCC=\frac{TP\times TN-FP\times FN}{\sqrt{\left(TP+FP\right)\times \left(TP+FN\right)\times \left(TN+FP\right)\times \left(TN+FN\right)}}$	A closer MCC to 1 indicates a more perfect prediction of the subject
F-measure	$F-measure=\frac{TP}{TP+\frac{FN+FP}{2}}$	A higher F-measure indicates that the classification results are more following expectations

In the table above, true positive (TP) and true negative (TN) are correct situations, which means that positive and negative classes are correctly predicted, respectively. At the same time, false positive (FP) and false negative (FN) are error cases. The former means that negative class is wrongly predicted as positive class, while the latter indicates that positive class is incorrectly predicted as negative class.

In addition, this paper also evaluates the comparison models that participated in the feature selection experiments by the Friedman test and then gave the Friedman ranking corresponding to the comparison models, thus demonstrating more intuitively that the bSRWPSO-FKNN has relatively better feature selection performance. We conducted all of our experiments using fair aspects, which are recognized as being common across a wide range of computer platforms, while adhering to the guidelines for unbiased comparisons in preceding AI-based work (Duan et al., 2022; Jin et al., 2022; Yang B. et al., 2022). If the professional designed the programming, it is assumed that these variables occur regardless no matter how the approach is used (Li H. et al., 2021; Zhang Z. et al., 2022; Lu et al., 2023). Finally, to ensure the fairness of the experimental process, the internal environment of all experiments is kept consistent, and the external experimental environment is kept consistent with the experimental part of the benchmark function.

### 7.2. Public dataset experiments

The evaluation results of the classification accuracy of bSRWPSO with the other ten binary algorithms are given in Table 13. From the table, it can be seen that bSRWPSO has the largest average classification accuracy on all nine public datasets tested, indicating that the method ranks first on the corresponding datasets. Secondly, it can also be seen that the stability of bSRWPSO is also the strongest among the compared methods. Table 14 shows the average ranking results of the 11 comparison methods based on nine public datasets obtained by the Friedman test for this experiment. As shown in the second column of the table, the mean value of the Friedman test of bSRWPSO based on nine public datasets is 1.22, which is the smallest among all comparison algorithms, indicating that bSRWPSO is ranked first in this classification accuracy test.

**TABLE 13:** The analysis results of accuracy.

	Australian		BreastEW		Clean	
	AVG	STD	AVG	STD	AVG	STD
bSRWPSO	**9.24630E-01**	2.53900E-02	**9.94740E-01**	**8.47750E-03**	**9.97920E-01**	**6.58810E-03**
bSCGWO	9.12930E-01	3.16970E-02	9.89380E-01	1.24270E-02	9.97870E-01	6.72830E-03
bGWO	9.13000E-01	**2.18680E-02**	9.91290E-01	1.22650E-02	9.95830E-01	8.78410E-03
bGSA	9.20340E-01	2.59130E-02	9.92980E-01	1.22670E-02	9.89450E-01	1.79090E-02
bPSO	9.13120E-01	2.22600E-02	9.89470E-01	1.22680E-02	9.74730E-01	2.16530E-02
bALO	9.10140E-01	3.11640E-02	9.93010E-01	1.22380E-02	9.76860E-01	1.54750E-02
bBA	7.94440E-01	7.52480E-02	9.31570E-01	5.91060E-02	8.88700E-01	3.82810E-02
bSSA	8.95500E-01	2.60130E-02	9.87780E-01	1.83780E-02	9.57890E-01	2.81520E-02
bQGWO	9.09940E-01	3.26520E-02	9.92950E-01	1.22970E-02	9.91580E-01	1.46520E-02
bHHO	9.10220E-01	2.21880E-02	9.87780E-01	1.43390E-02	9.62190E-01	1.67140E-02
bSMA	8.84130E-01	2.70560E-02	9.71930E-01	2.06020E-02	9.49600E-01	2.82530E-02
	**Heart**		**JPNdata**		**SpectEW**	
bSRWPSO	**9.48150E-01**	4.68490E-02	**9.79460E-01**	**3.31550E-02**	**9.51260E-01**	3.97050E-02
bSCGWO	9.37040E-01	5.53490E-02	9.60830E-01	4.61140E-02	9.32760E-01	3.80800E-02
bGWO	9.33330E-01	4.55290E-02	9.52800E-01	5.51620E-02	9.44020E-01	4.70830E-02
bGSA	9.40740E-01	**3.98140E-02**	9.67920E-01	3.38530E-02	9.36300E-01	3.56080E-02
bPSO	9.37040E-01	4.63580E-02	9.67920E-01	3.38530E-02	9.36020E-01	3.63400E-02
bALO	9.40740E-01	4.99960E-02	9.67500E-01	7.07600E-02	9.36580E-01	4.30230E-02
bBA	7.07410E-01	1.41250E-01	8.47800E-01	9.91200E-02	8.00980E-01	9.10470E-02
bSSA	9.29630E-01	4.07590E-02	9.48690E-01	5.78260E-02	9.21750E-01	**3.09000E-02**
bQGWO	9.25930E-01	4.61930E-02	9.67500E-01	3.42920E-02	9.06530E-01	4.38240E-02
bHHO	9.44440E-01	4.70110E-02	9.67080E-01	4.68300E-02	9.24780E-01	3.56210E-02
bSMA	9.00000E-01	4.29450E-02	9.54520E-01	5.22390E-02	8.87680E-01	4.61800E-02
	**Vote**		**wdbc**		**Wielaw**	
bSRWPSO	**9.93330E-01**	2.10820E-02	**1.00000E+00**	**0.00000E+00**	**9.62300E-01**	3.07900E-02
bSCGWO	9.90110E-01	1.59310E-02	9.96460E-01	7.46360E-03	9.41490E-01	5.26440E-02
bGWO	9.79990E-01	2.29900E-02	9.98210E-01	5.64690E-03	9.50490E-01	4.64460E-02
bGSA	9.86770E-01	2.28800E-02	9.98210E-01	5.64690E-03	9.49790E-01	3.33510E-02
bPSO	9.93330E-01	**1.40800E-02**	9.96460E-01	7.46360E-03	9.45640E-01	3.94720E-02
bALO	9.90000E-01	2.24980E-02	9.94700E-01	1.19830E-02	9.41800E-01	**2.93180E-02**
bBA	8.54490E-01	1.46740E-01	9.57980E-01	2.74730E-02	7.67570E-01	9.36110E-02
bSSA	9.83330E-01	2.35700E-02	9.91230E-01	1.24070E-02	9.21290E-01	6.29560E-02
bQGWO	9.76540E-01	2.78200E-02	9.98250E-01	5.54790E-03	9.54320E-01	5.33510E-02
bHHO	9.89770E-01	1.64750E-02	9.94670E-01	8.57580E-03	9.49990E-01	4.30380E-02
bSMA	9.69870E-01	3.39470E-02	9.85960E-01	1.09940E-02	9.03960E-01	3.50090E-02

Bold values represent the optimal data.

**TABLE 14:** The results of Friedman ranking for accuracy.

Method	Rank-AVG	Rank
**bSRWPSO**	**1.22**	**1**
bSCGWO	5.33	7
bGWO	4.78	4
bGSA	4.22	2
bPSO	4.89	5
bALO	4.44	3
bBA	11.00	11
bSSA	8.44	9
bQGWO	5.22	6
bHHO	5.67	8
bSMA	9.89	10

Bold values represent the optimal data.

Table 15 analyzes the AVG and STD of the sensitivities of each method in this experiment. It can be seen that bSRWPSO performs the best on nine public datasets. Except for Australian, SpectEW, and Wielaw, the sensitivity of bSRWPSO on the other six public datasets is above 97%. Of course, bSRWPSO exhibits the highest number of smallest STD on the public datasets, indicating that the method has the relatively most stable adaptation to different classification problems. In addition, by codifying and analyzing the Friedman test results of each method on each public dataset, Table 16 compiles the Friedman mean values of each algorithm on the nine public datasets. According to the table, it can be seen that bSRWPSO is ranked first, which indicates that the sensitivity of this method is relatively the best among the methods involved in the comparison.

**TABLE 15:** The analysis results of sensitivity.

	Australian		BreastEW		Clean	
	AVG	STD	AVG	STD	AVG	STD
bSRWPSO	**9.34550E-01**	**2.26780E-02**	**1.00000E+00**	**0.00000E+00**	**1.00000E+00**	**0.00000E+00**
bSCGWO	9.05800E-01	3.99670E-02	9.91590E-01	1.35470E-02	9.96300E-01	1.17120E-02
bGWO	9.19100E-01	4.16640E-02	9.91590E-01	1.35470E-02	9.92590E-01	1.56160E-02
bGSA	9.32320E-01	4.77900E-02	9.97220E-01	8.78410E-03	9.88750E-01	1.81240E-02
bPSO	9.21660E-01	4.79750E-02	9.94440E-01	1.17120E-02	9.66520E-01	2.73530E-02
bALO	9.18960E-01	6.13740E-02	9.94440E-01	1.17120E-02	9.70230E-01	2.92590E-02
bBA	8.30770E-01	6.74060E-02	9.18810E-01	7.24990E-02	8.62250E-01	6.60620E-02
bSSA	8.98180E-01	2.85010E-02	9.88890E-01	2.68360E-02	9.51710E-01	3.91930E-02
bQGWO	9.11070E-01	3.98880E-02	9.97140E-01	9.03510E-03	9.92590E-01	1.56160E-02
bHHO	9.26920E-01	2.38930E-02	9.88810E-01	1.44490E-02	9.44160E-01	2.64270E-02
bSMA	8.75240E-01	7.85390E-02	9.74760E-01	2.07760E-02	9.44300E-01	3.13850E-02
	**Heart**		**JPNdata**		**SpectEW**	
bSRWPSO	**9.73330E-01**	4.66140E-02	**9.87500E-01**	**3.95280E-02**	**9.30000E-01**	1.63640E-01
bSCGWO	9.53330E-01	5.48850E-02	9.48210E-01	8.92660E-02	7.96670E-01	2.09910E-01
bGWO	9.53330E-01	6.32460E-02	9.48210E-01	6.70540E-02	8.30000E-01	2.39060E-01
bGSA	9.46670E-01	6.12620E-02	9.75000E-01	5.27050E-02	8.43330E-01	2.06110E-01
bPSO	9.46670E-01	**4.21640E-02**	9.48210E-01	6.70540E-02	8.00000E-01	2.24430E-01
bALO	9.53330E-01	5.48850E-02	9.58930E-01	9.45100E-02	8.33330E-01	2.27170E-01
bBA	7.66670E-01	1.26690E-01	8.14290E-01	1.29100E-01	5.40000E-01	3.02990E-01
bSSA	9.26670E-01	4.91910E-02	9.35710E-01	6.79720E-02	8.53330E-01	**1.45890E-01**
bQGWO	9.33330E-01	6.28540E-02	9.60710E-01	6.34420E-02	7.93330E-01	2.22110E-01
bHHO	9.53330E-01	5.48850E-02	9.73210E-01	5.66260E-02	8.46670E-01	1.82710E-01
bSMA	9.00000E-01	7.20080E-02	9.60710E-01	6.34420E-02	6.90000E-01	2.61080E-01
	**Vote**		**wdbc**		**Wielaw**	
bSRWPSO	**1.00000E+00**	**0.00000E+00**	**1.00000E+00**	**0.00000E+00**	**9.63640E-01**	6.35640E-02
bSCGWO	1.00000E+00	0.00000E+00	9.90480E-01	2.00780E-02	9.37880E-01	7.22950E-02
bGWO	9.91670E-01	2.63520E-02	9.95240E-01	1.50580E-02	9.56060E-01	6.07950E-02
bGSA	9.90910E-01	2.87480E-02	9.95240E-01	1.50580E-02	9.37880E-01	6.06900E-02
bPSO	1.00000E+00	0.00000E+00	9.95240E-01	1.50580E-02	9.37120E-01	6.10310E-02
bALO	1.00000E+00	0.00000E+00	9.95240E-01	1.50580E-02	9.27270E-01	7.17100E-02
bBA	8.80300E-01	1.76580E-01	9.62550E-01	4.85930E-02	7.59090E-01	1.44130E-01
bSSA	9.91670E-01	2.63520E-02	9.81170E-01	3.30960E-02	9.20450E-01	6.46040E-02
bQGWO	9.90910E-01	2.87480E-02	9.95240E-01	1.50580E-02	9.46210E-01	7.62990E-02
bHHO	1.00000E+00	0.00000E+00	9.95240E-01	1.50580E-02	9.36360E-01	9.63050E-02
bSMA	9.82580E-01	3.67770E-02	9.71860E-01	3.23470E-02	8.92420E-01	**3.92510E-02**

Bold values represent the optimal data.

**TABLE 16:** The results of Friedman ranking for sensitivity.

Method	Rank-AVG	Rank
**bSRWPSO**	**1.00**	**1**
bSCGWO	6.46	8
bGWO	4.69	5
bGSA	3.44	2
bPSO	4.56	7
bALO	4.91	4
bBA	10.81	11
bSSA	8.02	9
bQGWO	5.70	6
bHHO	2.87	3
bSMA	9.93	10

Bold values represent the optimal data.

Table 17 shows the AVG and STD of the MCC for bSRWPSO and other comparison algorithms. Except for Australian, heart, and SpectEW, the AVG of bSRWPSO on all other public datasets are between 0.92 and 1. Also, combining the STD obtained in the experiment, it is easy to find that the overall performance of bSRWPSO ranks first. As shown in Table 18, the Friedman average ranking of bSRWPSO based on 9 public datasets is also the first.

**TABLE 17:** The analysis results of MCC.

	Australian		BreastEW		Clean	
	AVG	STD	AVG	STD	AVG	STD
bSRWPSO	**8.48120E-01**	5.17560E-02	**9.88860E-01**	**1.79430E-02**	**9.95830E-01**	**1.31820E-02**
bSCGWO	8.26100E-01	6.38890E-02	9.77540E-01	2.63920E-02	9.95770E-01	1.33900E-02
bGWO	8.26540E-01	**4.32100E-02**	9.81680E-01	2.59030E-02	9.91750E-01	1.73950E-02
bGSA	8.40760E-01	5.30100E-02	9.84980E-01	2.62980E-02	9.78690E-01	3.62540E-02
bPSO	8.26900E-01	4.58720E-02	9.77650E-01	2.61900E-02	9.49530E-01	4.34090E-02
bALO	8.21080E-01	5.80410E-02	9.85170E-01	2.61290E-02	9.54200E-01	3.04300E-02
bBA	5.84490E-01	1.55890E-01	8.61480E-01	1.15560E-01	7.82360E-01	7.37280E-02
bSSA	7.90540E-01	5.31720E-02	9.74960E-01	3.70790E-02	9.15870E-01	5.59080E-02
bQGWO	8.20150E-01	6.70390E-02	9.85200E-01	2.59010E-02	9.83010E-01	2.96510E-02
bHHO	8.18390E-01	4.49810E-02	9.74140E-01	3.05360E-02	9.25690E-01	3.36130E-02
bSMA	7.74640E-01	5.17100E-02	9.40850E-01	4.34340E-02	8.98900E-01	5.69620E-02
	**Heart**		**JPNdata**		**SpectEW**	
bSRWPSO	**8.96910E-01**	9.48510E-02	**9.61400E-01**	**6.23070E-02**	**8.61440E-01**	1.18250E-01
bSCGWO	8.74190E-01	1.12500E-01	9.27550E-01	8.36580E-02	7.91790E-01	1.28190E-01
bGWO	8.69650E-01	8.86570E-02	9.07830E-01	1.09730E-01	8.22250E-01	1.74430E-01
bGSA	8.85560E-01	**7.72810E-02**	9.39360E-01	6.39860E-02	8.05400E-01	1.28490E-01
bPSO	8.74600E-01	9.29970E-02	9.39360E-01	6.39860E-02	8.00390E-01	1.30890E-01
bALO	8.83820E-01	9.96680E-02	9.35010E-01	1.41490E-01	8.13990E-01	1.34470E-01
bBA	3.94240E-01	3.34160E-01	6.99310E-01	1.97110E-01	3.99080E-01	2.92430E-01
bSSA	8.61220E-01	8.26300E-02	9.01670E-01	1.13360E-01	7.81110E-01	**8.44080E-02**
bQGWO	8.52680E-01	9.28470E-02	9.38670E-01	6.47090E-02	7.31620E-01	1.17960E-01
bHHO	8.88830E-01	9.50870E-02	9.38570E-01	8.66450E-02	7.89040E-01	9.09150E-02
bSMA	8.03760E-01	8.66060E-02	9.13850E-01	9.93440E-02	6.52020E-01	1.74390E-01
	**Vote**		**wdbc**		**Wielaw**	
bSRWPSO	**9.87290E-01**	4.02020E-02	**1.00000E+00**	**0.00000E+00**	**9.26930E-01**	6.01290E-02
bSCGWO	9.80070E-01	3.20940E-02	9.92490E-01	1.58390E-02	8.84240E-01	1.05990E-01
bGWO	9.59230E-01	4.75310E-02	9.96230E-01	1.19370E-02	9.02450E-01	9.25300E-02
bGSA	9.73750E-01	4.48750E-02	9.96230E-01	1.19370E-02	9.02900E-01	6.46410E-02
bPSO	9.86560E-01	**2.83560E-02**	9.92560E-01	1.56880E-02	8.93380E-01	7.87510E-02
bALO	9.80480E-01	4.34470E-02	9.88810E-01	2.54420E-02	8.87420E-01	**5.69730E-02**
bBA	7.08580E-01	2.98890E-01	9.12290E-01	5.84740E-02	5.48000E-01	1.83860E-01
bSSA	9.66850E-01	4.60580E-02	9.81510E-01	2.61840E-02	8.47970E-01	1.19710E-01
bQGWO	9.52450E-01	5.66270E-02	9.96260E-01	1.18200E-02	9.10190E-01	1.06740E-01
bHHO	9.79460E-01	3.30840E-02	9.88850E-01	1.79480E-02	9.03540E-01	8.31280E-02
bSMA	9.39480E-01	6.88960E-02	9.70510E-01	2.30210E-02	8.08880E-01	7.00230E-02

Bold values represent the optimal data.

**TABLE 18:** The results of Friedman ranking for MCC.

Method	Rank-AVG	Rank
**bSRWPSO**	**1.04**	**1**
bSCGWO	6.11	7
bGWO	3.94	4
bGSA	3.85	2
bPSO	4.85	5
bALO	4.56	3
bBA	11.00	11
bSSA	8.39	9
bQGWO	5.74	6
bHHO	6.33	8
bSMA	9.98	10

Bold values represent the optimal data.

Table 19 analyzes the performance of bSRWPSO on nine public datasets based on the F-measure and gives the AVG reflecting its average capability and the STD reflecting its stability. Looking at Table 19, it is easy to see that the minimum mean of bSRWPSO occurs on the public dataset SpectEW, but it is also above 0.88. Overall, it shows a general ability greater than 0.95 and ranks first on each dataset. The comprehensive Friedman ranking for the F-measure in this experiment is given in Table 20. Among them, bSRWPSO ranks first among the 11 compared algorithms with an average ranking of 1 and is smaller than the average ranking of the second-ranked bGSA by 3. While bPSO ranks fourth with a score of 4.56. Therefore, it can be concluded that the three improvement strategies introduced in this paper improve the classification performance of bPSO in a significant way.

**TABLE 19:** The analysis results of F-measure.

	Australian		BreastEW		Clean	
	AVG	STD	AVG	STD	AVG	STD
bSRWPSO	**9.32450E-01**	2.23060E-02	**9.95850E-01**	**6.68000E-03**	**9.98180E-01**	**5.74960E-03**
bSCGWO	9.20180E-01	2.89040E-02	9.91550E-01	9.91450E-03	9.98110E-01	5.96660E-03
bGWO	9.21330E-01	2.00810E-02	9.92990E-01	9.87090E-03	9.96230E-01	7.95540E-03
bGSA	9.28150E-01	2.40040E-02	9.94480E-01	9.67520E-03	9.90560E-01	1.60430E-02
bPSO	9.21410E-01	2.09180E-02	9.91670E-01	9.71270E-03	9.77280E-01	1.95560E-02
bALO	9.18250E-01	3.22590E-02	9.94440E-01	9.71210E-03	9.79230E-01	1.40450E-02
bBA	8.18380E-01	6.31720E-02	9.42780E-01	5.19260E-02	8.96390E-01	3.87220E-02
bSSA	9.05390E-01	2.20070E-02	9.90130E-01	1.50700E-02	9.62240E-01	2.53790E-02
bQGWO	9.18410E-01	2.87380E-02	9.94440E-01	9.60140E-03	9.92590E-01	1.29500E-02
bHHO	9.19720E-01	**1.97830E-02**	9.90220E-01	1.14610E-02	9.65720E-01	1.47240E-02
bSMA	8.92330E-01	2.90680E-02	9.77610E-01	1.63650E-02	9.54980E-01	2.54200E-02
	**Heart**		**JPNdata**		**SpectEW**	
bSRWPSO	**9.54390E-01**	4.16280E-02	**9.80000E-01**	**3.22030E-02**	**8.81050E-01**	1.07180E-01
bSCGWO	9.44040E-01	4.85080E-02	9.58810E-01	4.91090E-02	8.16370E-01	1.24640E-01
bGWO	9.40680E-01	4.17140E-02	9.53970E-01	5.25700E-02	8.38000E-01	1.93070E-01
bGSA	9.46680E-01	**3.50180E-02**	9.69020E-01	3.27820E-02	8.30680E-01	1.26140E-01
bPSO	9.44010E-01	4.02680E-02	9.66430E-01	3.56500E-02	8.20660E-01	1.33040E-01
bALO	9.47410E-01	4.38540E-02	9.64420E-01	7.90370E-02	8.32400E-01	1.31910E-01
bBA	7.48270E-01	1.02980E-01	8.39690E-01	1.09160E-01	5.00180E-01	2.68920E-01
bSSA	9.36210E-01	3.61090E-02	9.48780E-01	5.53930E-02	8.17050E-01	**6.73170E-02**
bQGWO	9.32820E-01	4.34480E-02	9.66430E-01	3.56500E-02	7.66360E-01	1.23790E-01
bHHO	9.49860E-01	4.28710E-02	9.67860E-01	4.32360E-02	8.17920E-01	8.10720E-02
bSMA	9.08500E-01	3.99230E-02	9.55360E-01	5.03900E-02	6.91280E-01	1.82300E-01
	**Vote**		**wdbc**		**Wielaw**	
bSRWPSO	**9.92310E-01**	2.43250E-02	**1.00000E+00**	**0.00000E+00**	**9.59100E-01**	3.50840E-02
bSCGWO	9.87650E-01	1.99040E-02	9.95120E-01	1.02840E-02	9.37390E-01	5.52330E-02
bGWO	9.74970E-01	2.91750E-02	9.97560E-01	7.71290E-03	9.47360E-01	4.97040E-02
bGSA	9.83550E-01	2.80420E-02	9.97560E-01	7.71290E-03	9.45680E-01	3.63700E-02
bPSO	9.91650E-01	**1.76180E-02**	9.95240E-01	1.00480E-02	9.40980E-01	4.39230E-02
bALO	9.87960E-01	2.65790E-02	9.93020E-01	1.58940E-02	9.36250E-01	**3.30590E-02**
bBA	8.25720E-01	1.74520E-01	9.44640E-01	3.71620E-02	7.51270E-01	9.68510E-02
bSSA	9.79260E-01	2.84580E-02	9.87910E-01	1.73680E-02	9.18000E-01	6.26950E-02
bQGWO	9.70230E-01	3.56460E-02	9.97560E-01	7.71290E-03	9.50030E-01	5.87630E-02
bHHO	9.87300E-01	2.04640E-02	9.92910E-01	1.14200E-02	9.43360E-01	5.27690E-02
bSMA	9.62920E-01	4.19900E-02	9.80930E-01	1.50800E-02	8.97020E-01	3.72510E-02

Bold values represent the optimal data.

**TABLE 20:** The results of Friedman ranking for F-measure.

Method	Rank-AVG	Rank
**bSRWPSO**	**1.00**	**1**
bSCGWO	5.89	7
bGWO	4.67	5
bGSA	4.00	2
bPSO	4.56	4
bALO	4.44	3
bBA	11.00	11
bSSA	8.44	9
bQGWO	5.56	6
bHHO	6.00	8
bSMA	10.00	10

Bold values represent the optimal data.

In summary, this section verifies the comprehensive performance of bSRWPSO in feature selection experiments by analyzing classification accuracy, sensitivity, MCC, and F-measure. Comparing ten other methods demonstrates that the classification capability of bSRWPSO has a strong core competitive advantage. Therefore, the bSRWPSO proposed in this paper is a novel method with a more substantial classification capability that can be used for feature selection.

### 7.3. AD dataset experiments

#### 7.3.1. AD dataset

This medical dataset includes 181 patients enrolled at the Department of Dermatology at the Affiliated Hospital of Medical School, Ningbo University, from May 2021 to March 2022 diagnosed with AD. The primary demographic data such as sex and age are included, and the typical laboratory characteristics comprising the blood routine examination, blood serum allergen test, and Total IgE in serum are gathered. The clinical and laboratory results of the patients with AD are demonstrated in Table 21. Continuous data are expressed as means ± standard deviation. Categorical data are described as percentages. The Ethics Commission of the Affiliated Hospital of Medical School approved this medical dataset (NO. KY20191208).

**TABLE 21:** The characteristics dataset of 181 patients with AD.

Classes	No.	Features	Results
	F1	Gender [male/female, *n* (%)]	75 (40.8)/106 (57.6)
	F2	Age (months, $\bar{x}\pm$ s)	275.27 ± 255.16
The blood routine examination	F3	White blood cells (WBC) (× 10^9^/L, $\bar{x}\pm$ s)	7.32 ± 2.42
	F4	The content of NE (× 10^9^/L, $\bar{x}\pm$ s)	3.95 ± 4.36
	F5	The content of LY (× 10^9^/L, $\bar{x}\pm$ s)	2.93 ± 1.80
	F6	The content of MO (× 10^9^/L, $\bar{x}\pm$ s)	0.44 ± 0.21
	F7	The content of EO (× 10^9^/L, $\bar{x}\pm$ s)	0.29 ± 0.27
	F8	The content of BASO (× 10^9^/L, $\bar{x}\pm$ s)	0.03 ± 0.03
	F9	The percentage of neutrophils (NE) in WBC (%, $\bar{x}\pm$ s)	50.97 ± 16.75
	F10	The percentage of lymphocytes (LY) in WBC (%, $\bar{x}\pm$ s)	38.94 ± 15.78
	F11	The percentage of monocytes (MO) in WBC (%, $\bar{x}\pm$ s)	6.43 ± 5.84
	F12	The percentage of eosinophils (EO) in WBC (%, $\bar{x}\pm$ s)	3.66 ± 2.91
	F13	The percentage of basophils (BASO) in WBC (%, $\bar{x}\pm$ s)	0.68 ± 0.09
	F14	Hypersensitive c-reactive protein (hs-CRP) (mg/L, $\bar{x}\pm$ s)	2.61 ± 5.78
The blood serum allergen test	F15	Cat dander (IU/ml, $\bar{x}\pm$ s)	0.13 ± 0.51
	F16	Cockroach (IU/ml, $\bar{x}\pm$ s)	0.15 ± 0.69
	F17	Mildew (IU/ml, $\bar{x}\pm$ s)	0.14 ± 0.93
	F18	Pollen (IU/ml, $\bar{x}\pm$ s)	0.12 ± 0.08
	F19	Egg white (IU/ml, $\bar{x}\pm$ s)	0.35 ± 0.93
	F20	Milk (IU/ml, $\bar{x}\pm$ s)	0.31 ± 0.95
	F21	Shrimp (IU/ml, $\bar{x}\pm$ s)	0.17 ± 1.07
	F22	Dermatophagoides Pteronyssinus/Farinae (IU/ml, $\bar{x}\pm$ s)	2.89 ± 11.22
	F23	Dog dander (IU/ml, $\bar{x}\pm$ s)	0.09 ± 0.08
	F24	Crab (IU/ml, $\bar{x}\pm$ s)	0.18 ± 1.13
	F25	House dust (IU/ml, $\bar{x}\pm$ s)	0.08 ± 0.09
	F26	Beef (IU/mL, $\bar{x}\pm$ s)	0.07 ± 0.09
	F27	Ragweed (IU/ml, $\bar{x}\pm$ s)	0.14 ± 0.11
	F28	Peanut (IU/ml, $\bar{x}\pm$ s)	0.14 ± 0.10
	F29	Lobster/Scallop (IU/ml, $\bar{x}\pm$ s)	0.24 ± 1.52
	F30	Cod (IU/ml, $\bar{x}\pm$ s)	0.15 ± 0.89
	F31	Salmon (IU/ml, $\bar{x}\pm$ s)	0.08 ± 0.08
	F32	Mutton (IU/ml, $\bar{x}\pm$ s)	0.06 ± 0.07
	F33	Mugwort (IU/ml, $\bar{x}\pm$ s)	0.08 ± 0.09
	F34	Humulus (IU/ml, $\bar{x}\pm$ s)	0.13 ± 0.10
	F35	Soy (IU/ml, $\bar{x}\pm$ s)	0.11 ± 0.09
	F36	Total IgE (IU/ml, $\bar{x}\pm$ s)	119.87 ± 216.28

#### 7.3.2. Medical validation experiments

To further demonstrate the classification capability of bSRWPSO, this section sets up four comparison experiments on bSRWPSO based on a specific medical dataset. First, to illustrate the core advantage of the combination of bSRWPSO and FKNN in feature selection, this section sets up comparison experiments by making bSRWPSO combined with FKNN, kernel extreme learning machine (KELM), KNN, SVM, and MLP, respectively. Then, to verify that the classification ability of the bSRWPSO-FKNN is better than that of the classical classifier, this section makes the comparison experiments and analysis of bSRWPSO-FKNN with five classical classifiers based on a specific medical dataset, mainly including BP, CART, RandomF, AdaBoost, and ELMforFS. Next, this section makes bSRWPSO, and ten other binary versions of the swarm intelligence optimization algorithm combined with FKNN, respectively, and the classification advantages of the bSRWPSO-FKNN are verified by setting up feature classification comparison experiments. Finally, this section uses the bSRWPSO-FKNN to set up ten times 10-fold cross-validation experiments on a medical dataset. As a result, it successfully extracts the key features affecting the onset of AD.

**FIGURE 16:**
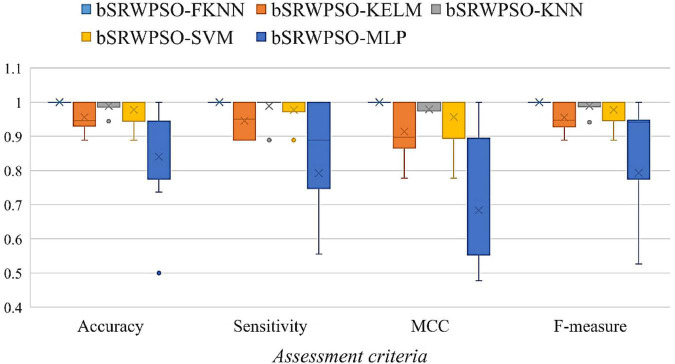
Evaluation results for different combinations.

Figure 16 shows the analysis of the results of the comparative experiments combining the bSRWPSO with each of the five machine learning algorithms. As seen from the box plots, the bSRWPSO-FKNN obtains the most concentrated experimental results among the four evaluation criteria, indicating that the classification ability of the model is relatively the most stable in this experiment. In the figure, the marker × represents the average value of each group of data. Therefore, it is easy to observe that the average value of all four evaluation methods for the combination of bSRWPSO and FKNN is 1 and greater than the other four combinations, indicating that the classification performance of the bSRWPSO-FKNN is the best on this medical dataset.

**FIGURE 17:**
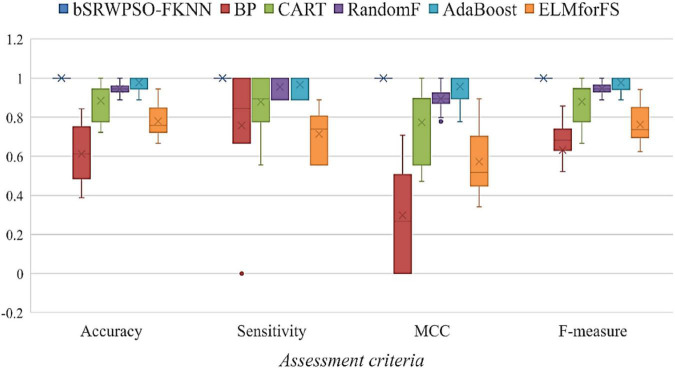
The results of bSRWPSO with classical classifiers.

Figure 17 shows the results of comparing the bSRWPSO-FKNN with five classical classifiers. Combined with the characteristics of the box plot, it can be noticed that the bSRWPSO-FKNN has an undeniable competitive advantage in this comparison. bSRWPSO-FKNN has a relatively more stable classification performance and is the best in terms of comprehensive classification ability. On the contrary, the evaluation results of the other five classical classifiers in all four evaluation criteria are relatively scattered, indicating that the classification ability of these classical methods is unstable. Therefore, the bSRWPSO-FKNN still has the core competitive advantage in this experiment.

**FIGURE 18:**
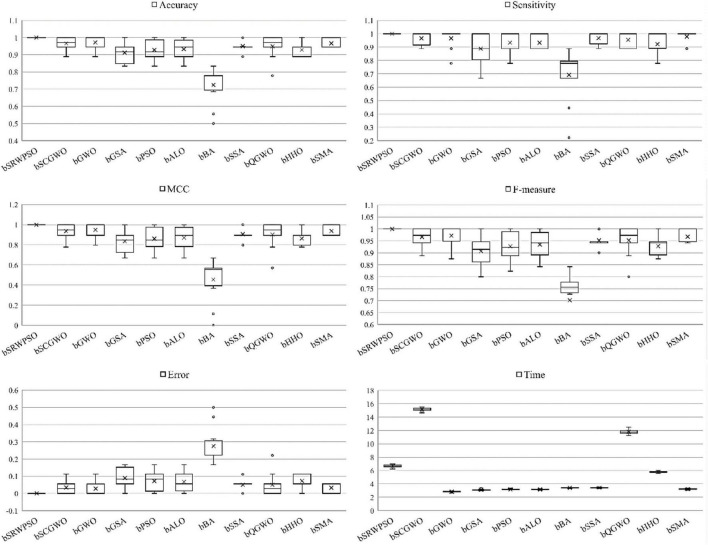
Comparison results of bSRWPSO with other binary algorithms.

Figure 18 shows the experimental results comparing bSRWPSO with ten other binary swarm intelligence algorithms. The figure shows the analysis results based on six evaluation criteria through six box plots, including Accuracy, Sensitivity, MCC, F-measure, Error, and Time. The error indicates the error rate of the classification method, and the sum of classification accuracy is 1. Time reflects the time spent by the classification method on feature experiments, and the larger the value, the more time the classification method consumes to extract key features successfully. Comparing the Accuracy, Sensitivity, MCC, and F-measure in the figure, we can see that bSRWPSO has the largest experimental results, which indicates that bSRWPSO has the most successful combination with FKNN among all the swarm intelligence optimization algorithms involved in the feature experiments. Its classification performance is not only the best but also the most stable. Comparing the Error in the figure, it is easy to find that bSRWPSO has the slightest possibility of an error during the experiments. However, by comparing the time of the 11 methods, it can be found that bSRWPSO has some shortcomings in time complexity; although it is much lower than the two methods, bSCGWO and bQGWO, in terms of time cost, it is higher than the other eight compared methods.

To further demonstrate the core advantages of the bSRWPSO-FKNN, Table 22 gives the average (AVG) and ranking results (Rank) of the Friedman test based on Accuracy, Sensitivity, MCC, and F-measure. According to the table, it is not difficult to conclude that bSRWPSO obtained the minimum average in all four evaluation criteria, indicating its first ranking in each criterion.

**TABLE 22:** The results of the Friedman test.

	Accuracy		Sensitivity		MCC		F-measure	
	AVG	Rank	AVG	Rank	AVG	Rank	AVG	Rank
**bSRWPSO**	**3.1**	**1**	**4.05**	**1**	**3.1**	**1**	**3.1**	**1**
bSCGWO	4.5	4	5.65	6	4.45	4	4.7	5
bGWO	4	2	4.5	3	4	2	4.1	2
bGSA	7.9	10	7.3	10	8	10	7.9	10
bPSO	7	8	6.95	8	6.95	8	7.15	9
bALO	7.05	9	7.05	9	7.15	9	7.05	8
bBA	10.95	11	10.45	11	10.95	11	10.9	11
bSSA	6.55	7	5.25	5	6.5	7	6.2	6
bQGWO	4.6	5	4.6	4	4.6	5	4.55	4
bHHO	6.2	6	5.8	7	6.15	6	6.2	6
bSMA	4.15	3	4.4	2	4.15	3	4.15	3

Bold values represent the optimal data.

**FIGURE 19:**
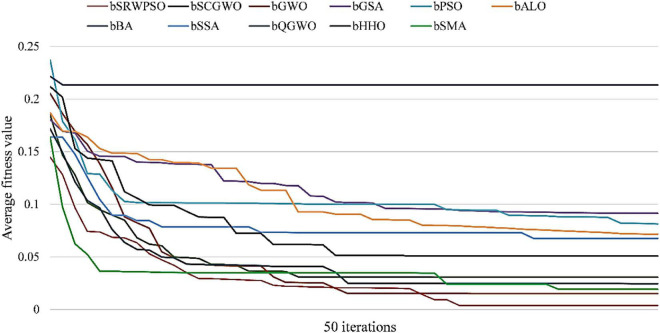
Convergence curve of feature selection method.

As shown in Figure 19, the convergence curve of bSRWPSO is lower than other methods’ convergence curves after reaching the maximum number of iterations, which means that bSRWPSO has the relatively best optimization accuracy among the methods involved in the experiments. Therefore, bSRWPSO is the most effective in optimizing the classification ability of FKNN.

**FIGURE 20:**
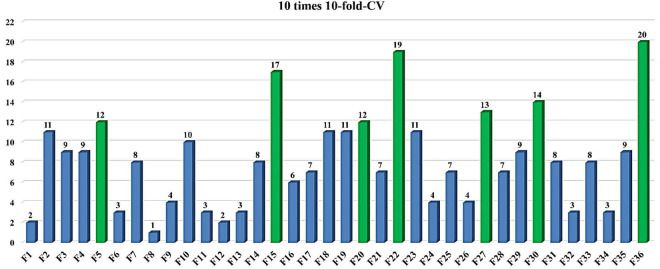
The results of 10 times 10-fold crossover experiments.

Figure 20 shows the experimental results of bSRWPSO for ten times 10-fold cross-validation on the AD dataset. In the figure, the vertical axis indicates the number of times each attribute was selected and the horizontal axis indicates the different attributes in AD. From the figure, it is easy to find that features F5, F15, F20, F22, F27, F30, and F36 were selected by bSRWPSO-FKNN, denoting the content of LY, Cat dander, Milk, Dermatophagoides Pteronyssinus/Farinae, Ragweed, Cod, and Total IgE, respectively. Combined with clinical medicine, it was concluded that these features have medical reference value. Therefore, this experiment proves that bSRWPSO-FKNN is scientific and practical for predicting AD development.

## 8. Conclusion and future works

This study puts forward an improved algorithm according to SOB, RRS, AWS, and PSO, called SRWPSO. Next, we propose a binary version of the SRWPSO, called bSRWPSO. Then, we make bSRWPSO combined with FKNN to offer a novel feature prediction model called bSRWPSO-FKNN. In SRWPSO, the SOB improves the quality of the initial swarm and improves the algorithm’s traversal of the initial population space. The RRS boosts the capability of the original PSO to get rid of the local optimum, which enhances the original PSO’s convergence accuracy. The AWS perturbs the algorithm according to its optimization search process and enhances the exploration ability of the algorithm by controlling the displacement vector. The RRS, in conjunction with the AWS, improves the diversity of the particle swarm, which in turn enhances the exploration and exploitation capability of the original PSO. In addition, based on the performance analysis experiments of SRWPSO and the original PSO, it can be concluded that the combination of the three improved strategies not only increases the population diversity of the original PSO but also balances the exploration and exploitation of the original PSO, which in turn leads to a stronger convergence capability of the original PSO. By analyzing the comparison results of SRWPSO, the original PSO, the nine original algorithms, and the nine improved algorithms on 30 benchmark functions, it is easy to conclude that the SRWPSO’s core advantages are faster convergence speed, higher convergence accuracy, and greater ability to escape local optimal solutions. For bSRWPSO, this paper introduces a V-shaped binary transformation method in SRWPSO to successfully discretize SRWPSO. In bSRWPSO-FKNN, when doing feature selection experiments, the model first optimizes the datasets participating in the experiments by bSRWPSO to obtain the optimization subsets. Then, the model performs classification prediction on the optimized subsets by FKNN. In order to verify the classification capability of bSRWPSO-FKNN, a series of performance validation experiments are conducted on the model in this paper, which successfully demonstrates that the performance of the model is relatively the best among the methods involved in the experiments. In addition, we conducted ten times 10-fold cross-validation experiments on bSRWPSO-FKNN based on a specific medical dataset in this paper and successfully extracted the key features affecting the onset of AD, mainly including the content of lymphocytes (LY), Cat dander, Milk, Dermatophagoides Pteronyssinus/Farinae, Ragweed, Cod and Total IgE. Finally, we enabled the selected key features to be discussed and analyzed in conjunction with clinical practice, demonstrating that the bSRWPSO-FKNN possesses practical medical significance.

However, the approach proposed in this paper is flawed. While the three improvement strategies greatly enhance the performance of the original PSO, they increase the time complexity of the original PSO, which means that the SRWPSO will solve the problem at a higher cost than the original PSO. Since bSRWPSO-FKNN is proposed based on SRWPSO, the increase in time complexity of the model is inevitable, which can be seen in Figure 18. Therefore, reducing the time complexity of this study will be one of the essential works in the future. In addition, we can use more effective strategies to improve the original PSO in future work.

## Data availability statement

The original contributions presented in this study are included in the article/Supplementary material, further inquiries can be directed to the corresponding authors.

## Author contributions

YL, ZX, AH, XJ, ZL, MW, and QZ: writing—original draft, writing—review and editing, software, visualization, and investigation. DZ, HC, and SX: conceptualization, methodology, formal analysis, investigation, writing—review and editing, funding acquisition, and supervision. All authors contributed to the article and approved the submitted version.
