# Supplementary material for: bSRWPSO-FKNN: A boosted PSO with fuzzy K-nearest neighbor classifier for predicting atopic dermatitis disease
Source: Front Neuroinform. 2023 Jan 16;16:1063048. doi: 10.3389/fninf.2022.1063048 (PMC9884708; doi:10.3389/fninf.2022.1063048)
Supplement: Supplementary file 1 [file Table_1.docx]

# Appendix A

**Table 1.** The experimental results of different versions

|  | F1 |  | F2 |  | F3 |  |
| --- | --- | --- | --- | --- | --- | --- |
|  | AVG | STD | AVG | STD | AVG | STD |
| SRWPSO | **1.64708E+05** | **1.13331E+05** | **2.28558E+02** | **2.76229E+01** | 3.00290E+02 | 6.09432E-01 |
| SRPSO | 1.26920E+07 | 3.59560E+06 | 9.79185E+07 | 2.41760E+07 | 9.04260E+02 | 2.19249E+02 |
| SWPSO | 7.87782E+05 | 9.12053E+05 | 1.41762E+07 | 7.76155E+07 | 3.00925E+02 | 1.38066E+00 |
| RWPSO | 3.54783E+05 | 5.11235E+05 | 2.32304E+02 | 3.38013E+01 | **3.00095E+02** | **1.18108E-01** |
| SOBPSO | 8.71795E+06 | 2.09504E+06 | 2.24749E+08 | 1.79491E+08 | 9.37397E+02 | 1.00364E+02 |
| RRPSO | 1.41426E+07 | 5.44182E+06 | 1.01507E+08 | 2.94061E+07 | 7.91902E+02 | 1.95767E+02 |
| AWPSO | 1.73113E+06 | 1.98454E+06 | 3.68725E+07 | 1.44285E+08 | 3.00780E+02 | 1.01216E+00 |
| PSO | 8.44036E+06 | 2.33759E+06 | 1.43611E+08 | 1.49967E+07 | 9.33042E+02 | 1.10029E+02 |
|  | F4 |  | F5 |  | F6 |  |
|  | AVG | STD | AVG | STD | AVG | STD |
| SRWPSO | **4.56052E+02** | 3.02987E+01 | 5.20006E+02 | 1.78681E-02 | 6.18582E+02 | **2.30170E+00** |
| SRPSO | 5.45777E+02 | 4.72398E+01 | 5.20496E+02 | 6.25480E-02 | 6.22198E+02 | 3.38221E+00 |
| SWPSO | 4.85470E+02 | 2.56940E+01 | 5.20796E+02 | 8.62913E-02 | **6.15180E+02** | 2.96133E+00 |
| RWPSO | 4.69539E+02 | **2.39338E+01** | **5.20000E+02** | **1.48654E-04** | 6.18038E+02 | 2.68751E+00 |
| SOBPSO | 5.17704E+02 | 2.85070E+01 | 5.20938E+02 | 5.52685E-02 | 6.21679E+02 | 2.82889E+00 |
| RRPSO | 5.29781E+02 | 2.81327E+01 | 5.20498E+02 | 8.80816E-02 | 6.21099E+02 | 3.09953E+00 |
| AWPSO | 4.82556E+02 | 3.20934E+01 | 5.20826E+02 | 9.73977E-02 | 6.17051E+02 | 3.17187E+00 |
| PSO | 4.68415E+02 | 3.32626E+01 | 5.20941E+02 | 4.41663E-02 | 6.22021E+02 | 2.95767E+00 |
|  | F7 |  | F8 |  | F9 |  |
|  | AVG | STD | AVG | STD | AVG | STD |
| SRWPSO | **7.00010E+02** | **1.13614E-02** | 8.66861E+02 | 1.45230E+01 | 1.03170E+03 | 2.21548E+01 |
| SRPSO | 7.01921E+02 | 2.84698E-01 | 9.07045E+02 | 1.83321E+01 | 1.08124E+03 | 2.45215E+01 |
| SWPSO | 7.01405E+02 | 3.27263E+00 | 9.07918E+02 | 1.58729E+01 | 1.07213E+03 | 2.17481E+01 |
| RWPSO | 7.00019E+02 | 1.95759E-02 | **8.61659E+02** | 1.37644E+01 | **9.87490E+02** | 2.19277E+01 |
| SOBPSO | 7.04493E+02 | 4.65071E+00 | 9.63389E+02 | **1.23560E+01** | 1.13821E+03 | **1.67062E+01** |
| RRPSO | 7.01859E+02 | 2.61577E-01 | 9.00610E+02 | 2.12657E+01 | 1.04679E+03 | 2.82075E+01 |
| AWPSO | 7.04143E+02 | 5.71490E+00 | 9.23321E+02 | 2.15361E+01 | 1.03827E+03 | 2.92775E+01 |
| PSO | 7.02324E+02 | 1.19736E-01 | 9.69655E+02 | 1.87470E+01 | 1.11495E+03 | 2.87648E+01 |
|  | F10 |  | F11 |  | F12 |  |
|  | AVG | STD | AVG | STD | AVG | STD |
| SRWPSO | **2.49936E+03** | 5.16713E+02 | 3.57935E+03 | **4.77004E+02** | 1.20023E+03 | 8.93177E-02 |
| SRPSO | 3.43309E+03 | 5.28518E+02 | 5.01228E+03 | 6.23842E+02 | 1.20073E+03 | 1.44637E-01 |
| SWPSO | 4.09024E+03 | 5.96897E+02 | 4.42311E+03 | 5.46276E+02 | 1.20019E+03 | **7.45221E-02** |
| RWPSO | 2.63639E+03 | **4.44807E+02** | **3.38568E+03** | 4.93765E+02 | 1.20022E+03 | 7.70405E-02 |
| SOBPSO | 5.18015E+03 | 5.06472E+02 | 5.88984E+03 | 5.46891E+02 | 1.20236E+03 | 2.41785E-01 |
| RRPSO | 3.35592E+03 | 5.09075E+02 | 4.92753E+03 | 5.34712E+02 | 1.20079E+03 | 1.53667E-01 |
| AWPSO | 3.77452E+03 | 5.66319E+02 | 4.14365E+03 | 7.24311E+02 | **1.20018E+03** | 8.66316E-02 |
| PSO | 5.17497E+03 | 5.15206E+02 | 5.90413E+03 | 5.43338E+02 | 1.20251E+03 | 2.46452E-01 |
|  | F13 |  | F14 |  | F15 |  |
|  | AVG | STD | AVG | STD | AVG | STD |
| SRWPSO | 1.30036E+03 | 9.40553E-02 | 1.40028E+03 | 7.65662E-02 | 1.50673E+03 | 2.78852E+00 |
| SRPSO | 1.30036E+03 | 7.38014E-02 | 1.40024E+03 | 5.06317E-02 | 1.51621E+03 | 3.77027E+00 |
| SWPSO | 1.30045E+03 | 1.31466E-01 | 1.40134E+03 | 5.32360E+00 | 1.50646E+03 | 4.18992E+00 |
| RWPSO | 1.30035E+03 | **5.80808E-02** | 1.40025E+03 | **3.89026E-02** | **1.50632E+03** | 2.57681E+00 |
| SOBPSO | 1.30040E+03 | 8.07885E-02 | 1.40064E+03 | 1.74072E+00 | 1.51653E+03 | **1.07992E+00** |
| RRPSO | 1.30036E+03 | 7.96726E-02 | **1.40024E+03** | 5.07203E-02 | 1.51490E+03 | 3.30725E+00 |
| AWPSO | 1.30048E+03 | 1.39367E-01 | 1.40040E+03 | 2.40299E-01 | 1.50744E+03 | 4.17131E+00 |
| PSO | **1.30034E+03** | 8.79482E-02 | 1.40031E+03 | 1.51996E-01 | 1.51681E+03 | 1.23976E+00 |
|  | F16 |  | F17 |  | F18 |  |
|  | AVG | STD | AVG | STD | AVG | STD |
| SRWPSO | **1.61048E+03** | 7.58576E-01 | 3.67287E+04 | 3.75853E+04 | 5.01220E+03 | 3.48548E+03 |
| SRPSO | 1.61092E+03 | 5.75277E-01 | 4.31101E+05 | 3.14203E+05 | 1.36056E+06 | 5.94489E+05 |
| SWPSO | 1.61167E+03 | 7.34198E-01 | **3.52231E+04** | **1.11375E+04** | 8.04530E+03 | **2.98598E+03** |
| RWPSO | 1.61075E+03 | 5.57983E-01 | 1.32813E+05 | 3.12390E+05 | 5.28938E+03 | 3.05342E+03 |
| SOBPSO | 1.61187E+03 | 4.87851E-01 | 2.04142E+05 | 7.67477E+04 | 2.04412E+06 | 5.69051E+05 |
| RRPSO | 1.61102E+03 | 5.42786E-01 | 5.57060E+05 | 4.53656E+05 | 1.33580E+06 | 6.38585E+05 |
| AWPSO | 1.61168E+03 | 6.36328E-01 | 8.41240E+04 | 5.36276E+04 | **4.16212E+03** | 3.32245E+03 |
| PSO | 1.61194E+03 | **4.19940E-01** | 2.33857E+05 | 1.19839E+05 | 2.10968E+06 | 5.65008E+05 |
|  | F19 |  | F20 |  | F21 |  |
|  | AVG | STD | AVG | STD | AVG | STD |
| SRWPSO | 1.91870E+03 | 1.74590E+01 | 2.22097E+03 | 1.00939E+02 | 2.57273E+04 | **1.80724E+04** |
| SRPSO | **1.91684E+03** | 1.07480E+01 | 2.28360E+03 | 5.70935E+02 | 1.61199E+05 | 1.22522E+05 |
| SWPSO | 1.92988E+03 | 2.75965E+01 | **2.17641E+03** | **4.92906E+01** | **2.30687E+04** | 1.94856E+04 |
| RWPSO | 1.92052E+03 | 2.03382E+01 | 2.47345E+03 | 2.91899E+02 | 3.42728E+04 | 2.32781E+04 |
| SOBPSO | 1.92402E+03 | 2.20269E+01 | 2.31184E+03 | 7.31639E+01 | 7.61011E+04 | 5.11145E+04 |
| RRPSO | 1.91883E+03 | 1.44238E+01 | 2.22144E+03 | 8.09384E+01 | 1.33554E+05 | 1.11334E+05 |
| AWPSO | 1.91763E+03 | 1.78652E+01 | 2.20852E+03 | 5.81601E+01 | 5.95534E+04 | 8.08845E+04 |
| PSO | 1.91715E+03 | **2.14162E+00** | 2.32599E+03 | 5.61966E+01 | 1.03230E+05 | 6.24794E+04 |
|  | F22 |  | F23 |  | F24 |  |
|  | AVG | STD | AVG | STD | AVG | STD |
| SRWPSO | **2.69519E+03** | 2.19190E+02 | **2.50000E+03** | **0.00000E+00** | **2.60000E+03** | **0.00000E+00** |
| SRPSO | 2.77128E+03 | 2.01330E+02 | 2.50000E+03 | 0.00000E+00 | 2.60000E+03 | 0.00000E+00 |
| SWPSO | 2.87205E+03 | **1.28283E+02** | 2.50000E+03 | 0.00000E+00 | 2.60000E+03 | 0.00000E+00 |
| RWPSO | 2.75787E+03 | 1.77172E+02 | 2.61545E+03 | 8.88919E-01 | 2.63176E+03 | 6.71347E+00 |
| SOBPSO | 2.91612E+03 | 1.58141E+02 | 2.50000E+03 | 0.00000E+00 | 2.60000E+03 | 0.00000E+00 |
| RRPSO | 2.71537E+03 | 1.90658E+02 | 2.62000E+03 | 1.65589E+00 | 2.63597E+03 | 6.78712E+00 |
| AWPSO | 2.84029E+03 | 2.11738E+02 | 2.61625E+03 | 2.31930E+00 | 2.62015E+03 | 9.11589E+00 |
| PSO | 2.89862E+03 | 1.82638E+02 | 2.61593E+03 | 6.44496E-01 | 2.62721E+03 | 5.30604E+00 |
|  | F25 |  | F26 |  | F27 |  |
|  | AVG | STD | AVG | STD | AVG | STD |
| SRWPSO | **2.70000E+03** | **0.00000E+00** | 2.70036E+03 | 7.04005E-02 | **2.90000E+03** | **0.00000E+00** |
| SRPSO | 2.70000E+03 | 0.00000E+00 | **2.70035E+03** | **5.82364E-02** | 2.90000E+03 | 0.00000E+00 |
| SWPSO | 2.70000E+03 | 0.00000E+00 | 2.70045E+03 | 1.02905E-01 | 2.90000E+03 | 0.00000E+00 |
| RWPSO | 2.71729E+03 | 4.60513E+00 | 2.72697E+03 | 4.48176E+01 | 3.40413E+03 | 1.93681E+02 |
| SOBPSO | 2.70000E+03 | 0.00000E+00 | 2.70038E+03 | 1.19929E-01 | 2.90000E+03 | 0.00000E+00 |
| RRPSO | 2.72056E+03 | 5.05892E+00 | 2.75393E+03 | 5.09575E+01 | 3.45542E+03 | 2.31012E+02 |
| AWPSO | 2.71364E+03 | 6.35890E+00 | 2.76722E+03 | 4.72402E+01 | 3.41072E+03 | 2.63358E+02 |
| PSO | 2.71022E+03 | 6.02059E+00 | 2.77043E+03 | 4.66198E+01 | 3.49742E+03 | 2.72439E+02 |
|  | F28 |  | F29 |  | F30 |  |
|  | AVG | STD | AVG | STD | AVG | STD |
| SRWPSO | **3.00000E+03** | **0.00000E+00** | **3.10000E+03** | **0.00000E+00** | **3.20000E+03** | **0.00000E+00** |
| SRPSO | 3.00000E+03 | 0.00000E+00 | 3.10000E+03 | 0.00000E+00 | 3.20000E+03 | 0.00000E+00 |
| SWPSO | 3.00000E+03 | 0.00000E+00 | 3.10000E+03 | 0.00000E+00 | 3.20000E+03 | 0.00000E+00 |
| RWPSO | 4.51455E+03 | 4.41088E+02 | 7.67733E+05 | 2.86548E+06 | 8.61043E+03 | 1.03280E+04 |
| SOBPSO | 3.00000E+03 | 0.00000E+00 | 3.10000E+03 | 0.00000E+00 | 3.20000E+03 | 0.00000E+00 |
| RRPSO | 4.76388E+03 | 5.48157E+02 | 3.69698E+05 | 1.71525E+06 | 1.26448E+04 | 4.39363E+03 |
| AWPSO | 6.14887E+03 | 6.55867E+02 | 1.25392E+07 | 2.18568E+07 | 6.10171E+03 | 2.30096E+03 |
| PSO | 7.02563E+03 | 9.74875E+02 | 6.33650E+04 | 1.02966E+05 | 1.55121E+04 | 6.46560E+03 |

**Table 2.** The p-value of the comparison result of different versions

|  | F1 | F2 | F3 | F4 | F5 |
| --- | --- | --- | --- | --- | --- |
| SRPSO | **1.73440E-06** | **1.73440E-06** | **1.73440E-06** | **1.92092E-06** | **1.73440E-06** |
| SWPSO | **4.07151E-05** | **5.75165E-06** | **1.19734E-03** | **1.35948E-04** | **1.73440E-06** |
| RWPSO | 9.77722E-02 | 9.91795E-01 | 3.93334E-01 | 3.60039E-01 | 1.41390E-01 |
| SOBPSO | **1.73440E-06** | **1.73440E-06** | **1.73440E-06** | **1.73440E-06** | **1.73440E-06** |
| RRPSO | **1.73440E-06** | **1.73440E-06** | **1.73440E-06** | **1.73440E-06** | **1.73440E-06** |
| AWPSO | **3.51524E-06** | **1.63945E-05** | **1.28663E-03** | **3.06500E-04** | **1.73440E-06** |
| PSO | **1.73440E-06** | **1.73440E-06** | **1.73440E-06** | 6.14315E-01 | **1.73440E-06** |
|  | F6 | F7 | F8 | F9 | F10 |
| SRPSO | **1.47728E-04** | **1.73440E-06** | **2.87860E-06** | **7.69086E-06** | **1.36011E-05** |
| SWPSO | **4.53356E-04** | **4.74250E-03** | **2.12664E-06** | **1.02463E-05** | **2.35342E-06** |
| RWPSO | 5.57743E-01 | 8.18393E-02 | 2.53594E-01 | **5.21649E-06** | 3.93334E-01 |
| SOBPSO | **1.25057E-04** | **1.73440E-06** | **1.73440E-06** | **1.73440E-06** | **1.73440E-06** |
| RRPSO | **6.03501E-03** | **1.73440E-06** | **6.33914E-06** | 9.77722E-02 | **1.79885E-05** |
| AWPSO | **3.32689E-02** | **1.93954E-03** | **1.73440E-06** | 2.36936E-01 | **1.73440E-06** |
| PSO | **3.11232E-05** | **1.73440E-06** | **1.73440E-06** | **1.73440E-06** | **1.73440E-06** |
|  | F11 | F12 | F13 | F14 | F15 |
| SRNPSO | **2.35342E-06** | **1.73440E-06** | 5.30440E-01 | 1.15608E-01 | **1.73440E-06** |
| SWEPSO | **1.97295E-05** | 1.10926E-01 | **3.85424E-03** | 1.65027E-01 | 3.93334E-01 |
| RWPSO | 2.13358E-01 | 8.29013E-01 | 9.26255E-01 | 1.91522E-01 | 5.85712E-01 |
| SOBPSO | **1.73440E-06** | **1.73440E-06** | **2.18267E-02** | 7.65519E-01 | **1.73440E-06** |
| RNDPSO | **1.73440E-06** | **1.73440E-06** | 9.75387E-01 | **2.70292E-02** | **1.92092E-06** |
| WEGPSO | **4.68184E-03** | **1.85190E-02** | **7.71217E-04** | **1.65655E-02** | 7.65519E-01 |
| PSO | **1.73440E-06** | **1.73440E-06** | 5.71646E-01 | 5.57743E-01 | **1.73440E-06** |
|  | F16 | F17 | F18 | F19 | F20 |
| SRPSO | **1.31942E-02** | **1.73440E-06** | **1.73440E-06** | **1.56585E-02** | 3.60039E-01 |
| SWPSO | **1.97295E-05** | 1.20445E-01 | **4.38962E-03** | 7.65519E-01 | 1.41390E-01 |
| RWPSO | **4.49189E-02** | **1.70877E-03** | 3.38856E-01 | 7.65519E-01 | **1.60464E-04** |
| SOBPSO | **3.88218E-06** | **1.73440E-06** | **1.73440E-06** | **2.84860E-02** | **1.83258E-03** |
| RRPSO | **9.27103E-03** | **1.73440E-06** | **1.73440E-06** | **4.94980E-02** | 9.91795E-01 |
| AWPSO | **1.36011E-05** | **2.41180E-04** | 3.82034E-01 | 5.44625E-02 | 9.26255E-01 |
| PSO | **2.60333E-06** | **1.73440E-06** | **1.73440E-06** | **2.95746E-03** | **3.72426E-05** |
|  | F21 | F22 | F23 | F24 | F25 |
| SRPSO | **1.73440E-06** | 1.02011E-01 | 1.00000E+00 | 1.00000E+00 | 1.00000E+00 |
| SWPSO | 4.04835E-01 | **1.28663E-03** | 1.00000E+00 | 1.00000E+00 | 1.00000E+00 |
| RWPSO | 5.44625E-02 | 2.53644E-01 | **1.97730E-07** | **1.73440E-06** | **1.73440E-06** |
| SOBPSO | **4.72920E-06** | **3.31726E-04** | 1.00000E+00 | 1.00000E+00 | 1.00000E+00 |
| RRPSO | **5.21649E-06** | 7.65519E-01 | **1.73440E-06** | **1.73440E-06** | **1.73440E-06** |
| AWPSO | **1.10792E-02** | **8.72967E-03** | **7.00782E-07** | **1.73440E-06** | **1.73440E-06** |
| PSO | **4.28569E-06** | **3.58884E-04** | **1.73440E-06** | **1.73440E-06** | **1.73440E-06** |
|  | F26 | F27 | F28 | F29 | F30 |
| SRPSO | 7.18888E-01 | 1.00000E+00 | 1.00000E+00 | 1.00000E+00 | 1.00000E+00 |
| SWPSO | **1.96458E-03** | 1.00000E+00 | 1.00000E+00 | 1.00000E+00 | 1.00000E+00 |
| RWPSO | **3.50090E-02** | **1.73440E-06** | **1.73440E-06** | **1.73440E-06** | **1.73440E-06** |
| SOBPSO | 4.16534E-01 | 1.00000E+00 | 1.00000E+00 | 1.00000E+00 | 1.00000E+00 |
| RRPSO | **4.19551E-04** | **1.73440E-06** | **1.73440E-06** | **1.73440E-06** | **1.73440E-06** |
| AWPSO | **1.73440E-06** | **1.73440E-06** | **1.73440E-06** | **1.73440E-06** | **1.73440E-06** |
| PSO | **4.28569E-06** | **1.73440E-06** | **1.73440E-06** | **1.73440E-06** | **1.73440E-06** |

**Table 3.** Comparison results of SRWPSO with traditional algorithms

|  | F1 |  | F2 |  | F3 |  |
| --- | --- | --- | --- | --- | --- | --- |
|  | AVG | STD | AVG | STD | AVG | STD |
| SRWPSO | **3.58922E+05** | 1.24528E+06 | **2.17495E+02** | **2.07568E+01** | **3.00233E+02** | **5.64776E-01** |
| ACOR | 5.33558E+06 | 7.77071E+06 | 1.41808E+07 | 7.76146E+07 | 8.03497E+03 | 1.10131E+04 |
| DE | 2.03966E+07 | 4.80935E+06 | 4.48211E+02 | 9.65082E+02 | 3.88166E+02 | 8.79677E+01 |
| SCA | 2.42427E+08 | 6.80501E+07 | 1.54583E+10 | 2.92608E+09 | 3.96275E+04 | 7.69182E+03 |
| HHO | 1.13386E+07 | 6.01272E+06 | 1.14404E+07 | 2.48710E+06 | 5.26734E+03 | 1.70407E+03 |
| GWO | 7.30729E+07 | 5.95060E+07 | 1.93762E+09 | 2.12935E+09 | 3.27731E+04 | 7.12935E+03 |
| WOA | 3.29853E+07 | 1.47615E+07 | 4.27760E+06 | 3.85066E+06 | 3.86510E+04 | 2.10211E+04 |
| BA | 7.97898E+05 | **5.88910E+05** | 5.57483E+05 | 3.07928E+05 | 4.44446E+02 | 2.81502E+02 |
| MFO | 8.17998E+07 | 8.07694E+07 | 1.31189E+10 | 7.90204E+09 | 9.16896E+04 | 5.71575E+04 |
| WDO | 1.46210E+07 | 4.14527E+06 | 6.15037E+07 | 1.40340E+07 | 3.02376E+03 | 7.78956E+02 |
|  | F4 |  | F5 |  | F6 |  |
|  | AVG | STD | AVG | STD | AVG | STD |
| SRWPSO | 4.60959E+02 | 3.83241E+01 | **5.20007E+02** | **3.95486E-02** | 6.17251E+02 | 2.61463E+00 |
| ACOR | 4.97730E+02 | 1.00829E+02 | 5.20902E+02 | 7.16684E-02 | **6.12491E+02** | 2.75092E+00 |
| DE | 5.00851E+02 | **2.85514E+01** | 5.20562E+02 | 5.65441E-02 | 6.18881E+02 | **2.26495E+00** |
| SCA | 1.35477E+03 | 2.28295E+02 | 5.20922E+02 | 5.74888E-02 | 6.32480E+02 | 2.83563E+00 |
| HHO | 5.45498E+02 | 5.22539E+01 | 5.20243E+02 | 1.73655E-01 | 6.31014E+02 | 4.04744E+00 |
| GWO | 6.64706E+02 | 9.97722E+01 | 5.20934E+02 | 7.40426E-02 | 6.13555E+02 | 2.45978E+00 |
| WOA | 5.96257E+02 | 5.13158E+01 | 5.20400E+02 | 1.97578E-01 | 6.35384E+02 | 3.62484E+00 |
| BA | **4.40741E+02** | 3.98315E+01 | 5.20958E+02 | 6.24940E-02 | 6.34891E+02 | 3.43496E+00 |
| MFO | 1.23809E+03 | 8.17603E+02 | 5.20271E+02 | 1.81091E-01 | 6.23716E+02 | 3.55767E+00 |
| WDO | 6.00367E+02 | 4.86197E+01 | 5.20797E+02 | 1.68036E-01 | 6.33211E+02 | 3.49259E+00 |
|  | F7 |  | F8 |  | F9 |  |
|  | AVG | STD | AVG | STD | AVG | STD |
| SRWPSO | 7.00013E+02 | 1.25759E-02 | 8.66297E+02 | 1.03150E+01 | 1.03367E+03 | 2.12666E+01 |
| ACOR | 7.04763E+02 | 8.59489E+00 | 8.66637E+02 | 2.11270E+01 | 1.01869E+03 | 6.57038E+01 |
| DE | **7.00000E+02** | **2.00121E-10** | **8.00942E+02** | **1.35838E+00** | 1.01182E+03 | **8.35684E+00** |
| SCA | 8.39290E+02 | 3.35324E+01 | 1.04017E+03 | 1.86668E+01 | 1.17349E+03 | 1.86329E+01 |
| HHO | 7.01110E+02 | 2.00125E-02 | 8.98128E+02 | 1.48351E+01 | 1.08985E+03 | 2.38978E+01 |
| GWO | 7.25784E+02 | 2.25456E+01 | 8.82923E+02 | 2.01603E+01 | **1.00443E+03** | 2.27145E+01 |
| WOA | 7.01021E+02 | 7.02435E-02 | 9.86473E+02 | 2.87043E+01 | 1.13602E+03 | 5.17591E+01 |
| BA | 7.00646E+02 | 2.09126E-01 | 1.01245E+03 | 4.10191E+01 | 1.17568E+03 | 7.16581E+01 |
| MFO | 8.02744E+02 | 7.60048E+01 | 9.47905E+02 | 4.40687E+01 | 1.11729E+03 | 4.91172E+01 |
| WDO | 7.10785E+02 | 1.54585E+00 | 9.57945E+02 | 2.62325E+01 | 1.07707E+03 | 2.85110E+01 |
|  | F10 |  | F11 |  | F12 |  |
|  | AVG | STD | AVG | STD | AVG | STD |
| SRWPSO | 2.53560E+03 | 5.03786E+02 | **3.51067E+03** | 4.57122E+02 | **1.20020E+03** | **6.60491E-02** |
| ACOR | 3.17091E+03 | 6.16431E+02 | 4.21453E+03 | 1.76007E+03 | 1.20240E+03 | 2.79450E-01 |
| DE | **1.02594E+03** | **3.00425E+01** | 5.66789E+03 | 3.44071E+02 | 1.20090E+03 | 1.18890E-01 |
| SCA | 7.07054E+03 | 4.13163E+02 | 8.13096E+03 | **2.30325E+02** | 1.20245E+03 | 2.97022E-01 |
| HHO | 2.45502E+03 | 6.09396E+02 | 5.36629E+03 | 5.64842E+02 | 1.20146E+03 | 4.27582E-01 |
| GWO | 3.05187E+03 | 4.92672E+02 | 3.91321E+03 | 5.51712E+02 | 1.20104E+03 | 1.15059E+00 |
| WOA | 5.18048E+03 | 8.31640E+02 | 5.86822E+03 | 8.08428E+02 | 1.20165E+03 | 4.64904E-01 |
| BA | 5.52710E+03 | 6.57389E+02 | 5.58052E+03 | 6.19008E+02 | 1.20121E+03 | 3.32699E-01 |
| MFO | 4.49069E+03 | 9.34315E+02 | 5.63572E+03 | 7.62035E+02 | 1.20044E+03 | 2.54969E-01 |
| WDO | 5.22084E+03 | 7.61707E+02 | 5.79340E+03 | 9.49623E+02 | 1.20132E+03 | 3.94642E-01 |
|  | F13 |  | F14 |  | F15 |  |
|  | AVG | STD | AVG | STD | AVG | STD |
| SRWPSO | **1.30033E+03** | 7.23533E-02 | **1.40026E+03** | 4.94228E-02 | **1.50632E+03** | 2.20419E+00 |
| ACOR | 1.30049E+03 | 1.45454E-01 | 1.40072E+03 | 2.65866E-01 | 9.94940E+03 | 4.45291E+04 |
| DE | 1.30035E+03 | **3.35108E-02** | 1.40034E+03 | 9.45405E-02 | 1.51175E+03 | **7.87828E-01** |
| SCA | 1.30305E+03 | 2.89806E-01 | 1.44531E+03 | 1.26080E+01 | 3.59637E+03 | 1.62923E+03 |
| HHO | 1.30052E+03 | 1.08938E-01 | 1.40029E+03 | 1.12906E-01 | 1.53744E+03 | 6.70259E+00 |
| GWO | 1.30039E+03 | 7.75770E-02 | 1.40489E+03 | 6.29138E+00 | 1.55756E+03 | 5.57031E+01 |
| WOA | 1.30051E+03 | 1.13670E-01 | 1.40027E+03 | **4.55216E-02** | 1.57658E+03 | 2.20711E+01 |
| BA | 1.30049E+03 | 1.09421E-01 | 1.40036E+03 | 1.36856E-01 | 1.52732E+03 | 5.84238E+00 |
| MFO | 1.30155E+03 | 1.30732E+00 | 1.43416E+03 | 2.85719E+01 | 1.63862E+05 | 3.00183E+05 |
| WDO | 1.30046E+03 | 5.08346E-02 | 1.40035E+03 | 8.23515E-02 | 1.53121E+03 | 5.66660E+00 |
|  | F16 |  | F17 |  | F18 |  |
|  | AVG | STD | AVG | STD | AVG | STD |
| SRWPSO | **1.61057E+03** | 6.12262E-01 | **2.69806E+04** | **9.93999E+03** | 6.00743E+03 | 4.83755E+03 |
| ACOR | 1.61151E+03 | 3.88915E-01 | 1.71990E+05 | 3.14952E+05 | 1.09525E+04 | 8.40293E+03 |
| DE | 1.61142E+03 | 3.22648E-01 | 1.39787E+06 | 6.40354E+05 | 8.64847E+03 | 5.48196E+03 |
| SCA | 1.61282E+03 | 2.96049E-01 | 6.85362E+06 | 3.20797E+06 | 1.44049E+08 | 7.55055E+07 |
| HHO | 1.61233E+03 | 3.75168E-01 | 1.53613E+06 | 1.07085E+06 | 8.98053E+04 | 4.48250E+04 |
| GWO | 1.61108E+03 | 7.34371E-01 | 1.77913E+06 | 2.38098E+06 | 6.77150E+06 | 1.73253E+07 |
| WOA | 1.61249E+03 | 4.35721E-01 | 3.47899E+06 | 1.96464E+06 | **4.92623E+03** | **3.04922E+03** |
| BA | 1.61328E+03 | **2.75036E-01** | 1.01600E+05 | 6.06603E+04 | 9.76187E+04 | 4.73560E+04 |
| MFO | 1.61279E+03 | 5.76347E-01 | 3.44860E+06 | 4.78989E+06 | 2.87586E+05 | 5.00001E+05 |
| WDO | 1.61296E+03 | 4.39599E-01 | 3.52696E+05 | 1.14037E+05 | 1.72975E+05 | 5.39984E+04 |
|  | F19 |  | F20 |  | F21 |  |
|  | AVG | STD | AVG | STD | AVG | STD |
| SRWPSO | 1.91709E+03 | 1.41610E+01 | **2.23241E+03** | 1.15619E+02 | **3.68522E+04** | 4.73716E+04 |
| ACOR | 1.92130E+03 | 2.37303E+01 | 1.44411E+04 | 2.33899E+04 | 8.03559E+04 | 8.39276E+04 |
| DE | **1.90824E+03** | **6.34655E-01** | 5.22798E+03 | 1.45746E+03 | 3.01914E+05 | 1.43297E+05 |
| SCA | 1.98445E+03 | 1.91253E+01 | 1.56809E+04 | 4.45029E+03 | 1.25821E+06 | 5.53411E+05 |
| HHO | 1.93985E+03 | 4.01231E+01 | 1.27334E+04 | 6.00492E+03 | 4.11325E+05 | 4.95140E+05 |
| GWO | 1.94922E+03 | 2.81152E+01 | 1.69688E+04 | 7.41365E+03 | 3.99839E+05 | 5.49433E+05 |
| WOA | 1.94380E+03 | 3.13673E+01 | 3.42020E+04 | 2.57168E+04 | 1.66530E+06 | 2.37286E+06 |
| BA | 1.91965E+03 | 1.50957E+01 | 2.35611E+03 | **1.05870E+02** | 4.98618E+04 | **2.32684E+04** |
| MFO | 1.97430E+03 | 6.81306E+01 | 6.24237E+04 | 4.32536E+04 | 6.50861E+05 | 5.97832E+05 |
| WDO | 1.94952E+03 | 3.45028E+01 | 2.90271E+03 | 3.61313E+02 | 1.34123E+05 | 4.78877E+04 |
|  | F22 |  | F23 |  | F24 |  |
|  | AVG | STD | AVG | STD | AVG | STD |
| SRWPSO | 2.68859E+03 | 1.84691E+02 | **2.50000E+03** | **0.00000E+00** | **2.60000E+03** | **0.00000E+00** |
| ACOR | 2.53586E+03 | 1.65853E+02 | 2.62002E+03 | 8.08707E+00 | 2.64492E+03 | 1.11073E+01 |
| DE | **2.34860E+03** | **7.24537E+01** | 2.61524E+03 | 1.38756E-12 | 2.62518E+03 | 1.78869E+00 |
| SCA | 2.91931E+03 | 1.47666E+02 | 2.66934E+03 | 1.27134E+01 | 2.60005E+03 | 3.41107E-02 |
| HHO | 3.07873E+03 | 2.55242E+02 | 2.50000E+03 | 0.00000E+00 | 2.60000E+03 | 5.32323E-05 |
| GWO | 2.59185E+03 | 1.52532E+02 | 2.63459E+03 | 9.46867E+00 | 2.60000E+03 | 9.23398E-04 |
| WOA | 2.98338E+03 | 2.90035E+02 | 2.63343E+03 | 7.42790E+00 | 2.60491E+03 | 3.75258E+00 |
| BA | 3.31700E+03 | 2.45317E+02 | 2.61525E+03 | 2.75904E-03 | 2.66776E+03 | 2.29260E+01 |
| MFO | 3.02553E+03 | 3.00918E+02 | 2.66222E+03 | 3.00334E+01 | 2.67337E+03 | 3.02204E+01 |
| WDO | 3.09623E+03 | 2.88626E+02 | 2.59732E+03 | 4.94938E+01 | 2.60000E+03 | 6.37132E-06 |
|  | F25 |  | F26 |  | F27 |  |
|  | AVG | STD | AVG | STD | AVG | STD |
| SRWPSO | **2.70000E+03** | **0.00000E+00** | 2.70034E+03 | 9.44470E-02 | **2.90000E+03** | **0.00000E+00** |
| ACOR | 2.70714E+03 | 2.17180E+00 | 2.72302E+03 | 6.87284E+01 | 3.45509E+03 | 7.48668E+01 |
| DE | 2.70707E+03 | 1.33738E+00 | **2.70033E+03** | **3.35802E-02** | 3.22598E+03 | 8.51261E+01 |
| SCA | 2.72479E+03 | 7.80339E+00 | 2.70261E+03 | 4.86868E-01 | 3.46386E+03 | 3.08847E+02 |
| HHO | 2.70000E+03 | 0.00000E+00 | 2.79336E+03 | 2.52535E+01 | 2.90000E+03 | 0.00000E+00 |
| GWO | 2.71112E+03 | 5.05338E+00 | 2.76417E+03 | 5.70898E+01 | 3.34036E+03 | 1.11105E+02 |
| WOA | 2.71047E+03 | 1.32860E+01 | 2.70375E+03 | 1.81823E+01 | 3.80239E+03 | 3.64378E+02 |
| BA | 2.73287E+03 | 1.38786E+01 | 2.70052E+03 | 1.60820E-01 | 3.84330E+03 | 3.86973E+02 |
| MFO | 2.71801E+03 | 1.13425E+01 | 2.70255E+03 | 1.37105E+00 | 3.60866E+03 | 1.76187E+02 |
| WDO | 2.70000E+03 | 0.00000E+00 | 2.78347E+03 | 3.76054E+01 | 3.56818E+03 | 4.26863E+02 |
|  | F28 |  | F29 |  | F30 |  |
|  | AVG | STD | AVG | STD | AVG | STD |
| SRWPSO | **3.00000E+03** | **0.00000E+00** | **3.10000E+03** | **0.00000E+00** | **3.20000E+03** | **0.00000E+00** |
| ACOR | 3.89890E+03 | 2.47350E+02 | 2.43713E+06 | 4.11393E+06 | 1.41252E+04 | 1.88788E+04 |
| DE | 3.63822E+03 | 2.59610E+01 | 4.80669E+03 | 8.18645E+02 | 6.17384E+03 | 1.19946E+03 |
| SCA | 4.85394E+03 | 3.31821E+02 | 1.20361E+07 | 8.04559E+06 | 2.70078E+05 | 1.09201E+05 |
| HHO | 3.00000E+03 | 0.00000E+00 | 3.78700E+03 | 3.76283E+03 | 6.06550E+03 | 7.47168E+03 |
| GWO | 3.85655E+03 | 1.79728E+02 | 1.60151E+06 | 3.18054E+06 | 5.77170E+04 | 6.09380E+04 |
| WOA | 5.03698E+03 | 7.30249E+02 | 6.55851E+06 | 4.74630E+06 | 1.04107E+05 | 5.75596E+04 |
| BA | 5.37376E+03 | 9.62380E+02 | 4.37520E+07 | 4.92533E+07 | 2.37478E+04 | 5.68125E+04 |
| MFO | 3.98840E+03 | 2.27289E+02 | 4.23913E+06 | 4.00413E+06 | 4.86687E+04 | 4.08872E+04 |
| WDO | 8.59318E+03 | 1.05307E+03 | 4.85187E+04 | 2.60342E+04 | 2.43267E+04 | 4.41207E+03 |

**Table 4.** The p-value of the comparison result of SRWPSO with traditional algorithms

|  | F1 | F2 | F3 | F4 | F5 |
| --- | --- | --- | --- | --- | --- |
| ACOR | **1.73440E-06** | **1.92092E-06** | **1.73440E-06** | 1.30592E-01 | **1.73440E-06** |
| DE | **1.73440E-06** | 2.05888E-01 | **1.73440E-06** | **3.31726E-04** | **1.73440E-06** |
| SCA | **1.73440E-06** | **1.73440E-06** | **1.73440E-06** | **1.73440E-06** | **1.73440E-06** |
| HHO | **1.92092E-06** | **1.73440E-06** | **1.73440E-06** | **4.72920E-06** | **1.73440E-06** |
| GWO | **1.73440E-06** | **1.73440E-06** | **1.73440E-06** | **1.73440E-06** | **1.73440E-06** |
| WOA | **1.73440E-06** | **1.73440E-06** | **1.73440E-06** | **1.73440E-06** | **1.73440E-06** |
| BA | **3.40526E-05** | **1.73440E-06** | **1.73440E-06** | **1.47954E-02** | **1.73440E-06** |
| MFO | **1.73440E-06** | **1.73440E-06** | **1.73440E-06** | **1.73440E-06** | **2.35342E-06** |
| WDO | **1.73440E-06** | **1.73440E-06** | **1.73440E-06** | **1.73440E-06** | **1.73440E-06** |
|  | F6 | F7 | F8 | F9 | F10 |
| ACOR | **8.46608E-06** | 1.86544E-01 | 6.28843E-01 | 2.28880E-01 | **3.31726E-04** |
| DE | **3.68261E-02** | **5.47240E-06** | **1.73440E-06** | **8.18775E-05** | **1.73440E-06** |
| SCA | **1.73440E-06** | **1.73440E-06** | **1.73440E-06** | **1.73440E-06** | **1.73440E-06** |
| HHO | **1.73440E-06** | **1.73440E-06** | **1.73440E-06** | **1.73440E-06** | 3.28571E-01 |
| GWO | **1.47728E-04** | **1.73440E-06** | **3.06500E-04** | **8.18775E-05** | **1.48393E-03** |
| WOA | **1.73440E-06** | **1.73440E-06** | **1.73440E-06** | **1.73440E-06** | **1.73440E-06** |
| BA | **1.73440E-06** | **1.73440E-06** | **1.73440E-06** | **1.73440E-06** | **1.73440E-06** |
| MFO | **6.98378E-06** | **1.73440E-06** | **1.73440E-06** | **1.73440E-06** | **1.73440E-06** |
| WDO | **1.73440E-06** | **1.73440E-06** | **1.73440E-06** | **1.49356E-05** | **1.73440E-06** |
|  | F11 | F12 | F13 | F14 | F15 |
| ACOR | 2.98944E-01 | **1.73440E-06** | **3.11232E-05** | **4.72920E-06** | **1.73440E-06** |
| DE | **1.73440E-06** | **1.73440E-06** | 1.91522E-01 | **2.59671E-05** | **1.92092E-06** |
| SCA | **1.73440E-06** | **1.73440E-06** | **1.73440E-06** | **1.73440E-06** | **1.73440E-06** |
| HHO | **2.12664E-06** | **1.73440E-06** | **5.21649E-06** | 3.93334E-01 | **1.73440E-06** |
| GWO | **1.39746E-02** | 6.56411E-02 | **2.06711E-02** | **4.44934E-05** | **1.92092E-06** |
| WOA | **2.35342E-06** | **1.73440E-06** | **7.69086E-06** | 4.28430E-01 | **1.73440E-06** |
| BA | **1.73440E-06** | **1.73440E-06** | **1.92092E-06** | **1.03568E-03** | **1.73440E-06** |
| MFO | **1.73440E-06** | **4.72920E-06** | **1.73440E-06** | **1.73440E-06** | **1.73440E-06** |
| WDO | **1.73440E-06** | **1.73440E-06** | **9.31566E-06** | **2.37045E-05** | **1.73440E-06** |
|  | F16 | F17 | F18 | F19 | F20 |
| ACOR | **6.33914E-06** | **8.91873E-05** | **1.31942E-02** | 1.84622E-01 | **1.73440E-06** |
| DE | **4.28569E-06** | **1.73440E-06** | 8.58958E-02 | **1.73440E-06** | **1.73440E-06** |
| SCA | **1.73440E-06** | **1.73440E-06** | **1.73440E-06** | **1.92092E-06** | **1.73440E-06** |
| HHO | **1.73440E-06** | **1.73440E-06** | **1.92092E-06** | **4.89690E-04** | **1.73440E-06** |
| GWO | **9.27103E-03** | **1.73440E-06** | **8.21674E-03** | **1.23808E-05** | **1.73440E-06** |
| WOA | **1.73440E-06** | **1.73440E-06** | 7.65519E-01 | **1.49356E-05** | **1.73440E-06** |
| BA | **1.73440E-06** | **2.35342E-06** | **1.73440E-06** | **1.04444E-02** | **7.71217E-04** |
| MFO | **1.73440E-06** | **2.35342E-06** | **1.02463E-05** | **1.79885E-05** | **1.73440E-06** |
| WDO | **1.73440E-06** | **1.73440E-06** | **1.73440E-06** | **1.03568E-03** | **1.73440E-06** |
|  | F21 | F22 | F23 | F24 | F25 |
| ACOR | **1.39746E-02** | **1.38204E-03** | **1.44016E-06** | **1.73440E-06** | **1.73440E-06** |
| DE | **1.73440E-06** | **2.35342E-06** | **4.32046E-08** | **1.73440E-06** | **1.73440E-06** |
| SCA | **1.73440E-06** | **3.58884E-04** | **1.73440E-06** | **1.73440E-06** | **2.56308E-06** |
| HHO | **4.28569E-06** | **8.46608E-06** | 1.00000E+00 | **2.70159E-05** | 1.00000E+00 |
| GWO | **1.23808E-05** | **3.32689E-02** | **1.73440E-06** | **1.73440E-06** | **8.29810E-06** |
| WOA | **1.73440E-06** | **4.89690E-04** | **1.73440E-06** | **1.73440E-06** | **1.22070E-04** |
| BA | **4.99155E-03** | **1.92092E-06** | **1.73440E-06** | **1.73440E-06** | **1.73440E-06** |
| MFO | **3.88218E-06** | **3.06500E-04** | **1.73440E-06** | **1.73440E-06** | **1.73440E-06** |
| WDO | **3.88218E-06** | **1.12654E-05** | **1.82153E-05** | **1.73440E-06** | 1.00000E+00 |
|  | F26 | F27 | F28 | F29 | F30 |
| ACOR | **1.63945E-05** | **1.73440E-06** | **1.73440E-06** | **1.73440E-06** | **1.73440E-06** |
| DE | 7.97098E-01 | **1.73440E-06** | **1.73440E-06** | **1.73440E-06** | **1.73440E-06** |
| SCA | **1.73440E-06** | **1.73440E-06** | **1.73440E-06** | **1.73440E-06** | **1.73440E-06** |
| HHO | **2.12664E-06** | 1.00000E+00 | 1.00000E+00 | 1.00000E+00 | 6.25000E-02 |
| GWO | **2.87860E-06** | **1.73440E-06** | **1.73440E-06** | **1.73440E-06** | **1.73440E-06** |
| WOA | **1.38204E-03** | **1.73440E-06** | **2.56308E-06** | **1.73004E-06** | **1.73440E-06** |
| BA | **3.72426E-05** | **1.73440E-06** | **1.73440E-06** | **1.73440E-06** | **1.73440E-06** |
| MFO | **1.73440E-06** | **1.73440E-06** | **1.73440E-06** | **1.73004E-06** | **1.73440E-06** |
| WDO | **1.73440E-06** | **1.73440E-06** | **1.73440E-06** | **8.29810E-06** | **1.73440E-06** |

**Table 5.** Comparison results of SRWPSO with famous variants

|  | F1 |  | F2 |  | F3 |  |
| --- | --- | --- | --- | --- | --- | --- |
|  | AVG | STD | AVG | STD | AVG | STD |
| SRWPSO | **2.02549E+05** | **2.65156E+05** | **2.25424E+02** | **2.64665E+01** | **3.00346E+02** | **9.17776E-01** |
| SCADE | 4.32400E+08 | 1.00994E+08 | 2.97952E+10 | 2.99154E+09 | 5.42038E+04 | 5.72681E+03 |
| CBA | 4.40583E+06 | 1.42665E+06 | 9.25459E+03 | 7.98505E+03 | 4.34845E+03 | 4.08069E+03 |
| RCACO | 8.63447E+05 | 1.13447E+06 | 1.61554E+04 | 1.38469E+04 | 3.91895E+03 | 2.90920E+03 |
| m_SCA | 6.60811E+07 | 3.82892E+07 | 7.73651E+09 | 3.61141E+09 | 2.76908E+04 | 6.19577E+03 |
| CLACO | 2.17254E+06 | 1.94053E+06 | 1.12743E+04 | 1.06137E+04 | 9.85308E+02 | 9.79101E+02 |
| SCA_PSO | 8.40413E+06 | 2.96175E+06 | 3.64870E+07 | 9.65500E+06 | 2.95283E+03 | 1.25428E+03 |
| RDWOA | 8.77822E+06 | 4.87410E+06 | 1.89831E+07 | 2.06165E+07 | 7.24490E+03 | 3.41596E+03 |
| OBLGWO | 1.90749E+07 | 1.00759E+07 | 1.47739E+07 | 1.14712E+07 | 8.75926E+03 | 3.43592E+03 |
| FSTPSO | 3.80413E+08 | 1.70946E+08 | 2.95688E+10 | 8.68705E+09 | 8.98820E+04 | 2.70776E+04 |
|  | F4 |  | F5 |  | F6 |  |
|  | AVG | STD | AVG | STD | AVG | STD |
| SRWPSO | 4.62458E+02 | **2.51851E+01** | 5.20015E+02 | 6.16944E-02 | 6.18135E+02 | 3.17766E+00 |
| SCADE | 2.67545E+03 | 1.07152E+03 | 5.20956E+02 | 4.56254E-02 | 6.34648E+02 | **1.99351E+00** |
| CBA | 5.08370E+02 | 2.98953E+01 | 5.20151E+02 | 2.08228E-01 | 6.41835E+02 | 3.05420E+00 |
| RCACO | **4.16873E+02** | 3.31370E+01 | 5.20675E+02 | 7.40327E-02 | **6.08106E+02** | 2.50906E+00 |
| m_SCA | 7.70132E+02 | 9.13315E+01 | 5.20637E+02 | 1.47423E-01 | 6.22124E+02 | 3.21742E+00 |
| CLACO | 4.42458E+02 | 3.06452E+01 | **5.20000E+02** | **1.03877E-03** | 6.13363E+02 | 6.10236E+00 |
| SCA_PSO | 4.85299E+02 | 4.23604E+01 | 5.20947E+02 | 5.72511E-02 | 6.29370E+02 | 3.27641E+00 |
| RDWOA | 5.24657E+02 | 4.55915E+01 | 5.20127E+02 | 7.58449E-02 | 6.23643E+02 | 4.14603E+00 |
| OBLGWO | 5.37498E+02 | 3.32256E+01 | 5.20938E+02 | 4.71879E-02 | 6.19514E+02 | 5.12582E+00 |
| FSTPSO | 3.72867E+03 | 1.46349E+03 | 5.20550E+02 | 9.92451E-02 | 6.35517E+02 | 2.69349E+00 |
|  | F7 |  | F8 |  | F9 |  |
|  | AVG | STD | AVG | STD | AVG | STD |
| SRWPSO | 7.00011E+02 | 1.41998E-02 | 8.66728E+02 | 1.25362E+01 | 1.03961E+03 | 2.10829E+01 |
| SCADE | 9.01455E+02 | 3.14994E+01 | 1.06977E+03 | 1.42144E+01 | 1.20108E+03 | **1.71556E+01** |
| CBA | 7.00009E+02 | 1.15400E-02 | 1.01921E+03 | 3.97511E+01 | 1.16355E+03 | 7.02947E+01 |
| RCACO | **7.00004E+02** | **6.15789E-03** | 8.27798E+02 | 9.93700E+00 | **1.00868E+03** | 2.46266E+01 |
| m_SCA | 7.44891E+02 | 3.01304E+01 | 9.35019E+02 | 2.74839E+01 | 1.05168E+03 | 3.36274E+01 |
| CLACO | 7.00009E+02 | 1.56912E-02 | **8.07230E+02** | **5.03910E+00** | 1.01976E+03 | 3.36716E+01 |
| SCA_PSO | 7.01353E+02 | 8.32903E-02 | 9.90978E+02 | 3.84337E+01 | 1.13580E+03 | 4.75822E+01 |
| RDWOA | 7.00977E+02 | 2.43871E-01 | 8.47586E+02 | 1.28242E+01 | 1.07812E+03 | 5.23451E+01 |
| OBLGWO | 7.01150E+02 | 6.55224E-02 | 9.17018E+02 | 2.58525E+01 | 1.06776E+03 | 3.07891E+01 |
| FSTPSO | 9.81461E+02 | 5.65886E+01 | 1.03458E+03 | 3.15495E+01 | 1.16582E+03 | 3.56854E+01 |
|  | F10 |  | F11 |  | F12 |  |
|  | AVG | STD | AVG | STD | AVG | STD |
| SRWPSO | 2.42462E+03 | 4.49963E+02 | **3.64063E+03** | 4.63757E+02 | 1.20020E+03 | 5.42925E-02 |
| SCADE | 7.37887E+03 | 2.84143E+02 | 8.22936E+03 | **3.15153E+02** | 1.20263E+03 | 3.08821E-01 |
| CBA | 5.39063E+03 | 6.59042E+02 | 5.61006E+03 | 6.31540E+02 | 1.20108E+03 | 5.17133E-01 |
| RCACO | 1.89594E+03 | 2.78087E+02 | 5.78939E+03 | 7.59984E+02 | 1.20117E+03 | 2.87484E-01 |
| m_SCA | 4.20218E+03 | 5.79996E+02 | 4.76580E+03 | 7.80286E+02 | 1.20078E+03 | 3.05216E-01 |
| CLACO | **1.20131E+03** | **1.41007E+02** | 4.19909E+03 | 5.54817E+02 | **1.20015E+03** | **5.02740E-02** |
| SCA_PSO | 5.08391E+03 | 7.17781E+02 | 5.51187E+03 | 5.97805E+02 | 1.20212E+03 | 3.23808E-01 |
| RDWOA | 1.61845E+03 | 3.20189E+02 | 4.61036E+03 | 6.06046E+02 | 1.20047E+03 | 1.94818E-01 |
| OBLGWO | 3.97598E+03 | 8.22518E+02 | 5.06273E+03 | 7.82905E+02 | 1.20242E+03 | 6.14879E-01 |
| FSTPSO | 6.66706E+03 | 5.38416E+02 | 6.78080E+03 | 6.52211E+02 | 1.20156E+03 | 4.18014E-01 |
|  | F13 |  | F14 |  | F15 |  |
|  | AVG | STD | AVG | STD | AVG | STD |
| SRWPSO | **1.30035E+03** | 8.55092E-02 | 1.40027E+03 | **4.20255E-02** | **1.50658E+03** | 3.14645E+00 |
| SCADE | 1.30390E+03 | 3.46792E-01 | 1.48835E+03 | 1.41409E+01 | 1.85490E+04 | 8.99325E+03 |
| CBA | 1.30050E+03 | 1.27500E-01 | 1.40033E+03 | 1.09996E-01 | 1.55979E+03 | 1.47820E+01 |
| RCACO | 1.30037E+03 | **7.09227E-02** | 1.40043E+03 | 2.07019E-01 | 1.51374E+03 | 3.70978E+00 |
| m_SCA | 1.30112E+03 | 8.43072E-01 | 1.41751E+03 | 8.98881E+00 | 1.90577E+03 | 4.98399E+02 |
| CLACO | 1.30038E+03 | 8.77802E-02 | 1.40041E+03 | 2.15755E-01 | 1.51132E+03 | 3.29152E+00 |
| SCA_PSO | 1.30044E+03 | 8.73614E-02 | **1.40024E+03** | 5.64858E-02 | 1.51732E+03 | **1.80176E+00** |
| RDWOA | 1.30046E+03 | 1.08104E-01 | 1.40026E+03 | 9.95370E-02 | 1.51979E+03 | 5.96932E+00 |
| OBLGWO | 1.30055E+03 | 1.19063E-01 | 1.40038E+03 | 1.60872E-01 | 1.51600E+03 | 5.06729E+00 |
| FSTPSO | 1.30436E+03 | 5.26346E-01 | 1.50257E+03 | 2.87284E+01 | 3.76485E+04 | 3.68998E+04 |
|  | F16 |  | F17 |  | F18 |  |
|  | AVG | STD | AVG | STD | AVG | STD |
| SRWPSO | **1.61049E+03** | 7.12955E-01 | **3.03412E+04** | **2.19883E+04** | **6.62802E+03** | **4.48913E+03** |
| SCADE | 1.61269E+03 | **1.91836E-01** | 1.51294E+07 | 6.66351E+06 | 2.06499E+08 | 1.23735E+08 |
| CBA | 1.61341E+03 | 2.51219E-01 | 2.27193E+05 | 1.67903E+05 | 8.75569E+03 | 9.59446E+03 |
| RCACO | 1.61131E+03 | 5.25517E-01 | 1.54568E+05 | 1.38100E+05 | 8.40806E+03 | 7.17611E+03 |
| m_SCA | 1.61172E+03 | 5.56247E-01 | 1.96666E+06 | 1.86487E+06 | 2.99135E+07 | 5.16676E+07 |
| CLACO | 1.61131E+03 | 5.95604E-01 | 1.74632E+05 | 1.29737E+05 | 9.30404E+03 | 8.00253E+03 |
| SCA_PSO | 1.61249E+03 | 3.02470E-01 | 1.49254E+05 | 1.10603E+05 | 7.67264E+05 | 2.52969E+05 |
| RDWOA | 1.61161E+03 | 6.10042E-01 | 1.01272E+06 | 5.63098E+05 | 6.80174E+03 | 5.73917E+03 |
| OBLGWO | 1.61195E+03 | 7.01160E-01 | 1.52509E+06 | 1.01929E+06 | 5.75840E+04 | 1.03983E+05 |
| FSTPSO | 1.61288E+03 | 5.59966E-01 | 1.08334E+07 | 1.18065E+07 | 4.34069E+07 | 5.99173E+07 |
|  | F19 |  | F20 |  | F21 |  |
|  | AVG | STD | AVG | STD | AVG | STD |
| SRWPSO | 1.91912E+03 | 1.80388E+01 | **2.37319E+03** | 5.82034E+02 | **3.04548E+04** | 4.27505E+04 |
| SCADE | 2.01150E+03 | 1.13313E+01 | 3.03377E+04 | 1.32312E+04 | 2.34078E+06 | 1.08932E+06 |
| CBA | 1.92775E+03 | 2.65648E+01 | 3.15300E+03 | 1.14947E+03 | 1.05850E+05 | 5.51424E+04 |
| RCACO | 1.91482E+03 | 2.11067E+01 | 2.67878E+03 | 4.85142E+02 | 1.61030E+05 | 1.82863E+05 |
| m_SCA | 1.94658E+03 | 2.52477E+01 | 1.04279E+04 | 4.29833E+03 | 5.10107E+05 | 4.03365E+05 |
| CLACO | **1.90808E+03** | **2.11270E+00** | 3.37703E+03 | 3.27487E+03 | 1.01035E+05 | 1.17255E+05 |
| SCA_PSO | 1.91820E+03 | 3.32804E+00 | 2.44360E+03 | **1.97620E+02** | 6.82599E+04 | **3.57431E+04** |
| RDWOA | 1.92403E+03 | 2.60224E+01 | 7.12813E+03 | 3.53447E+03 | 4.91335E+05 | 4.53158E+05 |
| OBLGWO | 1.92350E+03 | 3.33724E+01 | 5.65570E+03 | 2.21955E+03 | 4.95830E+05 | 3.96870E+05 |
| FSTPSO | 2.05464E+03 | 4.92603E+01 | 4.77264E+04 | 2.66626E+04 | 1.75416E+06 | 1.74246E+06 |
|  | F22 |  | F23 |  | F24 |  |
|  | AVG | STD | AVG | STD | AVG | STD |
| SRWPSO | 2.71187E+03 | 2.30882E+02 | **2.50000E+03** | **0.00000E+00** | **2.60000E+03** | **0.00000E+00** |
| SCADE | 3.07608E+03 | 1.69314E+02 | 2.50000E+03 | 0.00000E+00 | 2.60000E+03 | 3.22714E-07 |
| CBA | 3.30993E+03 | 2.75510E+02 | 2.61586E+03 | 2.62842E-01 | 2.67405E+03 | 3.17806E+01 |
| RCACO | **2.45751E+03** | 1.99825E+02 | 2.50001E+03 | 1.58418E-02 | 2.60009E+03 | 4.63145E-02 |
| m_SCA | 2.55158E+03 | 1.87090E+02 | 2.63724E+03 | 7.76218E+00 | 2.60000E+03 | 7.78710E-04 |
| CLACO | 2.48664E+03 | **1.58810E+02** | 2.61437E+03 | 2.33589E-01 | 2.62080E+03 | 9.32748E+00 |
| SCA_PSO | 3.13989E+03 | 2.39053E+02 | 2.50000E+03 | 0.00000E+00 | 2.60000E+03 | 0.00000E+00 |
| RDWOA | 2.76292E+03 | 1.94390E+02 | 2.50000E+03 | 0.00000E+00 | 2.60000E+03 | 2.04690E-04 |
| OBLGWO | 2.73410E+03 | 1.67906E+02 | 2.61863E+03 | 2.05947E+00 | 2.60000E+03 | 0.00000E+00 |
| FSTPSO | 3.16252E+03 | 3.27867E+02 | 2.74222E+03 | 5.38973E+01 | 2.68877E+03 | 1.13946E+01 |
|  | F25 |  | F26 |  | F27 |  |
|  | AVG | STD | AVG | STD | AVG | STD |
| SRWPSO | **2.70000E+03** | **0.00000E+00** | 2.70037E+03 | 1.16104E-01 | **2.90000E+03** | **0.00000E+00** |
| SCADE | 2.70000E+03 | 0.00000E+00 | 2.70368E+03 | 4.31374E-01 | 3.24392E+03 | 2.25485E+02 |
| CBA | 2.73079E+03 | 1.09709E+01 | 2.71450E+03 | 6.06540E+01 | 4.03442E+03 | 4.38555E+02 |
| RCACO | 2.70000E+03 | 2.70072E-04 | **2.70033E+03** | **6.31698E-02** | 2.90000E+03 | 3.97353E-03 |
| m_SCA | 2.71261E+03 | 2.69165E+00 | 2.70086E+03 | 3.82051E-01 | 3.15387E+03 | 7.91069E+01 |
| CLACO | 2.70089E+03 | 3.16300E-01 | 2.70037E+03 | 8.65001E-02 | 3.37789E+03 | 1.64847E+02 |
| SCA_PSO | 2.70000E+03 | 0.00000E+00 | 2.78341E+03 | 3.77386E+01 | 2.90000E+03 | 0.00000E+00 |
| RDWOA | 2.70000E+03 | 0.00000E+00 | 2.70091E+03 | 1.16005E+00 | 2.98522E+03 | 2.60924E+02 |
| OBLGWO | 2.70000E+03 | 0.00000E+00 | 2.70050E+03 | 9.11050E-02 | 3.09804E+03 | 3.10824E+02 |
| FSTPSO | 2.73661E+03 | 1.07993E+01 | 2.78856E+03 | 3.78219E+01 | 3.92291E+03 | 3.77243E+02 |
|  | F28 |  | F29 |  | F30 |  |
|  | AVG | STD | AVG | STD | AVG | STD |
| SRWPSO | **3.00000E+03** | **0.00000E+00** | **3.10000E+03** | **0.00000E+00** | **3.20000E+03** | **0.00000E+00** |
| SCADE | 5.35503E+03 | 5.34128E+02 | 1.71743E+07 | 1.03962E+07 | 4.22600E+05 | 1.49965E+05 |
| CBA | 5.63245E+03 | 1.09942E+03 | 5.04863E+07 | 5.50010E+07 | 2.38343E+04 | 3.89770E+04 |
| RCACO | 3.00001E+03 | 6.71108E-03 | 1.24536E+06 | 3.22690E+06 | 8.37129E+03 | 6.99950E+03 |
| m_SCA | 3.92715E+03 | 1.40440E+02 | 2.01145E+06 | 4.33060E+06 | 5.54396E+04 | 3.85028E+04 |
| CLACO | 3.24869E+03 | 5.54639E+01 | 3.13939E+03 | 7.89800E+01 | 3.83331E+03 | 2.40762E+02 |
| SCA_PSO | 3.00000E+03 | 0.00000E+00 | 4.17121E+03 | 4.49746E+03 | 1.28158E+04 | 9.46894E+03 |
| RDWOA | 3.00000E+03 | 0.00000E+00 | 2.34155E+06 | 3.94478E+06 | 1.02112E+04 | 5.28622E+03 |
| OBLGWO | 3.62092E+03 | 5.06749E+02 | 5.16932E+06 | 4.27388E+06 | 1.74225E+04 | 8.64310E+03 |
| FSTPSO | 8.26615E+03 | 1.08990E+03 | 1.42882E+07 | 1.35249E+07 | 3.50999E+05 | 2.10780E+05 |

**Table 6.** The p-value of the comparison result of SRWPSO with famous variants

|  | F1 | F2 | F3 | F4 | F5 |
| --- | --- | --- | --- | --- | --- |
| SCADE | **1.73440E-06** | **1.73440E-06** | **1.73440E-06** | **1.73440E-06** | **1.73440E-06** |
| CBA | **1.73440E-06** | **1.73440E-06** | **1.73440E-06** | **2.12664E-06** | **4.86026E-05** |
| RCACO | **2.84342E-05** | **1.73440E-06** | **1.73440E-06** | **1.14992E-04** | **1.73440E-06** |
| m_SCA | **1.73440E-06** | **1.73440E-06** | **1.73440E-06** | **1.73440E-06** | **1.73440E-06** |
| CLACO | **1.73440E-06** | **1.73440E-06** | **1.73440E-06** | **9.84214E-03** | **9.27103E-03** |
| SCA_PSO | **1.73440E-06** | **1.73440E-06** | **1.73440E-06** | **1.56585E-02** | **1.73440E-06** |
| RDWOA | **1.73440E-06** | **1.73440E-06** | **1.73440E-06** | **2.16302E-05** | **2.59671E-05** |
| OBLGWO | **1.73440E-06** | **1.73440E-06** | **1.73440E-06** | **2.87860E-06** | **1.73440E-06** |
| FSTPSO | **1.73440E-06** | **1.73440E-06** | **1.73440E-06** | **1.73440E-06** | **1.73440E-06** |
|  | F6 | F7 | F8 | F9 | F10 |
| SCADE | **1.73440E-06** | **1.73440E-06** | **1.73440E-06** | **1.73440E-06** | **1.73440E-06** |
| CBA | **1.73440E-06** | 3.80603E-01 | **1.73440E-06** | **1.73440E-06** | **1.73440E-06** |
| RCACO | **1.73440E-06** | **3.52948E-03** | **1.73331E-06** | **4.86026E-05** | **1.14992E-04** |
| m_SCA | **6.89229E-05** | **1.73440E-06** | **1.73440E-06** | 1.71376E-01 | **2.12664E-06** |
| CLACO | **3.37885E-03** | 2.40802E-01 | **1.73331E-06** | **1.31942E-02** | **1.73440E-06** |
| SCA_PSO | **1.73440E-06** | **1.73440E-06** | **1.73440E-06** | **2.35342E-06** | **1.73440E-06** |
| RDWOA | **1.49356E-05** | **1.73440E-06** | **2.59671E-05** | **2.10526E-03** | **2.35342E-06** |
| OBLGWO | 2.98944E-01 | **1.73440E-06** | **1.73440E-06** | **3.31726E-04** | **1.92092E-06** |
| FSTPSO | **1.73440E-06** | **1.73440E-06** | **1.73440E-06** | **1.73440E-06** | **1.73440E-06** |
|  | F11 | F12 | F13 | F14 | F15 |
| SCADE | **1.73440E-06** | **1.73440E-06** | **1.73440E-06** | **1.73440E-06** | **1.73440E-06** |
| CBA | **1.73440E-06** | **1.73440E-06** | **1.14992E-04** | **8.30707E-04** | **1.73440E-06** |
| RCACO | **1.73440E-06** | **1.73440E-06** | 1.91522E-01 | **1.70877E-03** | **5.75165E-06** |
| m_SCA | **6.33914E-06** | **1.73440E-06** | **1.73440E-06** | **2.60333E-06** | **1.73440E-06** |
| CLACO | **1.14992E-04** | **2.10526E-03** | 1.52861E-01 | **1.31942E-02** | **7.51366E-05** |
| SCA_PSO | **1.73440E-06** | **1.73440E-06** | **1.03568E-03** | **9.84214E-03** | **1.73440E-06** |
| RDWOA | **5.21649E-06** | **1.73440E-06** | **3.88111E-04** | 3.08615E-01 | **2.60333E-06** |
| OBLGWO | **2.87860E-06** | **1.73440E-06** | **6.98378E-06** | **1.49356E-05** | **2.60333E-06** |
| FSTPSO | **1.73440E-06** | **1.73440E-06** | **1.73440E-06** | **1.73440E-06** | **1.73440E-06** |
|  | F16 | F17 | F18 | F19 | F20 |
| SCADE | **1.73440E-06** | **1.73440E-06** | **1.73440E-06** | **1.73440E-06** | **1.73440E-06** |
| CBA | **1.73440E-06** | **2.60333E-06** | 8.13017E-01 | **1.38204E-03** | **3.72426E-05** |
| RCACO | **8.46608E-06** | **5.79245E-05** | 5.17048E-01 | **2.41470E-03** | **1.05695E-04** |
| m_SCA | **1.73440E-06** | **1.73440E-06** | **1.92092E-06** | **3.31726E-04** | **1.73440E-06** |
| CLACO | **3.72426E-05** | **1.73440E-06** | 2.62299E-01 | **2.87860E-06** | **3.06500E-04** |
| SCA_PSO | **1.73440E-06** | **4.28569E-06** | **1.73440E-06** | **9.84214E-03** | **7.27105E-03** |
| RDWOA | **9.31566E-06** | **1.73440E-06** | 8.13017E-01 | 5.99936E-01 | **1.73440E-06** |
| OBLGWO | **1.97295E-05** | **1.73440E-06** | **3.18168E-06** | 7.03564E-01 | **6.33914E-06** |
| FSTPSO | **1.73440E-06** | **1.73440E-06** | **1.73440E-06** | **1.73440E-06** | **1.73440E-06** |
|  | F21 | F22 | F23 | F24 | F25 |
| SCADE | **1.73440E-06** | **1.02463E-05** | 1.00000E+00 | **1.56250E-02** | 1.00000E+00 |
| CBA | **2.84342E-05** | **3.88218E-06** | **1.73440E-06** | **1.73440E-06** | **1.73440E-06** |
| RCACO | **3.31726E-04** | **3.88111E-04** | **1.73440E-06** | **1.73440E-06** | **2.56308E-06** |
| m_SCA | **1.73440E-06** | **1.10792E-02** | **1.73440E-06** | **1.73440E-06** | **1.73440E-06** |
| CLACO | **1.60464E-04** | **4.89690E-04** | **1.73440E-06** | **1.73440E-06** | **1.73440E-06** |
| SCA_PSO | **8.91873E-05** | **2.16302E-05** | 1.00000E+00 | 1.00000E+00 | 1.00000E+00 |
| RDWOA | **1.73440E-06** | 1.77907E-01 | 1.00000E+00 | **1.73440E-06** | 1.00000E+00 |
| OBLGWO | **1.73440E-06** | 5.85712E-01 | **1.73440E-06** | 1.00000E+00 | 1.00000E+00 |
| FSTPSO | **1.73440E-06** | **1.97295E-05** | **1.73440E-06** | **1.73440E-06** | **1.73440E-06** |
|  | F26 | F27 | F28 | F29 | F30 |
| SCADE | **1.73440E-06** | **2.70159E-05** | **2.56308E-06** | **3.78962E-06** | **2.56308E-06** |
| CBA | **2.95746E-03** | **1.73440E-06** | **1.73440E-06** | **1.73440E-06** | **1.73440E-06** |
| RCACO | 6.26828E-02 | **1.73440E-06** | **1.73440E-06** | **1.73440E-06** | **1.73440E-06** |
| m_SCA | **1.92092E-06** | **1.73440E-06** | **1.73440E-06** | **1.73440E-06** | **1.73440E-06** |
| CLACO | 9.58990E-01 | **1.73440E-06** | **1.73440E-06** | **1.73440E-06** | **1.73440E-06** |
| SCA_PSO | **3.18168E-06** | 1.00000E+00 | 1.00000E+00 | **4.00996E-05** | **1.82153E-05** |
| RDWOA | **5.70644E-04** | 2.50000E-01 | 1.00000E+00 | **8.28420E-06** | **1.73440E-06** |
| OBLGWO | **3.11232E-05** | **3.90625E-03** | **1.31834E-04** | **1.73440E-06** | **1.73440E-06** |
| FSTPSO | **1.73440E-06** | **1.73440E-06** | **1.73440E-06** | **1.73440E-06** | **1.73440E-06** |

**Table 7.** Comparison results of SRWPSO with new peer variants

|  | F1 |  | F2 |  | F3 |  |
| --- | --- | --- | --- | --- | --- | --- |
|  | AVG | STD | AVG | STD | AVG | STD |
| SRWPSO | **2.20448E+05** | **2.87090E+05** | **1.37229E+03** | **6.28997E+03** | **3.00136E+02** | **1.69843E-01** |
| EWOA | 3.77911E+06 | 3.85627E+06 | 9.31902E+03 | 8.73253E+03 | 4.55493E+03 | 3.69423E+03 |
| EESHHO | 9.59365E+05 | 5.54697E+05 | 9.29158E+03 | 8.31147E+03 | 5.12857E+03 | 2.21899E+03 |
| XMACOR | 6.05833E+05 | 4.73891E+05 | 1.49729E+04 | 1.20632E+04 | 5.54893E+03 | 6.53514E+03 |
| CAGWO | 3.91097E+07 | 1.88104E+07 | 4.77227E+08 | 3.65554E+08 | 2.78325E+04 | 5.51839E+03 |
| SGLSCA | 2.91976E+06 | 1.25313E+06 | 1.55654E+04 | 7.76589E+03 | 5.98259E+03 | 2.38240E+03 |
| IGWO | 1.58627E+07 | 5.84651E+06 | 2.77400E+06 | 1.64997E+06 | 6.67546E+03 | 2.93305E+03 |
| GCHHO | 2.12275E+06 | 1.04549E+06 | 1.31852E+04 | 8.52404E+03 | 2.34199E+03 | 1.68318E+03 |
|  | F4 |  | F5 |  | F6 |  |
|  | AVG | STD | AVG | STD | AVG | STD |
| SRWPSO | 4.61244E+02 | **2.25573E+01** | **5.20001E+02** | **2.63497E-03** | 6.18684E+02 | 2.76865E+00 |
| EWOA | 5.16594E+02 | 4.90846E+01 | 5.20112E+02 | 1.04467E-01 | 6.21855E+02 | 3.57842E+00 |
| EESHHO | 4.89255E+02 | 3.30858E+01 | 5.20020E+02 | 4.35510E-02 | 6.21775E+02 | 3.18384E+00 |
| XMACOR | **4.55141E+02** | 3.43901E+01 | 5.20925E+02 | 5.75344E-02 | **6.03530E+02** | **1.64796E+00** |
| CAGWO | 5.74307E+02 | 2.78241E+01 | 5.21038E+02 | 6.11716E-02 | 6.11164E+02 | 2.23018E+00 |
| SGLSCA | 5.25077E+02 | 4.24238E+01 | 5.20113E+02 | 1.38550E-01 | 6.17339E+02 | 4.90975E+00 |
| IGWO | 5.27625E+02 | 3.16638E+01 | 5.20511E+02 | 1.28578E-01 | 6.19222E+02 | 3.15544E+00 |
| GCHHO | 4.93688E+02 | 3.41188E+01 | 5.20118E+02 | 1.16653E-01 | 6.28536E+02 | 3.06279E+00 |
|  | F7 |  | F8 |  | F9 |  |
|  | AVG | STD | AVG | STD | AVG | STD |
| SRWPSO | 7.00012E+02 | 1.29747E-02 | 8.66331E+02 | 1.39955E+01 | 1.02941E+03 | 2.76816E+01 |
| EWOA | 7.00057E+02 | 6.37283E-02 | **8.34593E+02** | **9.75460E+00** | 1.05274E+03 | 3.31036E+01 |
| EESHHO | 7.00025E+02 | 2.91958E-02 | 8.41696E+02 | 1.87607E+01 | 1.06871E+03 | 2.28901E+01 |
| XMACOR | **7.00005E+02** | **8.42307E-03** | 9.08486E+02 | 4.38426E+01 | 1.08162E+03 | 2.43644E+01 |
| CAGWO | 7.04556E+02 | 1.91824E+00 | 9.10536E+02 | 4.19879E+01 | 1.06727E+03 | 3.42925E+01 |
| SGLSCA | 7.00007E+02 | 9.10411E-03 | 8.79640E+02 | 1.95633E+01 | 1.03426E+03 | 4.07487E+01 |
| IGWO | 7.00979E+02 | 7.13378E-02 | 8.85279E+02 | 1.53582E+01 | **1.01481E+03** | 2.32532E+01 |
| GCHHO | 7.00035E+02 | 3.73298E-02 | 8.94460E+02 | 2.01915E+01 | 1.07903E+03 | **2.09139E+01** |
|  | F10 |  | F11 |  | F12 |  |
|  | AVG | STD | AVG | STD | AVG | STD |
| SRWPSO | 2.54862E+03 | 4.52441E+02 | **3.63283E+03** | 5.12786E+02 | 1.20021E+03 | **6.54336E-02** |
| EWOA | 1.81996E+03 | 3.33031E+02 | 4.69069E+03 | 4.77125E+02 | 1.20038E+03 | 1.41571E-01 |
| EESHHO | **1.68501E+03** | **2.93588E+02** | 4.13446E+03 | 4.91801E+02 | **1.20019E+03** | 7.37318E-02 |
| XMACOR | 1.86323E+03 | 9.78108E+02 | 7.39417E+03 | 4.76842E+02 | 1.20243E+03 | 2.52893E-01 |
| CAGWO | 5.36578E+03 | 1.51413E+03 | 6.85882E+03 | 1.36010E+03 | 1.20295E+03 | 3.82054E-01 |
| SGLSCA | 2.21709E+03 | 6.04698E+02 | 4.23157E+03 | 6.28017E+02 | 1.20024E+03 | 1.75330E-01 |
| IGWO | 3.50936E+03 | 5.42606E+02 | 4.34663E+03 | 8.01369E+02 | 1.20062E+03 | 2.81427E-01 |
| GCHHO | 2.67164E+03 | 5.64662E+02 | 5.21496E+03 | **4.32528E+02** | 1.20086E+03 | 3.70874E-01 |
|  | F13 |  | F14 |  | F15 |  |
|  | AVG | STD | AVG | STD | AVG | STD |
| SRWPSO | **1.30034E+03** | 7.72204E-02 | **1.40025E+03** | 1.03089E-01 | **1.50629E+03** | 3.03541E+00 |
| EWOA | 1.30052E+03 | 1.03739E-01 | 1.40031E+03 | **3.92410E-02** | 1.52086E+03 | 9.12168E+00 |
| EESHHO | 1.30056E+03 | 1.11403E-01 | 1.40034E+03 | 1.75465E-01 | 1.51792E+03 | 6.47672E+00 |
| XMACOR | 1.30040E+03 | 6.34690E-02 | 1.40029E+03 | 4.46011E-02 | 1.51617E+03 | 1.94153E+00 |
| CAGWO | 1.30039E+03 | **5.33994E-02** | 1.40043E+03 | 1.38312E-01 | 1.51828E+03 | 2.64787E+00 |
| SGLSCA | 1.30040E+03 | 9.14959E-02 | 1.40027E+03 | 4.61609E-02 | 1.50740E+03 | **1.89913E+00** |
| IGWO | 1.30059E+03 | 1.04650E-01 | 1.40050E+03 | 3.59251E-01 | 1.51712E+03 | 3.90194E+00 |
| GCHHO | 1.30054E+03 | 1.12199E-01 | 1.40031E+03 | 1.85770E-01 | 1.52988E+03 | 1.22888E+01 |
|  | F16 |  | F17 |  | F18 |  |
|  | AVG | STD | AVG | STD | AVG | STD |
| SRWPSO | **1.61045E+03** | 7.58321E-01 | **2.96256E+04** | **1.48848E+04** | 4.56009E+03 | 2.55704E+03 |
| EWOA | 1.61169E+03 | 5.03205E-01 | 8.57049E+05 | 6.34016E+05 | 6.61853E+03 | 5.38599E+03 |
| EESHHO | 1.61059E+03 | 6.56598E-01 | 4.55033E+05 | 2.43144E+05 | 4.13864E+03 | 2.42336E+03 |
| XMACOR | 1.61211E+03 | **3.35325E-01** | 1.05070E+05 | 7.86695E+04 | 3.51571E+03 | 2.41474E+03 |
| CAGWO | 1.61187E+03 | 4.24073E-01 | 1.15900E+06 | 8.68385E+05 | 6.95598E+05 | 2.44332E+06 |
| SGLSCA | 1.61094E+03 | 5.88897E-01 | 2.96924E+05 | 2.07544E+05 | **3.07045E+03** | **1.04501E+03** |
| IGWO | 1.61174E+03 | 6.83250E-01 | 8.73137E+05 | 5.32527E+05 | 1.71485E+04 | 2.09406E+04 |
| GCHHO | 1.61210E+03 | 4.89211E-01 | 5.23701E+05 | 3.26469E+05 | 5.73472E+03 | 6.10596E+03 |
|  | F19 |  | F20 |  | F21 |  |
|  | AVG | STD | AVG | STD | AVG | STD |
| SRWPSO | 1.92156E+03 | 2.00853E+01 | **2.23908E+03** | **1.12818E+02** | **4.96345E+04** | 1.32920E+05 |
| EWOA | 1.91764E+03 | 2.47467E+01 | 4.42457E+03 | 2.49862E+03 | 4.03995E+05 | 3.21306E+05 |
| EESHHO | 1.91640E+03 | 1.92848E+01 | 3.98579E+03 | 2.10220E+03 | 2.81665E+05 | 2.24203E+05 |
| XMACOR | **1.90750E+03** | **1.58986E+00** | 2.93949E+03 | 9.33181E+02 | 9.54336E+04 | 9.21536E+04 |
| CAGWO | 1.92868E+03 | 5.82521E+00 | 2.08323E+04 | 8.38732E+03 | 2.28648E+05 | 2.42083E+05 |
| SGLSCA | 1.91234E+03 | 1.65681E+00 | 3.43044E+03 | 6.79876E+02 | 1.03752E+05 | **7.07263E+04** |
| IGWO | 1.91867E+03 | 1.25967E+01 | 3.32121E+03 | 1.24314E+03 | 3.16001E+05 | 2.13184E+05 |
| GCHHO | 1.91829E+03 | 2.20209E+01 | 3.36446E+03 | 1.10287E+03 | 2.56198E+05 | 1.90245E+05 |
|  | F22 |  | F23 |  | F24 |  |
|  | AVG | STD | AVG | STD | AVG | STD |
| SRWPSO | 2.65262E+03 | 2.00269E+02 | **2.50000E+03** | **0.00000E+00** | **2.60000E+03** | **0.00000E+00** |
| EWOA | 2.78867E+03 | 1.55606E+02 | 2.61535E+03 | 3.81719E-01 | 2.60433E+03 | 1.02404E+01 |
| EESHHO | 2.71160E+03 | **1.32138E+02** | 2.50000E+03 | 0.00000E+00 | 2.60000E+03 | 6.03495E-05 |
| XMACOR | **2.41829E+03** | 1.51484E+02 | 2.61524E+03 | 2.64057E-11 | 2.62303E+03 | 6.19825E+00 |
| CAGWO | 2.63798E+03 | 1.87356E+02 | 2.62572E+03 | 3.91096E+00 | 2.60000E+03 | 8.87158E-05 |
| SGLSCA | 2.62136E+03 | 1.40061E+02 | 2.50000E+03 | 0.00000E+00 | 2.60000E+03 | 0.00000E+00 |
| IGWO | 2.62487E+03 | 1.64983E+02 | 2.62005E+03 | 2.32733E+00 | 2.60001E+03 | 5.66569E-03 |
| GCHHO | 2.80312E+03 | 1.96200E+02 | 2.50000E+03 | 0.00000E+00 | 2.60000E+03 | 8.00889E-05 |
|  | F25 |  | F26 |  | F27 |  |
|  | AVG | STD | AVG | STD | AVG | STD |
| SRWPSO | **2.70000E+03** | **0.00000E+00** | **2.70035E+03** | 1.10577E-01 | **2.90000E+03** | **0.00000E+00** |
| EWOA | 2.71503E+03 | 6.33367E+00 | 2.72382E+03 | 4.28854E+01 | 3.60924E+03 | 2.28810E+02 |
| EESHHO | 2.70000E+03 | 0.00000E+00 | 2.74031E+03 | 4.95692E+01 | 2.90000E+03 | 0.00000E+00 |
| XMACOR | 2.70442E+03 | 1.46291E+00 | 2.76020E+03 | 4.96390E+01 | 3.11216E+03 | 5.01264E+01 |
| CAGWO | 2.70000E+03 | 0.00000E+00 | 2.71455E+03 | 3.17449E+01 | 3.26015E+03 | 5.89858E+01 |
| SGLSCA | 2.70000E+03 | 0.00000E+00 | 2.70039E+03 | 1.12030E-01 | 2.90000E+03 | 0.00000E+00 |
| IGWO | 2.71001E+03 | 2.89322E+00 | 2.70076E+03 | 1.48890E-01 | 3.11764E+03 | 4.86474E+01 |
| GCHHO | 2.70000E+03 | 0.00000E+00 | 2.70047E+03 | **1.04323E-01** | 2.90000E+03 | 0.00000E+00 |
|  | F28 |  | F29 |  | F30 |  |
|  | AVG | STD | AVG | STD | AVG | STD |
| SRWPSO | **3.00000E+03** | **0.00000E+00** | **3.10000E+03** | **0.00000E+00** | **3.20000E+03** | **0.00000E+00** |
| EWOA | 4.40825E+03 | 4.56564E+02 | 5.07659E+06 | 4.56671E+06 | 1.01104E+04 | 5.51084E+03 |
| EESHHO | 3.00000E+03 | 0.00000E+00 | 3.96923E+03 | 9.89952E+02 | 6.18099E+03 | 1.37456E+03 |
| XMACOR | 3.74405E+03 | 2.01858E+02 | 1.51428E+05 | 6.62801E+05 | 6.86789E+03 | 2.52425E+03 |
| CAGWO | 3.72605E+03 | 4.24844E+01 | 8.25471E+04 | 1.52143E+05 | 3.70907E+04 | 1.59395E+04 |
| SGLSCA | 3.00000E+03 | 0.00000E+00 | 3.10000E+03 | 0.00000E+00 | 3.20000E+03 | 0.00000E+00 |
| IGWO | 3.87863E+03 | 2.31998E+02 | 7.41867E+05 | 2.76922E+06 | 2.30169E+04 | 8.74754E+03 |
| GCHHO | 3.00000E+03 | 0.00000E+00 | 2.89109E+06 | 4.15368E+06 | 7.25682E+03 | 1.65679E+03 |

**Table 8.** The p-value of the comparison result of SRWPSO with new peer variants

|  | F1 | F2 | F3 | F4 | F5 |
| --- | --- | --- | --- | --- | --- |
| EWOA | 1.73440E-06 | 2.59671E-05 | 1.73440E-06 | 1.02463E-05 | 1.73440E-06 |
| EESHHO | 7.69086E-06 | 3.40526E-05 | 1.73440E-06 | 6.63921E-04 | 1.24526E-02 |
| XMACOR | 6.31976E-05 | 1.79885E-05 | 1.73440E-06 | **6.87136E-02** | 1.73440E-06 |
| CAGWO | 1.73440E-06 | 1.73440E-06 | 1.73440E-06 | 1.73440E-06 | 1.73440E-06 |
| SGLSCA | 1.73440E-06 | 3.88218E-06 | 1.73440E-06 | 2.87860E-06 | 1.35948E-04 |
| IGWO | 1.73440E-06 | 1.73440E-06 | 1.73440E-06 | 2.35342E-06 | 1.73440E-06 |
| GCHHO | 1.73440E-06 | 2.60333E-06 | 1.73440E-06 | 1.79885E-05 | 1.36011E-05 |
|  | F6 | F7 | F8 | F9 | F10 |
| EWOA | 4.53356E-04 | 1.02463E-05 | 1.73440E-06 | 1.56585E-02 | 9.31566E-06 |
| EESHHO | 6.15641E-04 | **1.30592E-01** | 3.88111E-04 | 4.44934E-05 | 3.88218E-06 |
| XMACOR | 1.73440E-06 | 1.10917E-02 | 1.74228E-04 | 1.23808E-05 | 1.04444E-02 |
| CAGWO | 2.12664E-06 | 1.73440E-06 | 6.89229E-05 | 1.60464E-04 | 2.12664E-06 |
| SGLSCA | **3.38856E-01** | **1.58855E-01** | 1.83258E-03 | **7.03564E-01** | 2.18267E-02 |
| IGWO | **8.13017E-01** | 1.73440E-06 | 1.14992E-04 | 4.27667E-02 | 9.31566E-06 |
| GCHHO | 1.73440E-06 | 5.66717E-03 | 3.18168E-06 | 3.18168E-06 | **6.28843E-01** |
|  | F11 | F12 | F13 | F14 | F15 |
| EWOA | 2.87860E-06 | 3.51524E-06 | 5.21649E-06 | 3.58884E-04 | 1.73440E-06 |
| EESHHO | 4.11403E-03 | **1.84622E-01** | 5.75165E-06 | 2.76527E-03 | 1.73440E-06 |
| XMACOR | 1.73440E-06 | 1.73440E-06 | 2.95746E-03 | 1.31942E-02 | 1.73440E-06 |
| CAGWO | 2.12664E-06 | 1.73440E-06 | 1.95692E-02 | 1.05695E-04 | 1.73440E-06 |
| SGLSCA | 1.38204E-03 | **7.65519E-01** | 1.31942E-02 | **1.25438E-01** | 3.68261E-02 |
| IGWO | 3.06500E-04 | 1.92092E-06 | 1.73440E-06 | 4.53356E-04 | 2.35342E-06 |
| GCHHO | 1.73440E-06 | 1.73440E-06 | 8.46608E-06 | **6.56411E-02** | 1.73440E-06 |
|  | F16 | F17 | F18 | F19 | F20 |
| EWOA | 9.31566E-06 | 1.73440E-06 | **1.52861E-01** | 2.95746E-03 | 6.31976E-05 |
| EESHHO | **4.40522E-01** | 1.73440E-06 | **8.13017E-01** | 1.59270E-03 | 5.75165E-06 |
| XMACOR | 1.92092E-06 | 1.12654E-05 | 3.32689E-02 | 1.73440E-06 | 2.16302E-05 |
| CAGWO | 2.35342E-06 | 1.73440E-06 | 1.73440E-06 | 1.47954E-02 | 1.73440E-06 |
| SGLSCA | 1.56585E-02 | 1.92092E-06 | 4.11403E-03 | 7.71217E-04 | 1.73440E-06 |
| IGWO | 1.02463E-05 | 1.73440E-06 | 1.63945E-05 | **2.36936E-01** | 1.73440E-06 |
| GCHHO | 1.73440E-06 | 1.73440E-06 | **7.65519E-01** | **5.85712E-01** | 1.73440E-06 |
|  | F21 | F22 | F23 | F24 | F25 |
| EWOA | 1.97295E-05 | 1.24526E-02 | 1.73440E-06 | 1.73440E-06 | 2.56308E-06 |
| EESHHO | 1.49356E-05 | **1.77907E-01** | **1.00000E+00** | 1.96437E-04 | **1.00000E+00** |
| XMACOR | 1.47728E-04 | 3.40526E-05 | 6.79885E-08 | 1.73440E-06 | 1.73440E-06 |
| CAGWO | 2.59671E-05 | **8.13017E-01** | 1.73440E-06 | 1.73440E-06 | **1.00000E+00** |
| SGLSCA | 1.35948E-04 | **3.38856E-01** | **1.00000E+00** | **1.00000E+00** | **1.00000E+00** |
| IGWO | 1.63945E-05 | **6.58331E-01** | 1.73440E-06 | 1.73440E-06 | 2.56308E-06 |
| GCHHO | 1.12654E-05 | 1.47954E-02 | **1.00000E+00** | 1.82153E-05 | **1.00000E+00** |
|  | F26 | F27 | F28 | F29 | F30 |
| EWOA | 5.75165E-06 | 1.73440E-06 | 1.73440E-06 | 1.67206E-06 | 1.73440E-06 |
| EESHHO | 2.12664E-06 | **1.00000E+00** | **1.00000E+00** | 2.93053E-04 | 2.56308E-06 |
| XMACOR | 1.49356E-05 | 1.73440E-06 | 1.73440E-06 | 1.73440E-06 | 1.73440E-06 |
| CAGWO | 3.85424E-03 | 1.73440E-06 | 1.73440E-06 | 1.73440E-06 | 1.73440E-06 |
| SGLSCA | **8.22065E-02** | **1.00000E+00** | **1.00000E+00** | **1.00000E+00** | **1.00000E+00** |
| IGWO | 2.60333E-06 | 1.73440E-06 | 1.73440E-06 | 1.73440E-06 | 1.73440E-06 |
| GCHHO | 2.41180E-04 | **1.00000E+00** | **1.00000E+00** | 2.54416E-06 | 2.56308E-06 |
